# 12th CONSEURO and the 25th National and 12th International SEOC Congress, April 3-5, 2025, Cordoba, Spain

**DOI:** 10.4317/jced.1122335667806

**Published:** 2025-12-20

Abstracts of the scientific communications follow.

