# Supplementary material for: 12th CONSEURO and the 25th National and 12th International SEOC Congress, April 3-5, 2025, Cordoba, Spain
Source: J Clin Exp Dent. 2025 Dec 20;17(Suppl 2):S1–S83. doi: 10.4317/jced.1122335667806 (PMC12718635; doi:10.4317/jced.1122335667806)
Supplement: Related texts

# Abstract: 01. Non-surgical retreatment of tooth 4.6

**Authors:** S. Wu

**Affiliations:** *International University of Catalonia, Barcelona, Spain. stephanie53591@gmail.com *

## Aim

This case report aims to describe the non-surgical endodontic retreatment and subsequent indirect overlay restoration of a mandibular first molar (tooth 4.6) presenting with symptomatic apical periodontitis following previous endodontic treatment.

## Case Report

A 56-year-old male patient presented to the Universitat Internacional de Catalunya clinic reporting "great pain when biting in the lower right molars". His medical history was non-contributory, with no known allergies, and he was classified as ASA I according to the American Society of Anesthesiologists. Clinical examination revealed pain upon percussion and palpation of the lower right first molar (tooth 4.6), with physiological mobility and periodontal probing depths, and no visible sinus tract. Pre-operative periapical radiography confirmed a previously endodontically treated tooth. Based on the American Association of Endodontists' criteria, the pulpal diagnosis was 'Previously treated tooth' and the periapical diagnosis was 'Symptomatic apical periodontitis'. The proposed treatment plan involved non-surgical endodontic retreatment followed by an indirect overlay restoration. During the retreatment, two canals were located. Gutta-percha was removed using a Reciproc M-file 25.08. Patency was established with a manual #10 K-file. The mesial canal had a working length of 26 mm with an apical calibration of 40.02, while the distal canal measured 21 mm with an apical calibration of 55.02, both achieved with manual K-files. Prior to final obturation, a thorough irrigation protocol was performed sequentially: 4.25% sodium hypochlorite, followed by 10% citric acid, then another rinse with 4.25% sodium hypochlorite, and finally 96% alcohol.

## Results

The case was followed up for 6 months. The follow-up radiograph demonstrated healing of the periapical lesion. Clinically, the tooth remained asymptomatic, with no pain upon percussion or palpation, and maintained physiological mobility and periodontal probing depths.

## Discussion

This case highlights the principles of minimally invasive dentistry (MID), prioritizing tooth structure preservation through non-surgical retreatment and direct/indirect restorative approaches. The choice of an indirect overlay restoration is consistent with modern restorative concepts. The observed healing of the periapical lesion and resolution of symptoms at 6 months aligns with successful outcomes reported for non-surgical endodontic retreatments in the presence of periapical lesions

## Conclusions

This case report demonstrates that non-surgical endodontic retreatment combined with an indirect overlay restoration can successfully manage symptomatic apical periodontitis in a previously treated mandibular molar, leading to periapical healing and complete symptom resolution within 6 months. This conservative interdisciplinary approach offers a predictable and effective option for the functional rehabilitation and preservation of compromised teeth.

# Abstract: 02. Title: Endodontic management of tooth 4.5 through a bridge

**Authors:** S. Bóveda

**Affiliations:** *International University of Catalonia, Barcelona, Spain. sofiaboveda6@gmail.com*

## Aim

This case report aims to describe the non-surgical endodontic treatment of a mandibular second premolar (tooth 4.5) presenting with pulp necrosis and symptomatic apical periodontitis. The primary objective was to successfully manage the infection while preserving the integrity of an existing, well-adapted, and leakage-free prosthetic bridge of which tooth 4.5 was an abutment.

## Case Report

A patient presented to the Universitat Internacional de Catalunya clinic with pulp necrosis and symptomatic apical periodontitis affecting tooth 4.5. This tooth served as an abutment for a prosthetic bridge extending from tooth 4.5 to 4.3. Clinical and radiographic examination confirmed the diagnosis. Given the good adaptation, absence of leakage, and satisfactory aesthetics of the existing bridge, the treatment plan prioritized a non-surgical root canal treatment performed through the bridge. A preoperative Cone-Beam Computed Tomography (CBCT) scan was obtained to comprehensively assess the root canal anatomy and the extent of the periapical infection. Instrumentation of the root canal was performed using Protaper Ultimate and ProFile files which are well-established systems for root canal preparation. Following thorough chemomechanical debridement, obturation was completed using the Guttacore system, an obturation technique known for its ability to fill the canal space effectively.

## Results

The non-surgical root canal treatment of tooth 4.5 through the existing bridge was successfully completed, and the root canal was obturated. Postoperative controls, including percussion tests and radiographic evaluations, are scheduled at 3-6 months.

## Discussion

This case highlights a practical and conservative approach to managing endodontic pathology in a tooth serving as an abutment for a fixed prosthetic restoration. The decision to perform non-surgical root canal treatment through the bridge was crucial, prioritizing the preservation of the existing, functional, and aesthetically acceptable prosthesis. This aligns with the broader principle of minimally invasive dentistry. The utilization of preoperative CBCT was instrumental in precisely delineating the complex root canal anatomy and confirming the extent of the periapical pathology.

## Conclusions

This case report illustrates that the non-surgical endodontic treatment of a tooth through an existing prosthetic bridge is a viable and conservative option for managing pulp necrosis and symptomatic apical periodontitis. This interdisciplinary approach, supported by advanced diagnostic tools like CBCT and modern endodontic techniques, offers a predictable path to preserve compromised teeth and their prosthetic restorations, thereby fulfilling both functional and aesthetic patient expectations. Ongoing clinical and radiographic follow-up is essential to confirm long-term healing.

# Abstract: 03. Endodontic restoration of a tooth with paracrestal perforations: a case report

**Authors:** M. Tarabay

**Affiliations:** *International University of Catalonia, Barcelona, Spain. mohamadtarabay@uic.es*

## Aim

A 52-year-old female patient with no relevant medical history presented seeking dental restoration. Clinical and radiographic examinations revealed paracrestal perforations and challenges in locating the root canals. Differential diagnosis considered external root resorption, untreated root canals, and perforation-related complications. The primary treatment objective was to preserve the affected tooth through successful perforation sealing, comprehensive endodontic therapy, and a definitive prosthetic restoration.

## Case Report

Upon access cavity preparation, two perforations, approximately the size of a #10 file, were observed. While the vestibular canal was readily identified, localization and achieving permeability of the palatal canal necessitated manual scouting, ultrasonic instrumentation, and the use of a reciprocating file. Hemorrhage at the perforation site was controlled with sodium hypochlorite irrigation, followed by drying and sealing with mineral trioxide aggregate (MTA). Root canal treatment was completed in a subsequent session following standard endodontic procedures, including working length determination, conometry, and obturation. A provisional restoration was placed. Radiographic evaluation confirmed adequate sealing of the perforation with a calcium silicate-based hydraulic cement. The patient remained asymptomatic at a two-month follow-up, facilitating the placement of a definitive lithium disilicate crown for tooth 1.4. An 8-month follow-up affirmed the successful outcome with no reported complications.

## Discussion

The integration of endodontic and prosthetic treatment was key to achieving long-term functional and esthetic success . This interdisciplinary approach is a cornerstone in modern dentistry, particularly in complex cases requiring a confluence of specialized techniques. The definitive restoration with a lithium disilicate crown highlights the use of high-strength ceramic materials that provide not only excellent esthetics but also long-term stability and adhesive properties, crucial for functional outcomes.

## Conclusions

This case highlights the importance of meticulous diagnosis and treatment planning in endodontic cases with perforations. Utilizing CBCT for accurate assessment and MTA for perforation repair played a crucial role in preserving the tooth. The integration of endodontic and prosthetic treatment ensured long-term functional and esthetic success. At the 8-month follow-up, the patient exhibited complete symptom resolution and a well-integrated final restoration. This approach reinforces the viability of conservative management in complex endodontic cases, preserving the patient’s natural dentition with predictable outcomes.

# Abstract: 04. Root canal retreatment of tooth 2.5: diagnosis, treatment, and outcomes

**Authors:** A.K. Zakarneh

**Affiliations:** *International University of Catalonia, Barcelona, Spain. ahmadkamal99@windowslive.com *

## Aim

This case report details the successful non-surgical endodontic retreatment of tooth 2.5 in a 42-year-old female patient diagnosed with a chronic apical abscess and a previously treated root canal. The primary objective was to resolve the periapical pathology and restore the tooth's function and aesthetics. Cone-beam computed tomography (CBCT) was strategically utilized for comprehensive diagnostic evaluation, confirming the extent of the lesion and guiding the retreatment process.

## Case Report

Initial clinical examination revealed a chronic apical abscess, necessitating retreatment. At the first appointment, the existing post was carefully removed. De-obturation of the root canal system was meticulously performed using ProFile 25.04 rotary files. Calcium hydroxide was then placed as an intracanal medicament to promote microbial control. During the second visit, a significant reduction in the sinus tract was observed. Complete gutta-percha removal and accurate working length verification were achieved. The irrigation protocol involved a 4.25% sodium hypochlorite (NaOCl) solution, activated with Passive Ultrasonic Irrigation (PUI), followed by 10% citric acid for effective smear layer removal, and a final rinse with NaOCl and 98% alcohol. Root canal obturation was completed using cone fit verification, downpack, and backfill techniques, confirmed by radiographic imaging. The tooth was subsequently restored with a Class II composite build-up.

## Results

At the follow-up examinations, complete resolution of the sinus tract and absence of periapical pathology were confirmed both clinically and radiographically. The patient remained asymptomatic, indicating a successful retreatment outcome.

## Conclusions

The case highlights the value of precise technique, proper case selection, and adherence to evidence-based protocols in achieving favorable outcomes in root canal retreatment. Effective post removal, thorough canal disinfection, and high-quality obturation were paramount to achieving the favorable clinical outcome. The enhancement of irrigation efficacy with PUI significantly contributed to the thorough decontamination of the root canal system. The use of calcium hydroxide also supported sustained microbial control. Coronal sealing with a definitive composite restoration further ensured the long-term success and integrity of the treated tooth. While non-surgical retreatment proved effective and represents a minimally invasive strategy for preserving natural dentition, alternative interventions such as apical surgery or extraction could have been considered if healing had not progressed as favorably.

# Abstract: 05. Non-surgical retreatment on a 3.6.

**Authors:** P. Solis Zurro, A.M. Avadanei, M. Garcia Font

**Affiliations:** *International University of Catalonia, Barcelona, Spain. pablo.solis@uic.es*

## Aim

This case report describes the successful non-surgical endodontic retreatment of a previously treated tooth, (tooth 3.6), presenting with a chronic apical abscess and a fractured metal-ceramic crown with distal leakage. The objective was to eliminate the periapical infection, restore the tooth's structural integrity, and achieve a favorable long-term prognosis through a comprehensive retreatment and definitive crown restoration. Cone-beam computed tomography (CBCT), was crucial for confirming the diagnosis of periapical pathology associated with the mesial root and guiding the treatment plan.

## Case Report

A patient presented with a gingival sinus tract and a fractured metal-ceramic crown with apparent distal leakage. Clinical examination revealed no pain on percussion or palpation, consistent with a chronic apical abscess. The treatment was performed over three appointments, with resolution of the sinus tract observed between the first and second visits. In the first appointment, the existing metal-ceramic crown was removed, and the carious lesion on the distal wall was thoroughly cleaned. A metallic post was removed from the distal root canal, and the existing gutta-percha filling material was removed from the root canals using the Reciproc M-Wire system. In the second appointment, the root canals were precisely calibrated at their working length, followed by obturation with gutta-percha and a resin sealer, employing a warm vertical compaction technique. Subsequently, the tooth was prepared for a new crown, and an intraoral digital scan was performed for fabrication. The third appointment involved the definitive cementation of the new ceramic crown under rubber dam isolation using warm composite for optimal adaptation and seal.

## Results

Follow-up examinations at 3, 6, and 12 months demonstrated complete resolution of the clinical signs of periapical disease, including the sinus tract, and radiographic evidence of periapical healing.

## Conclusions

This case highlights the efficacy of a systematic non-surgical endodontic retreatment combined with meticulous restorative procedures for managing chronic apical abscesses in previously treated teeth. Thorough removal of defective restorations and existing root canal filling material, precise working length determination, and high-quality obturation are paramount for successful outcomes. The M-Wire system facilitated efficient removal of the gutta-percha, contributing to comprehensive canal debridement. This conservative approach proved effective in preserving the natural tooth structure and function, reinforcing its viability as a primary management strategy in complex endodontic scenarios.

# Abstract: 06. Non-surgical management in an immature maxillary central incisor with open apex using mineral trioxide aggregate (mta) apical barrier: a case report.

**Authors:** S. Jiménez

**Affiliations:** *International University of Catalonia, Barcelona, Spain. s.jimenez@uic.es*

## Aim

This case report details the successful non-surgical management of a 30-year-old female patient presenting with localized dental pain in tooth #2.1, diagnosed with pulp necrosis and symptomatic apical periodontitis. The case was complicated by the presence of an open apex, consistent with an immature root, and a history of dental trauma. The primary objective was to achieve periapical healing and tooth preservation through an apical barrier technique using Mineral Trioxide Aggregate (MTA).

## Case Report

The patient reports no systemic conditions, allergies, or smoking habits and has no significant oral health risk factors. Her last dental check-up was about seven months ago. She recalls a history of trauma to the affected tooth, which likely set off the current pathology. On clinical evaluation, tooth 2.1 showed a non-responsive result to cold testing, confirming loss of pulp vitality. Percussion testing elicited a positive response, suggesting periapical inflammation, and palpation was also tender, further indicating periapical involvement. The tooth exhibited Grade I mobility but had physiological probing depths, ruling out any significant periodontal breakdown. Radiographic imaging confirmed an open apex, consistent with an immature root. Given these findings, the diagnosis was established as pulp necrosis with symptomatic apical periodontitis. To manage this case predictably, the proposed treatment plan was an apical barrier technique using MTA. Since the apex remained open, traditional obturation wasn’t viable, making MTA the material of choice to create a reliable apical seal. The patient was thoroughly informed about the condition, treatment approach, and prognosis. After a detailed discussion, she provided informed consent, and treatment commenced. The endo protocol involved standard isolation with a rubber dam to maintain asepsis. After access was gained, copious irrigation was performed using sodium hypochlorite to ensure thorough disinfection of the necrotic canal space. Mechanical debridement was limited given the immature root structure, and passive irrigation techniques were emphasized to maximize biofilm disruption. Final irrigation included EDTA to remove the smear layer, followed by a final rinse with CHX for its substantivity. Once the canal space was clean and dry, MTA was carefully placed at the apex to create a barrier, ensuring proper condensation to prevent extrusion beyond the root. A moistened cotton pellet was placed to allow initial setting, and the access was temporized. At the subsequent visit, after verifying the MTA set properly, the canal was obturated using a bioceramic sealer with warm vertical compaction to enhance the apical seal. The access was restored with composite to ensure a coronal seal, minimizing the risk of reinfection. At the one-year follow-up, radiographic assessment revealed significant bone regeneration around the periapex, indicating successful healing. Clinically, the patient remained asymptomatic, with no signs of reinfection or periapical pathology. Mobility remained within normal limits, and soft tissue health was stable. The prognosis for long-term retention of the tooth remained favorable.

## Results

At the one-year follow-up, almost complete healing of the affected bone was observed, and the patient remained free of clinical signs or symptoms. This favorable outcome is consistent with the goals of conservative endodontic therapy for periapical lesion resolution and tooth preservation.

## Conclusions

This case highlights the predictable efficacy of conservative non-surgical treatment in immature teeth with pulp necrosis and an open apex through apical barrier formation with MTA. MTA proved to be a reliable material for inducing apical closure and periapical healing, even in incompletely developed roots. The combination of thorough root canal system disinfection and meticulous coronal restoration is crucial for long-term success and preservation of the natural tooth structure. This minimally invasive approach represents a valuable alternative to more radical options like extraction or apical surgery, supporting the philosophy of maximum tooth structure preservation.

# Abstract: 07. Conservative management of reversible pulpitis in a young permanent molar: partial pulpotomy and direct composite

**Authors:** M. Tyrosvouti

**Affiliations:** *International University of Catalonia, Barcelona, Spain. mariatyro@uic.es*

## Aim

This case report aims to detail the conservative, non-surgical management of a permanent molar using vital pulp therapy (VPT) in a young patient diagnosed with reversible pulpitis, emphasizing the maintenance of pulp vitality. This approach aligns with the principles of minimally invasive dentistry.

## Case Report

A 16-year-old male patient presented with symptoms consistent with reversible pulpitis in the mandibular left first molar (tooth 3.6). Given the patient's young age, preserving pulp vitality was crucial to potentially allow for continued root development and strengthen the tooth against future challenges. Clinical and radiographic evaluations confirmed the diagnosis and guided the treatment plan. Treatment Protocol: A partial pulpotomy was performed on tooth 3.6. Standard isolation with a rubber dam was rigorously maintained throughout the procedure to ensure an aseptic field and optimal infection control. The inflamed coronal pulp tissue was carefully removed. Subsequently, a biocompatible pulp-capping material was applied directly to the remaining vital pulp, a technique that has shown promise in promoting pulp healing. The tooth was then definitively restored with a direct composite restoration. This restorative choice is widely utilized for its ability to restore proper function, ensure coronal seal, and prevent bacterial infiltration, which is vital for long-term success.

## Results

At the 6-month follow-up, the patient remained asymptomatic, indicating successful resolution of the pulpitis. Radiographic evaluation demonstrated the preservation of normal periapical tissues, the absence of root resorption or other pathological changes, and the possible formation of a dentin bridge at the pulpotomy site, a direct histological sign of pulp healing and vitality maintenance. This outcome successfully supported the efficacy of partial pulpotomy in maintaining pulp vitality [New Abstract].

## Conclusions

Partial pulpotomy proved to be an effective and conservative approach for managing reversible pulpitis in a young patient. The success of this case highlights the importance of early diagnosis and appropriate intervention in preserving pulp vitality. Long-term follow-up and additional studies are necessary to further validate the long-term outcomes of this treatment modality.

# Abstract: 08. Conservative management of a maxillary first molar with extensive tissue loss: a case report

**Authors:** R.I. Lazo Ribalta

**Affiliations:** *International University of Catalonia, Barcelona, Spain. byo193848@uic.es*

## Objetives

This case report details the non-surgical endodontic and subsequent indirect adhesive restorative management of a maxillary first molar (tooth 1.6) diagnosed with symptomatic irreversible pulpitis and normal periapical tissues, aiming to restore function, address extensive structural loss, and improve long-term prognosis. This approach is consistent with the current emphasis on minimally invasive dentistry.

## Case Report

A patient presented with an extensive old composite restoration and secondary caries reaching the pulp in the maxillary first molar (1.6), necessitating endodontic intervention. Preoperative Cone-Beam Computed Tomography (CBCT) was utilized to meticulously assess the complex root canal morphology and confirm the absence of periapical pathology. Treatment Protocol: Following rigorous rubber dam isolation to ensure an aseptic field, root canal preparation was meticulously performed using the ProTaper Ultimate rotary system. Effective irrigation protocols were employed, involving sodium hypochlorite (NaOCl) and citric acid to thoroughly disinfect the canal system. The canals were then obturated using the Tagger Hybrid Technique, combining warm vertical and lateral compaction with thermoplasticized gutta-percha and a root canal sealer. Given the significant coronal destruction, an indirect adhesive restoration with cuspal coverage was chosen to provide robust long-term functional and biomechanical integrity, aligning with strategies for managing extensive tissue loss. Designed using CAD/CAM technology to ensure optimal fit and preservation of tooth structure.

## Results

Postoperative controls are scheduled at 3 and 6 months to monitor healing. Successful outcomes for endodontic treatment are typically characterized by the patient remaining asymptomatic and radiographic evidence of periapical healing, without signs of resorption. The long-term performance of indirect composite restorations has been shown to be favorable.

## Conclusions

This case demonstrates the successful endodontic and conservative indirect restorative management of a maxillary first molar with symptomatic irreversible pulpitis and extensive coronal destruction. The approach highlights the importance of precise diagnostics and comprehensive treatment planning for maintaining tooth longevity. Long-term follow-up and additional studies are necessary to further validate the sustained success of this treatment modality.

# Abstract: 09. Minimally invasive management of a maxillary central incisor with internal root resorption and apical periodontitis: a case report

**Authors:** N. Morata Rubio

**Affiliations:** *International University of Catalonia. Barcelona, Spain. noeliamoratarubio@uic.es*

## Aim

This case report aims to present the comprehensive endodontic and restorative management of a previously treated maxillary central incisor (tooth 1.1) diagnosed with asymptomatic apical periodontitis and internal root resorption with communication, highlighting a minimally invasive approach.

## Case Report

A 35-year-old patient presented for evaluation of tooth 1.1, which had a history of endodontic treatment. Clinical and radiographic analyses, including a detailed assessment of the lesion, confirmed the diagnosis of internal resorption with communication to the periapical tissues. Treatment Protocol: The treatment involved non-surgical endodontic retreatment of tooth 1.1. Rigorous rubber dam isolation was maintained throughout the procedure The root canal system was thoroughly disinfected using sodium hypochlorite (NaOCl) irrigation and ultrasonic activation to enhance cleaning efficacy. Subsequently, the root canal was definitively sealed with Mineral Trioxide Aggregate (MTA) putty, a biocompatible material recognized for its sealing properties and ability to induce mineral precipitation. To reinforce the root structure, a layer of Vitrebond (a glass ionomer cement) and flowable resin was applied. For the restorative phase, a Biologically Oriented Preparation Technique (BOPT) was performed, aiming to reshape the gingival architecture and achieve optimal coronal proportion and harmony, without the need for surgical techniques. After minimal adjustments to the provisional crown and gingival margin for symmetry with the adjacent tooth 2.1, the definitive restoration was fabricated. This consisted of a zirconia core with a feldspathic veneer, a combination chosen for its superior aesthetics, strength, and long-term functional and biomechanical integrity. Try-in cements from Variolink were utilized to verify aesthetics and fit. Cementation of the zirconia core was performed using the APC and Panavia protocols, ensuring optimal adhesion, while the feldspathic veneer was cemented with Variolink Warm for natural aesthetic integration.

## Results

At the 8-month follow-up, clinical and radiographic evaluations confirmed the successful outcome of the treatment. The patient remained asymptomatic, periapical healing was evident, and the restoration demonstrated optimal aesthetic and functional integration with the surrounding dentition.

## Conclusions

The presented case successfully illustrates the effective non-surgical endodontic retreatment and indirect restorative management of internal root resorption with communication in a permanent central incisor. This approach, integrating advanced endodontic techniques with a conservative BOPT restorative protocol, highlights the importance of precise diagnosis and interdisciplinary treatment planning for maintaining tooth longevity and achieving predictable aesthetic and functional results. Long-term follow-up and further studies are necessary to validate the sustained success of this treatment modality.

# Abstract: 01. Innovative approach to crown lengthening using forced eruption for restoration of tooth #12

**Authors:** M. Al Fartousi

**Affiliations:** *Dental Clinic for Microscopic Dentistry, Karlsruhe, Germany. info@endo-karlsruhe.de*

## Introduction

This case report introduces an innovative approach to crown lengthening using forced eruption for the restoration of tooth #12. Following root canal treatment and the removal of deep caries, controlled forces were applied with extraction forceps to extrude the tooth, achieving a 3-4 mm crown lengthening without bone removal or soft tissue adjustments. The tooth was temporarily stabilized, built up with a post and core, and splinted for two months before final crown restoration. This method offers a faster, conservative alternative to traditional surgical techniques, avoiding the need for orthodontic appliances and ensuring compatibility with adjacent restorations. Forced eruption proves to be an effective and efficient solution in the esthetic zone for teeth with significant structural loss.

## Case Report

Clinically and radiographically, deep marginal caries was diagnosed on tooth #12, which was restored with a crown. After removing the crown and decay, significant tooth structure destruction was revealed, with necrotic pulp exposure and apical radiolucency. A crown lengthening procedure was recommended to achieve a ferrule effect for a post-core and crown restoration, providing a conservative solution compared to extraction and implant placement, with the forced eruption approach indicated for improving the treatment outcome in the esthetic zone. After completing the root canal treatment, an intrasulcular incision was made through the junctional epithelium and supracrestal connective tissue. Controlled forces were applied using extraction forceps to extrude the tooth without extraction. The erupted tooth was temporarily stabilized with widgets, then built up with a post and core and splinted using a TTS for two months. After the retention phase, the splint was removed, and the tooth was restored with a crown. The crown lengthening of 3-4 mm facilitated the ferrule effect, achieving the desired result in a shorter period.

## Conclusions

This method of crown lengthening proves to be a valuable and effective alternative to traditional surgical techniques, offering the advantage of avoiding bone removal or the need for aesthetic soft tissue corrections. Unlike conventional orthodontic approaches, which require the use of appliances and can extend treatment time by one to two weeks, this technique utilizes extraction forceps to accelerate the process. The decision not to use orthodontic tools was driven by the need to address the differences in crown Material of the adjacent teeth, ensuring compatibility with the surrounding restorations. I confirm that there are no sources of funding.

# Abstract: 02. Minimally invasive direct composite injection for post-orthodontic severe anterior tooth wear using bi-layered 3d-printed indices

**Authors:** K. Hosaka, K. Watanabe, E. Tanaka, J. Tagami

**Affiliations:** *Tokushima University, Tokushima, Japan. keiichihosaka@gmail.com*

## Introduction

Post-orthodontic severe anterior tooth wear requires a minimally invasive approach for functional and esthetic rehabilitation. This case report presents a direct composite injection technique using bi-layered 3D-printed indices with a fully digital workflow, enabling controlled composite placement while preserving natural tooth structure and maintaining occlusal stability.

## Case Report

A 25-year-old female underwent orthodontic treatment with corticotomy-assisted maxillary molar intrusion to correct a severe anterior open bite. Myofunctional therapy was performed to improve oral muscle function and tongue posture. Despite these interventions, significant incisal wear remained on the six maxillary anterior teeth (canine to canine), requiring restoration. A fully digital workflow was used to design an ideal occlusal scheme using CAD software (3Shape Dental System, 3Shape A/S) and fabricate bi-layered 3D-printed indices (outer layer: Dima Print Splint Clear, Kulzer; inner layer: IBT-Flex Resin, Formlabs). The rigid outer shell provided stability, while the flexible inner layer ensured precise adaptation. The 3D printing process was optimized to create non-bonded surfaces, allowing easy removal of the index after composite injection. To avoid interproximal bonding, a skip-tooth approach was used, restoring alternating teeth first, followed by the remaining teeth. A one-step universal adhesive system (Clearfil Universal Bond Quick 2, Kuraray Noritake) was applied, followed by the injection of Clearfil Majesty ES Flow Universal (Kuraray Noritake) through the indices. Light curing, finishing, and polishing were performed. At six months, the restorations demonstrated excellent marginal adaptation, stable occlusion, and improved anterior guidance. The patient reported enhanced function and esthetics with no hypersensitivity or restoration failure.

## Conclusions

This is the first reported case utilizing bi-layered 3D-printed indices for minimally invasive composite injection. The technique, combined with the skip-tooth method and a digital workflow, provides a reliable and reproducible solution for anterior tooth wear rehabilitation. This approach represents a significant advancement in adhesive, esthetic, and sustainable restorative dentistry, offering enhanced accuracy, predictability, and clinical efficiency.

# Abstract: 03. Management of complicated crown fracture with biodentine apical plug and direct composite restoration

**Authors:** M.S. Alaqili Muhand, S. Irazusta

**Affiliations:** *International University of Catalonia, Barcelona, Spain. mohannadaq1998@gmail.com*

## Aim

A 9 year old patient came to the University clinic with a chief complaint of broken crown. Upon examining the tooth the tooth was Necrotic with Asymptomatic apical periodontitis.

## Material and Methods

We started the endodontic treatment we accessed the tooth - Immediate pre-endodontic dentin sealing. working length was 20mm. We irrigated and placed Ca(OH)2 as an intracanal medicament, In the second visit after 2 weeks we reassessed and we calibrated the tooth in which we had a calibre matching size 70 manual file with no gutta percha tug back, we proceeded with final irrigating using 5.25% Sodium hypochlorite 10% Citric acid - Alcohol. We obturated the Canal with Biodentine For the restorative part, we did a Class IV restoration under rubber dam isolation. We closed the access for the canal with flowable composite and then proceeded to the aesthetic part. A bevel in the vestibular area was premade with a red bur to smooth the surface and create a better transition between the enamel of the tooth and the last layer of the restoration. We created a palatal shell with Medium Enamel shade from GC Essentia Kit. The next step was placing the dentin shades, in this case we use a Medium Dentin creating and replicating the anatomy of the neighboring tooth. A thin line of OBE composite was placed to recreate the translucency of the incisal edge, typical in young patients. The last layer applied was a Medium Enamel shade, also from the GC Essentia Kit. Everything was light cured in each layer and a final polymerization under glycerin. Characterization was made by using different burs, SoFlex discs, Enhanced Burs by Dentsply Sirona, EVE rotatory and polishing paste.

## Conclusions

We conclude that Complicated crown fractures can be managed with great aesthetic results if proper protocols were followed.

# Abstract: 01. Effect of experience on dental diagnostics with clinical examination - ai-aided imaging

**Authors:** E. Gürler, G. Ozan, G. Güneç, E. Yildiz, K.C. Aydin

**Affiliations:** *Department of Restorative Dentistry, Institute of Graduate Studies in Health Sciences, Istanbul University, Istanbul, Turkey. efegurler@yahoo.com*

## Aim

Currently, efforts are ongoing to develop artificial intelligence (AI) applications not only limited to radiographic evaluation but also for use in clinical decision-making processes. Nevertheless, detailed studies are needed to evaluate the performance of AI applications used in diagnostics concerning radiographic findings. This study aims to compare whether the use of artificial intelligence as a diagnostic tool can affect the diagnostic accuracy of dentists with varying levels of expertise.

## Material and Methods

Patients aged between 18-50, who have applied to the University clinics and agreed to take part in the study, gone through oral examinations with a mirror- a blunt probe and radiographic evaluations by three evaluators with different experience levels: 4th-year student, 2-year, and 10-year dentists. Each evaluator had examined 50 patients clinically and radiographically and then marked the number of specified five parameters (carious teeth, extracted teeth, restorations, root canal treatments, and periapical lesions) to an Excel table on an iPad where evaluators could not interfere with the table of another. Further, patients’ panoramic images submitted to the AI-based program (Dentistoday) and relevant diagnostics of the program had also been recorded to an Excel sheet as well. The R-language was used to conduct statistical analyses. The significance was set at (p<0.05).

## Results

Analysis revealed a significant main effect of comparison indicating that mean differences from AI varied among 4th-year student, 2-year, and 10-year dentists (p = .021). Regarding carious teeth, the scores of 4th-year student showed significantly more variance with AI results (p<0.05). However, the evaluators’ findings regarding extracted teeth, root canal treatments, and periapical lesions were not significantly different (p > 0.05) either within evaluators or with AI.

## Conclusions

In Conclusion, diagnosis of carious lesions showed more variance with AI in less experienced evaluator. Hence, AI results may be more helpful to less experienced practitioners concerning difficult diagnostic tasks such as carious lesions.

# Abstract: 02. Effect of whitening on the aesthetics of three types of stained composites

**Authors:** A. Rodriguez

**Affiliations:** *Catholic University of Valencia, Valencia, Spain. analucia.rodcruz@gmail.com *

## Aim

The aim of this study is to compare the color stability and whitening ability of three types of composites: nanofilled, suprananofilled, and microfilled.

## Material and Methods

For this study, we used three types of composite resin. As a nanofilled material, we used Filtek XTE by 3M; as a suprananofilled composite, Estelite Sigma by Tokuyama; and as a microfilled agent, Renamel by Cosmedent. All composites were in the A2 VITA shade. A total of 60 specimens were prepared with a thickness of 2 mm (following ISO standards) and a diameter of 8 mm. They were immersed in a coffee solution for 30 days and then subjected to a whitening process using Opalescence Boost 40% PF in a single clinical session without light activation. The color measurements were obtained using spectrophotometry before and after each process. Color was expressed using the Lab* color space. The results were analyzed using analysis of variance (ANOVA). The significance levels were set at p<0.05 for staining and p<0.005 for whitening.

## Results

None of the three composites showed a statistically significant difference (p<0.005) regarding the whitening process, so we cannot confirm that our samples were susceptible to discoloration. The suprananofilled material showed significantly lower staining (p<0.05) compared to the other materials, making it the most stain-resistant composite.

## Conclusions

Composites have varying susceptibility to staining depending on the type of filler particle, with suprananofilled composites being the most stain-resistant and nanofilled composites acquiring the most color. Furthermore, the whitening process had a negative effect on all composite resins.

# Abstract: 01. Effect of artificial toothbrushing on color stability of CAD/CAM-milled and 3D printed composite resins

**Authors:** R. Çoban, S. Atasoy

**Affiliations:** *Department of Restorative Dentistry, Faculty of Dentistry, Ordu University, Altınordu/Ordu, Turkey. rabiamesinci@hotmail.com*

## Aim

The aim of this study was to evaluate the color stability of CAD/CAM-milled and 3D-printed composite resins after artificial toothbrushing. The null hypotheses were that there would be no difference in color stability after toothbrushing between (1) different manufacturing types and (2) different 3D printed resins.

## Material and Methods

Two 3D printed permanent crown resins (Crowntec, CT and VarseoSmile Crown Plus, VS) and a CAD/CAM resin nanoceramic (Cerasmart, CS) were used to fabricate 30 disc-shaped samples (10 × 2 mm) (n=10). The color coordinates at baseline and after subjected to 10.000 cycles of artificial toothbrushing were measured using a spectrophotometer (VITA Easy ShadeV; VITA Zahnfabrik). Color difference (ΔE00) values were calculated by CIEDE2000 and the data were analyzed using 1-way ANOVA and post-hoc Tukey tests (α=.05).

## Results

Manufacturing type affected the ΔE00 values after toothbrushing (p<0.001). The highest ΔE00 values was found in 3D printed CT, while the lowest ΔE00 values was found in CAD/CAM milled CS (p<0.001). No statistically significant difference was found between the ΔE00 values of the different 3D printed resins (p=0.703). Furthermore, a decrease in L*, a* and b* color coordinate values were found after toothbrushing in all groups.

## Conclusions

3D printed composite resins can be considered more susceptible to color change after artificial toothbrushing than CAD/CAM milled resin nanoceramic, although the observed values did not exceed the previously reported acceptability threshold.

# Abstract: 02. Pilot study on the match between style italiano's optishade and the mobile applications chromatcher and denthue

**Authors:** S. Rodriguez Ebert, G. Ionata, I. Reche, M. Valor

**Affiliations:** *Alfonso X el Sabio University, Madrid, Spain. srodrebe@myuax.com*

## Aim

This pilot study aims to evaluate the accuracy and concordance between Style Italian Optishade colorimeter and two mobile applications: Chromatcher and Denthue.

## Material and Methods

An in vitro study was performed, using a sample of six anterior tooth tablets of VITA MFT. Under light conditions of 5000K-6000K, measuring each tooth three times. We assessed the repetition, accuracy, and concordance of these devices.

## Results

Chromatcher presented the highest accuracy with 91.7 %, followed by Optishade with 72.2 % and Denthue with 41.7 %. Significant agreement was found between Optishade and Denthue, although with a low Kappa coefficient. No significant agreement was observed between Optishade and Chromatcher.

## Conclusions

Chromatcher exhibited superior accuracy, suggesting that it is a reliable tool for clinical use. Optishade, although less precise, still gives satisfactory results. However, Denthue does not present satisfactory reliability. There is no concordance between Optishade and Chromatcher, but there is, albeit a low one, between Denthue and Optishade.

# Abstract: 03. A spectrophotometric analysis of mouthwash-induced color changes in fluoride-releasing restorative materials

**Authors:** B. Kesgin, F. Yılmaz

**Affiliations:** *Department of Restorative Dentistry, Faculty of Dentistry, Muğla Sıtkı Koçman University, Muğla, Turkey. busekesgin@gmail.com*

## Aim

Fluoride-releasing resin-based restorative Material are widely used in dentistry due to their aesthetic properties and caries-preventive effects. This study aimed to evaluate the effects of different mouthwashes on the color stability of giomer, glass hybrid, and compomer materials.

## Material and Methods

A total of 120 disk-shaped specimens (n=10) were prepared from three different restorative materials: a giomer, a glass hybrid, and a compomer. The specimens were immersed in three commercially available mouthwashes (Listerine Total Care, Colgate Plax, and Kloroben) and distilled water as a control. Their initial color values (L, a, b*) were recorded using a spectrophotometer (Exact, X-Rite, Inc., Grand Rapids, Michigan, USA), and color measurements were repeated after 24 hours, simulating two years of daily use. The pH values of the three different solutions were measured using a pH meter. One-Way and Two-Way ANOVA were performed to assess the effects of restorative material type and mouthwash on color stability, followed by Tukey’s post-hoc test for multiple comparisons.

## Results

The results showed that the type of restorative material did not significantly affect color stability (p>0.05), while the mouthwash composition had a statistically significant impact (p<0.05). Among the tested solutions, each mouthwash caused different levels of discoloration, with the Plax solution leading to significantly higher color changes compared to Distilled water (p<0.05). The mean ΔE values in all groups were found to be below the clinically acceptable limit of 3.3.

## Conclusions

Mouthwashes have been shown to induce perceptible color alterations in restorative materials, primarily due to their pH and possible interactions with resin components. These findings suggest that both material and mouthwash play a critical role in maintaining the long-term aesthetic quality of dental restorations. Clinicians should consider the staining potential of mouthwashes when recommending oral hygiene products to patients. Further studies with long-term clinical evaluations are necessary to confirm these results.

# Abstract: 04. Water sorption behavior of 3D printed crown Material and conventional nanohybrid composites: a comparative study

**Authors:** Z.I. Metin, S. Akarsu

**Affiliations:** *Department of Restorative Dentistry, Faculty of Dentistry, Ordu University, Altınordu/Ordu, Turkey. z.i.m199898@gmail.com*

## Aim

This study aimed to compare the water sorption of a nanohybrid composite resin and two 3D-printed resin Material over varying storage periods.

## Material and Methods

A total of 60 cylindrical specimens (8.0 mm diameter and 2.0 mm thickness) were prepared and divided into three groups (n=20) based on the Material used: a nanohybrid composite (Kuraray Clearfil Majesty Esthetic, NC, Japan) and two 3D-printed resin Material (Saremco Crowntec, SC, Switzerland; VarseoSmile Crown Plus, VS, Germany). NC specimens were fabricated using stainless steel molds, while SC and VS specimens were prepared using a Phrozen Sonic Mini 8K SLA 3D printer (Phrozen Technology, Taiwan). Post-processing for SC and VS included immersion in isopropyl alcohol for 5 minutes and UV curing at 405 nm for 10 minutes. All specimens were stored in a desiccator until a constant mass was achieved and then immersed in distilled water. Water sorption (WS) was evaluated according to ISO 4049:2009 standards at 1, 7, 30, and 90 days. Statistical analysis was performed using one-way ANOVA and Tukey’s post-hoc test at a significance level of p<0.05.

## Results

The nanohybrid composite (NC) exhibited significantly higher water sorption values at all time points compared to the 3D-printed groups (SC and VS) (p<0.05). Between the 3D-printed resins, SC showed significantly higher water sorption than VS at 1 and 7 days (p<0.05) however, no significant difference was observed at 30 and 90 days (p>0.05). All Material demonstrated a rapid increase in water sorption in the first week, followed by a slower rate of absorption as storage time progressed.

## Conclusions

The nanohybrid composite (NC) exhibited greater water sorption compared to the 3D-printed resin materials. These findings suggest that 3D-printed resin materials, may be a suitable alternative for long-term clinical restorations.

# Abstract: 05. The effect of surface treatments on the bond strength of different esthetic restorative materials

**Authors:** A. Sahin, S. Baglar

**Affiliations:** *Department of Restorative Dentistry, Faculty of Dentistry, Ordu University, Altınordu/Ordu, Turkey. aysenur.sahin.700@gmail.com*

## Aim

The aim of this study was to evaluate the effects of surface treatments (sandblasting, hydrofluoric acid, Nd:YAG laser) on bond strength of different CAD/CAM and a nanohybrid composite restorative. The null hypothesis was, surface treatments and adhesive system variations have no effect on bond strength of restorative Material to enamel.

## Material and Methods

168 caries-free enamel samples were used. CAD/CAM Material and enamel surfaces were standardized by using Sof-Lex discs (3M ESPE, USA). CAD/CAM groups [Lava Ultimate (3M ESPE, USA) and Vita Enamic (Vita Zahnfabrik, Germany)] were divided into four subgroups according to surface treatments: sandblasting (Al₂O₃), hydrofluoric acid (9.5%HF), Nd:YAG laser (20Hz) and control. CAD/CAM specimens were bonded to enamel by using resin cement [RelyX U200 (3M ESPE, USA)]. In composite group [Filtek Supreme (3M ESPE, USA)], Material were divided into two subgroups of total-etch and self-etch. Each subgroup was further divided into three groups of Nd:YAG laser (20Hz), Nd:YAG laser (10Hz), and control. Surface treatments were applied to tooth enamel and composites were bonded via silicone mold. Bond strength was measured by universal testing machine, and surfaces analyzed by using SEM. One-way ANOVA and Post-hoc Tukey’s tests were used for statistical analysis.

## Results

The sandblasting group of Vita Enamic exhibited the highest bond strength (29.06 ± 2.8 MPa). Laser treatments improved bond strength for both Lava Ultimate and Vita Enamic compared to the control. In the composite group, total-etch performed better than self-etch across all treatments. The highest SBS was observed in total-etch with 10Hz laser (22.85 ± 1.62 MPa), while the lowest was in self-etch with 20Hz laser (11.78 ± 1.29 MPa, p<0.05).

## Conclusions

Nd:YAG laser surface treatment is adequate in comparison to other conventional methods.

# Abstract: 06. Do fast food sauces affect the surface properties and colour stainability of CAD/CAM and resin-based materials?

**Authors:** H. Ozyilmaz, A. Sahin, S. Baglar

**Affiliations:** *Department of Restorative Dentistry, Faculty of Dentistry, Ordu University, Altınordu/Ordu, Turkey. hkucukali61@gmail.com*

## Aim

This study aims to investigate the color stability and surface roughness of different aesthetic restorative Material exposed to various fast food sauces. First null hypothesis posits that fast food sauces will not cause any changes in the color of restorative Material and that there will be no significant differences in color change among the Material used. The second null hypothesis is that there would be no differences in surface roughness among the tested restorative materials.

## Material and Methods

Samples were prepared using resin nanoceramic blocks (Lava Ultimate, 3M ESPE, St. Paul, MN, USA (LU)), lithium disilicate glass-ceramic (IPS e.max CAD Opal, Ivoclar Vivadent, Liechtenstein (IC)), nanoceramic composite (Ceram.X SphereTEC One, Dentsply, Sirona, Konstanz, Germany (CX)), and hybrid resin (Palfique Estelite Paste, Tokuyama Corp., Japan (PE)). The samples were immersed in different fast-food sauces, ketchup, BBQ, curry, mustard, for 1, 7, and 14 days. Surface roughness was measured using a profilometer, and color changes were analyzed using a spectrophotometer. In this study, 10 specimens per group were used, resulting in a total sample size of 160.

## Results

The most significant color change was observed on 14th day in the ketchup-exposed Tokuyama group samples (6.58 ± 3.33 ΔE). IPS e.max samples maintained color stability regardless of immersion time or solution. After polishing at the end of the experiment, all samples returned to clinically acceptable ΔE color values of <3.3. The highest surface roughness increase was observed in Tokuyama-Curry samples at the end of day 1.

## Conclusions

Fast food sauces may have a potential to cause superficial discoloration on restorative materials. Besides this, polishing has managed to recovery of color.

# Abstract: 07. The effect of toothbrushing via different toothpastes in removing the discoloration of 3D-printed laminate veneer restorations

**Authors:** S. Kucuk, C. Atalayın Ozkaya, F.S. Barhan, C. Peskersoy, H. Tezel

**Affiliations:** *School of Dentistry, Ege University, İzmir, Turkey. sulekck98@gmail.com*

## Aim

The aim of this in vitro study is to investigate the effectiveness of toothbrushing applied with different toothpastes for removing the discoloration of 3D-printed laminate veneer restorations. The first null hypothesis is that the prolonged immersion in coffee will not affect the color of the 3D resin samples. The second hypothesis is that the non-invasive procedure such as toothbrushing will have no effect on the color restitution of 3D resins immersed in coffee.

## Material and Methods

Two different 3D printing resins (Alias Temporary 3D Printing Resin-Dokuz Kimya and VarseoSmile Crown Plus-Bego) were tested. A 3D-printer (Varseo XS-Bego) was used to produce a total of 80 laminates, 40 of each resin type. After the baseline color measurement by spectrophotometer (Vita Easyshade Advance 5.0-Vita Zahnfabrik), the specimens were immersed in distiled water and coffee (Nescafe-Nestle) for 15 days and the color measurement was repeated. Then, the specimens were subjected to toothbrushing simulation for 3 minutes twice a day for 15 days via an electric toothbrush (Vitality-Oral B) with different toothpastes as Colgate Sensation White-Palmolive, Ipana Pro-Expert-Ipana and Bioniq-Dr. Wolff. The color measurement was repeated and the color changes after discoloration (ΔE1) and toothbrushing simulation (ΔE2) were calculated. Statistical analyses were conducted using SPSS version 27.0 (p<0.05).

## Results

The differences between ΔE1 values of all resin groups were significant (p˂0.05). After toothbrushing simulation, the VarseoSmile Crown Plus resins stored in coffee showed significant color restitution (ΔE2bioniq=11.70, ΔE2colgate=11.79, ΔE2ipana=11.79), but Alias Temporary 3D printing resin showed significant color restitution (ΔE2=18.69) only via Ipana Pro-expert toothpaste (p<0.05).

## Conclusions

The beverages such as coffee seems to negatively affect the color of the 3D printing resins. The regular toothbrushing and the choice of a proper toothpaste seems to be the noteworthy points to obtain the best outcomes for color restitution in 3D-printed restorations.

# Abstract: 08. Influence of tobacco heating system on color stability and surface roughness of contemporary restorative materials

**Authors:** A. Uslu Tekce

**Affiliations:** *Department of Restorative Dentistry, School of Dentistry, Hacettepe University, Ankara, Turkey. aybukeuslu91@gmail.com*

## Aim

This study aimed to evaluate the effect of the Tobacco Heating System (THS) on the color stability and surface roughness of contemporary resin composite materials. The null hypothesis was that one-week THS exposure would not affect the color or surface roughness of the tested restorative materials.

## Material and Methods

A total of 48 samples, 12 from each group (Filtek Z550 [3M], Omnichroma [Tokuyama], G-aenial Universal Injectable [GC], and Aura-Bulk Fill [SDI]), were prepared using molds (2mm thickness, 5mm diameter). Following finishing and polishing (Kerr OptiDisc, Kerr; Polishing paste, SDI) baseline color (Easy Shade-VITA) and surface roughness (Perthometer M2, Mahr) measurements were recorded. Specimens were exposed to a custom-made THS system for one week (400 puffs/day) under controlled conditions (37°C, 60% humidity). Post-exposure color and surface roughness measurements were performed. Color differences (ΔE00) were calculated using the CIEDE2000 formula. Statistical analyses were conducted using Kruskal-Wallis and Wilcoxon tests with Bonferroni correction (α = 0.05).

## Results

THS exposure did not lead to a statistically significant change in the surface roughness of any tested material (p>0.05). However, it caused significant color changes in all tested materials, with none maintaining their color stability. Moreover, significant differences were observed among the tested Material (p<0.05), except between Omnichroma and G-aenial Universal Injectable; Filtek and Aura Bulk Fill (p>0.05). Omnichroma showed the highest color change, while Filtek had the least.

## Conclusions

One-week THS exposure significantly affected the color stability of restorative materials, with Omnichroma showing the highest color change and Filtek the least. Surface roughness was not significantly altered following THS exposure.

# Abstract: 09. In vitro biocompatibility of titanium-dioxide nanotubes reinforced glass-ionomers

**Authors:** B. Oglakci Ozkoc, V.B. Yenigün, Z.C. Özduman, B.O. Kucukyildirim, A. Kocyigit, E. Eliguzeloglu Dalkilic

**Affiliations:** *Department of Restorative Dentistry, Faculty of Dentistry, Bezmialem Vakif University, İstanbul, Turkey. burcu923@hotmail.com*

## Aim

This in vitro study evaluated the biocompatibility properties of titanium-dioxide (TiO2) reinforced glass-ionomer cements (GICs).

## Material and Methods

Conventional GICs (Ionofil Molar, Voco GmbH) were reinforced with varying amounts (0.1%, 0.5%, 1%, 2%) of non-functionalized and functionalized TiO2 nanotubes(f-TiO2). Specimens were divided into nine groups. IM0:no reinforced (control), IM1:0.1%TiO2, IM2:0.5%TiO2, IM3:1%TiO2, IM4:2%TiO2, IM1f:0.1%f-TiO2, IM2f:0.5%f-TiO2, IM3f:1%f-TiO2, IM4f:2%f-TiO2. Nanotubes were characterized with X-ray diffraction (XRD), FTIR spectroscopy, specific surface area (BET), transmission electron microscope (TEM) and scanning electron microscope (SEM). Disc-shaped GIC specimens(5×2mm) were fabricated and immersed in culture medium at 37ºC for 24 h. Then, extracted medium were applied on human normal fibroblast cells (CCD-1079Sk) and incubated for 24h and 48h. Cytotoxicity was measured using ATP cell viability test. Reactive Oxygen Species (ROS) production was quantified by 2′–7′dichlorodihydrofluorescein diacetate (H2DCFDA) method. Genotoxicity was assessed using Alkaline Single-Cell Electrophoresis Assay (Comet), while apoptosis was evaluated through Acridine Orange/Ethidium Bromide double staining. Experimental procedures were repeated three times. Two-way ANOVA and Bonferroni tests were used for statistical analyses (p<0.05).

## Results

At 24h incubation, when comparing to Group IM0(control) (96.51±2.88), for non-fuctionalized groups, Group IM3(89.36±5.71) exhibited significantly higher cytotoxicity (p<0.05) while all functionalized groups (98.28±4.29/99.93±5.06/94.95±4.77/91.78±4.43) showed similar cytotoxicity, respectively(p>0.05). At 48h incubation, for functionalized groups, Group IM4f (84.96±4.61) showed significantly higher cytotoxicity (p<0.05) whereas there were no significant differences in cytotoxicity between Group IM0 (91.24±2.07) and all non-functionalized groups (90.94±3.18/88.71±3.04/87.34±1.80/91.86±2.91) (p>0.05). At 24h and 48h, Group IM0 showed similar genotoxicity (3.557±4.309/2.969±2.675) and ROS activity (1.051±0.079/1.032±0.069) to all reinforced groups(p>0.05). At 24h, for functionalized groups, Group IM3f (6.421±4.234) exhibited significantly higher apoptosis than Group IM0 (1.811±4.081) (p<0.05), all non-functionalized groups (4.607±5.225/4.305±2.376/8.045±6.614/4.304±3.089) showed similar apoptosis (p>0.05). At 48h, no significant differences in apoptosis were determined between Group IM0 (8.94±7.314) and all reinforced groups (p>0.05).

## Conclusions

Certain high concentrations of TiO2 nanotube additives might increase the apoptosis and cytotoxicity of conventional GIC, while reinforcement had no negative effect on ROS activity and genotoxicity. Funding: Health Institute of Turkey (TUSEB)-Innovative Drug Development Strategic R-D Project Support 2020-YI-02 (Project Number:6689).

# Abstract: 10. Fracture strength of high-viscosity bulk-fill composites: a comparison of MOD cavities and ISO standard bars

**Authors:** E.A. Kahraman Karsigil, B. Ozturk

**Affiliations:** *Department of Restorative Dentistry, Faculty of Dentistry, Ankara University, Ankara, Turkey. easlihankahraman@gmail.com*

## Aim

This study aimed to evaluate the fracture strength of high-flow bulk-fill composites prepared in two different geometries: MOD cavity-shaped and ISO standard bar-shaped molds.

## Material and Methods

Eighty samples were divided into two groups: MOD cavity-shaped (n=40) and ISO standard bar-shaped molds (n=40). Each group contained four composite subgroups (n=10): GC Posterior (control), Clearfil Majesty High Flow Bulk Fill, Tetric N-Ceram High Flow Bulk Fill, and Tokuyama Estelite High Flow Bulk Fill. MOD cavity samples were incrementally filled and light-cured in two layers, while bar-shaped samples were fabricated using 3D-printed molds and light-cured in three steps. Fracture strength was tested using a universal testing machine (three-point bending setup, 1 mm/min). Data were analyzed with one-way ANOVA and Tukey’s post hoc test (p<0.05).

## Results

In the MOD cavity group, Clearfil Majesty (1133.10 ± 171.12 N) and Tetric N-Ceram (1198.33 ± 188.83 N) exhibited significantly higher fracture strength than GC Posterior (871.92 ± 227.67 N, p<0.05). In the ISO standard bar group, Tokuyama Estelite (85.84 ± 23.43 N) and Clearfil Majesty (68.58 ± 28.44 N) showed superior strength compared to GC Posterior (54.19 ± 11.57 N, p<0.05).

## Conclusions

High-flow bulk-fill composites exhibited superior fracture strength compared to conventional composites. The MOD cavity configuration enhanced fracture performance, emphasizing its clinical relevance. These findings support the use of high-flow bulk-fill composites in restorative dentistry for improved mechanical durability.

# Abstract: 11. Investigation of pH variations following polymerization of an MDP-based all-in-one adhesive on composite surfaces

**Authors:** E. Dikici, Y.H. Bagis

**Affiliations:** *Department of Restorative Dentistry, Faculty of Dentistry, Ankara University, Ankara, Turkey. ekindikici@hotmail.com*

## Aim

This study aimed to evaluate the pH behavior of an MDP-containing adhesive on a pre-polymerized composite resin surface and in a humid environment without polymerization. The hypothesis was that the adhesive would retain its acidic effect after polymerization, while humid storage might stabilize its pH.

## Material and Methods

The adhesive’s (G-Premio Bond, GC, Japan) pH was tested under two conditions. On a 1 mm-thick pre-polymerized composite resin layer (G-ænial Posterior, GC, Japan), pH was measured using a digital pH meter (LAQUAtwin pH-11, Horiba, Japan) before and after polymerization for 20 seconds with a light-curing device. In a humid environment (dark, 100% humidity, 37°C), pH was measured before and after 5 minutes of incubation. Fifteen specimens were tested for each condition. Data were analyzed using the Wilcoxon signed-rank test (SPSS 22, p<0.05).

## Results

The adhesive’s pH significantly decreased after polymerization on the composite surface (p<0.05), yet it remained acidic. In the humid environment, no significant pH change was observed after incubation (p>0.05).

## Conclusions

This study suggests that the acidic behavior of an MDP-containing adhesive may persist after polymerization, which could potentially contribute to bonding challenges during composite repair and might influence dentin demineralization, potentially leading to pulpal reactions. The observation of a stable pH in a humid environment prior to polymerization indicates that adhesives without solvents like ethanol might be applied after a brief waiting period without affecting their performance. Nevertheless, the potential for persistent acidity post-polymerization warrants caution in clinical applications, particularly when applying a second adhesive layer, as it could influence monomer bonding. Further studies are needed to validate these findings and explore their clinical implications.

# Abstract: 12. The influence of bleaching tray design on the occurrence of gingival irritation: A randomized clinical trial

**Authors:** P. Pereira Lores, T. Gancedo Gancedo, V. Alonso de la Peña, I. Varela Aneiros, B. Martín Biedma, P. Castelo Baz

**Affiliations:** *Department of Endodontics and Restorative Dentistry, School of Medicine and Dentistry, University of Santiago de Compostela, Santiago de Compostela, A Coruña, Spain. patriciapereiralores@gmail.com*

## Aim

This randomized clinical trial aimed to evaluate the potential relationship between the design of bleaching trays and the incidence of gingival irritation during at-home teeth bleaching treatments. Additionally, it aims to compare differences in dental sensitivity and the effectiveness of the bleaching treatments.

## Material and Methods

It was registered on ClinicalTrials.gov. (NCT06371664) and included 72 participants randomly allocated into two groups: one using 3 mm bleaching trays and other using 1 mm bleaching trays. Participants underwent a nightly bleaching regimen (6 hours) over 3 weeks with 16% carbamide peroxide gel. Gingival irritation was assessed daily by participants and during scheduled visits by clinicians. Tooth sensitivity was recorded daily using a 5-point numerical scale, while color was measured with a dental spectrophotometer. Statistical analyses included the Pearson chi-squared test, Fisher’s exact text, and the Student’s t-test, with a significance threshold of α=.05.

## Results

Subjective assessments showed gingival irritation risk of 66.7% for the 3 mm tray group and 47.2% for the 1 mm tray group, a difference that was not statistically significant (p>.05). Objective clinical evaluations revealed significantly higher gingival irritation in the 3 mm tray group (88.9%) compared to the 1 mm tray group (63.9%) (p=.01(95% CI 1.06 to 1.83). Although tray design did not significantly influence the risk of tooth sensitivity (p>.05), sensitivity intensity was notably higher in the 3 mm tray group (p<.001). No significant differences in tooth color changes were observed between the groups (p>.05).

## Conclusions

Extended bleaching trays increase both gingival irritation and tooth sensitivity intensity. To minimize side effects, a 1 mm tray design in recommended.

# Abstract: 13. In vitro evaluation of the effect of plaque disclosing agents on the color stability of different restorative materials

**Authors:** I. Menekse, Ü.B. Efes

**Affiliations:** *Department of Restorative Dentistry, Faculty of Dentistry, Istanbul University, Istanbul, Turkey. incimenekse99@gmail.com*

## Aim

Plaque disclosing agents, which stain dental plaque on tooth surfaces, are commonly used by dentists to assess and educate patients about their oral hygiene. The aim of this study was to evaluate the effect of two plaque disclosing agents on the color parameters of four different restorative materials.

## Material and Methods

A total of 80 disc-shaped specimens, 8 mm in diameter and 2 mm in thickness, (n=20) were prepared from three different composite resins (Filtek Z250; Charisma Diamond; Estelite Universal Flow Medium) and a giomer (Beautifil II). Baseline color measurements were made by VITA Easyshade Compact after the specimens were numbered and kept in distilled water at 37°C for 24 hours. The plaque disclosing solution Mira-2-Ton was applied to the surface of 10 specimens of each group and Propind Plaque Indicator Gel was applied to the surface of the other 10 specimens. After removing the agents from surface by washing, color measurements were made for the second time. Color changes in the specimens were calculated using the CIEDE2000 formula. One-way Analysis of Variance, Kruskal Wallis and Mann Whitney-U test were used for statistical analysis (p<0.05).

## Results

After the application of Mira-2-Ton solution, clinically unacceptable color changes were observed in all groups. These Delta E00 values were determined as; Filtek Z250:2,98 ± 1,13, Charisma Diamond:7,18 ± 2,65, Estelite Universal Flow Medium:3,26 ± 1,87, Beautifil II:5,36 ± 3,03. All the groups stained with Propind gel did not show clinically relevant discoloration with no statistically significant difference between the Delta E00 values (p>0.05). The color change in specimens applied with Mira-2-Ton solution was found to be statistically significantly higher compared to those applied with Propind gel (p<0,05).

## Conclusions

Considering that plaque disclosing agents may cause color changes in restorations, further studies using a wide variety of this agents and under clinical conditions are needed.

# Abstract: 14. Effect of different finishing-polishing techniques on microhardness-surface roughness properties of various flowable composites

**Authors:** B. Erol, I.C. Sahin, I.N. Caliskan, G. Ozan, H. Sar Sancakli

**Affiliations:** *Department of Restorative Dentistry, Institute of Graduate Studies in Health Sciences, Istanbul University, Istanbul, Turkey. beyzaerol@ogr.iu.edu.tr*

## Aim

Flowable composites have low viscosity hence, it can be easily applied into cavities, reducing the need for manual manipulation, which can lead to quicker and more adaptive restorations. However, one of the challenges of flowable composites is maintaining their mechanical and physical surface properties, which can be influenced by polishing techniques. The aim of this in vitro study is to evaluate surface roughness and microhardness of different flowable composites with different polishing techniques. The null hypothesis is different polishing techniques wouldn’t have any significant difference to various flowable composites.

## Material and Methods

A total of 84 discs (4mm*8mm) from a bulkfill flowable composite (Tetric N-Powerflow 2-TP), a highly filled flowable composite (Filtek Supreme Flow-FS), a single-shade flowable composite (Vittra APS Unique Flow-VU) and an injectable composite (G-aenial Universal Injectable-GU) were obtained by using a custom-made stainless-steel mold. LED light was applied for 20 seconds on each surface. The bottom surfaces of all discs were determined and numbered. The samples were divided into 3 subgroups according to the polishing system applied(n=7): G1) Aluminum oxide embedded polishing discs, G2) Diamond embedded polishing discs, G3) Rubber set (coarse and soft). Hardness and surface roughness values were measured. Data were analyzed using ANOVA. The significance level was set at p<0.05.

## Results

The roughness values of composites show differences (p<0.001). Using different polishing techniques affected roughness values (p<0.001). Regardless of composite material, G3 subgroups gave the highest roughness value. While TP-G3 subgroup has the highest roughness (median=1,68), FS-G2 subgroup has the lowest roughness (median=0,24). No significant difference was observed between all composites’ G1 and G2 subgroup roughness values(p>0.05). Using various polishing techniques affected microhardness values (p<0.001). While TP-G1 subgroup has the highest microhardness (median=85,15), VU-G1 subgroup has the lowest microhardness (median=49).

## Conclusions

Polishing techniques affect physical and mechanical properties of composite surfaces. Therefore, clinicians should spare adequate time for finishing-polishing procedures.

# Abstract: 15. Effect of breakfast alternatives on the surface microhardness of resin composites

**Authors:** K. Karaosmanoglu, A. Mujdeci

**Affiliations:** *Department of Restorative Dentistry, Faculty of Dentistry, Ankara University, Ankara, Turkey. dtkubrayazici@gmail.com*

## Aim

The aim is to assess the influence of breakfast alternatives on the microhardness of microhybrid and nanohybrid resin composite materials.

## Material and Methods

Forty cylindrical specimens of a microhybrid (G-aenial Posterior) and a nanohybrid resin composite (Estelite Sigma Quick) were prepared (6x2 mm) and stored in distilled water (370C, 7 days). After baseline Vickers microhardness measurements of all specimens were taken (100 g load, 20 seconds dwell time), they were randomly divided into 4 subgroups (n=10) and immersed in one of the breakfast alternatives: nutritional powder drink milk, overnight oats, overnight oats with added nutritional powder drink or control (distilled water). The immersion protocol for test groups was performed as a cycling (15 times in each breakfast alternative for 3 seconds, alternating immersion in distilled water for the same time) once a day for 30 days. They were stored in distilled water (24 hours, 37°C) until the next daily cycling. Control group immersed in distilled water through the study. After 30 days, all the specimens' final Vickers microhardness measurement was performed the same as for the baseline measurement. Data were analyzed with 2-way ANOVA and Tukey HSD tests (α=.05).

## Results

Resin composites, breakfast alternatives and their interactions were significant (p=0.000). Overnight oats (9.76±0.68) and overnight oats with added nutritional powder drink (10.31±0.61) caused the most microhardness decreases in nanohybrid composite. In contrast, overnight oats (10.89±0.54) and nutritional powder drink milk (11.63±0.85) were the most softening effect in microhybrid composite. Microhybrid composite had more softening potential than nanohybrid composite in all breakfast alternatives and the control except for overnight oats with added nutritional powder drink (p<0.05).

## Conclusions

The breakfast alternatives and the control negatively affected the microhardness of both resin composites, depending on the resin composites’ structure and the ingredients of the breakfast alternatives. With one exception, the microhybrid composite showed a higher microhardness decrease than the nanohybrid composite.

# Abstract: 16. Effect of collagen crosslinkers used in dentin pretreatment on immediate dentin microtensile bond strength

**Authors:** B. Tekin, H.S. Sancakli, G. Ozan

**Affiliations:** *Department of Restorative Dentistry, Faculty of Dentistry, Istanbul University, Istanbul, Turkey tekinbusr@yandex.com*

## Aim

Theobromine and Theaflavin are consisted of phenolic compounds that have the potential of modifiying the double carbon bonds to collagen. Thus, these compounds may improve the adhesive bond strength to dentin and be used among the adhesive steps. To evaluate the effect of collagen crosslinking agents used as primers for dentin biomodification on dentin microtensile bond strength in vitro.

## Material and Methods

A total of 15 caries free human wisdom teeth used in the study. Intact dentin surfaces obtained by cutting 4mm below the occlusal surface with a diamond blade horizontally. Then all of the surfaces etched with 37% orthophosphoric acid for 15s. After washing for 15s, all of the surfaces were air-dried gently. Experimental groups were consisted of two crosslinkers of each concentrated 2% Theabromin (TB) and Theaflavin (TF). Afterward, each crosslinkers applied to intact dentin surfaces for 15s and air-dried for 5 sec. Then primer and bonding of optibond FL was applied. In the control group, only Optibond FL adhesive applied to dentin surfaces after etching for 15s. Build-ups were performed with a microhybrid composite. Dentin beams with a 1x1mm cross-section perpendicular to the adhesive dentin bonding surfaces were obtained in each group (n=50). Then, microtensile bond strength values were performed and failure modes were also recorded. The data were evaluated by Chisquare and Bonferroni test at p<0.05.

## Results

According to the data, the use of cross-linkers significantly changed dentin immediate microtensile strength (p>0.001). The highest MPa value was in the TB group (23.4MP). The lowest MPa value was in the TF group (13.45MPa). There were no significant differences among the control and TB groups (p>0.05). The TF group showed more adhesive failure, while the TB group showed both adhesive and cohesive failures.

## Conclusions

Immediate dentin microtensile bond strength has improved by applying ‘Teobromin’ as primer. In order to introduce such dentin surface biomodifying Material into clinical practice, long-term studies that are examining both of the adhesive properties and bonding interface are needed.

# Abstract: 17. Effect of battery level of a light curing unit on the microshear bond strength of cad/cam Material to zirconia

**Authors:** I.C. Sahin, Z.H. Keles, M. Berkman, H. Sar Sancakli, G. Ozan

**Affiliations:** *Department of Restorative Dentistry, Institute of Graduate Studies in Health Sciences, Istanbul University, Istanbul, Turkey. irmakcesminaz.sahin@ogr.iu.edu.tr*

## Aim

The rising usage of zirconium restorations underscores the need to optimize material combinations, as veneering feldspathic ceramic onto zirconia-based restorations merges the durability of zirconia with the enhanced aesthetic qualities of veneering porcelain. This study aimed to evaluate the impact of the battery level of a light curing unit on the microshear bond strength between feldspathic CAD/CAM blocks and sintered zirconia.

## Material and Methods

Sintered zirconia blocks in 4×4×2 mm were subjected to airborne-particle-abraded and divided into two groups according to 100% (Group A) and 10% (Group B) battery levels of an LED light curing unit. Silane primer and a universal adhesive system were applied to the prepared zirconia block surfaces. Feldspathic ceramic blocks (n=7) prepared in the same dimensions (4×4×2 mm) were etched with 10% hydrofluoric acid for 60 seconds, then silane primer and universal adhesive system were applied. Ceramic blocks were cemented to zirconia blocks using dual-curing adhesive resin cement and polymerized for 20 seconds with an LED curing unit with 100% battery level in Group A and 10% battery level in Group B. Blocks kept in distilled water at 37°C for 24 hours were sectioned into sticks and subjected to μTBS tests. Statistical analysis was performed using one-way ANOVA, with a significance set at p<0.05.

## Results

Main SBS values were 45 MPa for %100 battery level and 36 MPa for %10 battery level subgroup. A significant decrease in bond strength (p<0.05) was observed with lower LED battery levels, while DC values remained unaffected (p>0.05).

## Conclusions

Reduced LED battery levels significantly impact the microshear bond strength between feldspathic ceramics and zirconia. To achieve optimal bond strength, maintaining a fully charged battery during adhesive cementation is crucial. Further studies could be designed for evaluating the adhesive strength of different adhesive cements or blocks.

# Abstract: 18. Clinical assessment of the masking effect of resin infiltration treatment on molar incisor hypomineralized enamel surfaces

**Authors:** M.D. Casaña Ruiz, M.A. Velló Ribes, M. Catalá Pizarro

**Affiliations:** *University of Valencia, Valencia, Spain. maria.d.casana@uv.es *

## Aim

Enamel defects in molar-incisor hypomineralization (MIH) stem from a combination of environmental, systemic, and genetic factors, indicating a multifactorial etiology. These defects pose a significant aesthetic challenge, particularly in anterior teeth, creating not only a visible impact but also substantial emotional implications for patients due to their high visibility The objective of this study is to assess the effect of infiltration resins on MIH lesions in terms of color, size and sensitivity improvement.

## Material and Methods

A total of 109 teeth diagnosed with MIH defects were treated with Icon® (DMG, Hamburg, Germany) infiltration resins, and 101 were monitored over six months. The evaluation included photographic follow-up, assessment of size and color, and measurement of sensitivity improvement after treatment.

## Results

The values obtained from the spectrophotometer before treatment suggest that the hypomineralizations exhibited a slight reddish tendency (mean a* = 2.12) and were notably yellowish (mean b* = 23.20), with a significant color difference compared to the baseline color of the tooth, ΔE (Delta E) = 8.62. The mean value of L* = 69.81 indicates that the studied surface had medium-high brightness (0 represents absolute black, and 100 represents absolute white). The reduction in lesion size yielded significant results, with an average decrease of 4.5 percentage points. Additionally, a positive trend was observed in terms of reduced sensitivity. Notably, 36.6% of patients showed an improvement of 1 to 2 points on the SCASS scale.

## Conclusions

Following the application of resin infiltration, a color variation is observed due to the significant shift in Delta E in MIH opacities, along with a marked reduction in lesion size and a significant improvement in sensitivity. Thus, treatment with Icon infiltration resin should be considered as an option for managing anterior teeth affected by molar incisor hypomineralization in pediatric patients.

# Abstract: 19. Does an additional curing effects the mechanical/physical properties of nanohybrid composites used in semi-direct technique

**Authors:** I.N. Caliskan, E. Sorrentino, M. Turk, G. Ozan, C. D'Arcangelo, H. Sar Sancakli

**Affiliations:** *Department of Restorative Dentistry, Faculty of Dentistry, Istanbul University, Istanbul, Turkey. iremnaz.caliskan@ogr.iu.edu.tr*

## Aim

The present study aimed to evaluate mechanical and physical properties of two different artificially aged nanohybrid composites indicated for semi-direct restorations regarding different polymerization methods. The null hypothesis of this study is that additional polymerization wouldn’t affect the mechanical and physical properties of aged composites.

## Material and Methods

A total of 40 discs (2mm*8mm) were prepared with a biocompatible nanohybrid composite (Micerium Enamel Biofunction) and a highly-filled composite (Voco GrandioSo) and polymerised with a LED light-curing unit (Elipar Deep Cure-S) for 40s on each side. The samples were then divided into two groups (n=10): a) Light-cured and b) Light-cured Tescera ATL oven. First, all samples were then soaked in artificial saliva. Then, all samples were subjected to thermal-cycle for 5000 cycles. Surface roughness and microhardness measurements were obtained. Homogenity and normality of measurements were confirmed by using Shapiro-Wilk test with p<0.05 significance with Tukey’s test. One sample from each group was evaluated under SEM.

## Results

Higher filler composition and additional curing showed most influential factors to enhance mechanical properties of composites, while surface roughness was primarily affected by artificial aging methods (p<0.001). Obtained data also indicated that both group (p=0.007) and subgroup (p=0.006) factors significantly affected surface roughness. Time factor was less impactful on mechanical properties (p=0.03). Additionally cured Voco samples has the highest microhardness (Mean=115.23) and surface roughness (Ra=0.39).

## Conclusions

Within the limitation of the study, it can be understood that surface roughness can be affected mostly by artificial aging protocols while additional curing and composite resin content have a significant impact on mechanical properties. Further studies are needed to assess the clinical relevance of other variables to heating process.

# Abstract: 20. Fluorescence of different milled composites under ultraviolet light: an in vitro study

**Authors:** D.F. Saa Valencia

**Affiliations:** *Catholic University of Valencia, Valencia, Spain. diegofsaa@gmail.com*

## Introduction

This theoretical framework explores the fundamentals of fluorescence in milled composite resins, their chemical composition, and the techniques used to evaluate this optical phenomenon, based on this in vitro study and previous research.

## Objectives

1. Determine the degree of visible fluorescence in milled composites. 2. Analyze their similarity to natural teeth, according to standardized fluorescence.

## Material and Methods

1. Type of study: Descriptive, observational, cross-sectional. 2. Three samples of each nanohybrid composite resin block were obtained in shades A1, A2, and A3, with dimensions of 3 mm × 3 mm and thicknesses of 0.5, 1.0, and 1.5 mm for the nanohybrid composite resin blocks. The blocks were cut using a diamond disc and carbide bur in a handpiece and polished with an Ultraden disc. UV light was applied, and measurements were taken for each sample using a UV spectrophotometer, obtaining the a*, b*, and L* values for each sample. The formula ΔE = √((L₂−L₁)² + (a₂−a₁)² + (b₂−b₁)²) was used to compare the samples with the range of natural teeth according to previous research. 3. A two-way ANOVA was performed to evaluate the effect of material, thickness, and their interaction on the L, a, b coordinates.

## Results

• VOCO Grandio Blocs showed fluorescence values (b*: 9.4–18.4) closer to those of natural teeth (b* = 15) than Coltène Brillant Crios (b*: 12.1–18.9). • Luminosity (L)* increased with thickness (0.5 → 1.5 mm) in both materials, but Coltène exceeded L* = 85 in all cases.

## Conclusions

1. VOCO Grandio Blocs exhibited fluorescence values (b*: 9.4–18.4) closer to the natural tooth range (b* = 12–18) than Coltene Brilliant Crios (b*: 12.1–18.9), highlighting its ability to replicate natural aesthetics under ultraviolet light. 2. Luminosity (L)* increased with thickness (0.5 to 1.5 mm) in both materials. VOCO approached the L* = 85 standard at greater thicknesses (up to 85.9), while Coltene consistently exceeded it (87.1–92.7), suggesting lower naturalness in terms of brightness.

# Abstract: 21. Facial aesthetic analysis using neural networks and computer vision in comparison with Digital Smile Design (DSD)

**Authors:** N. Rodriguez

**Affiliations:** *Catholic University of Valencia, Valencia, Spain. nicolecamilard1@gmail.com*

## Introduction

Despite the widespread adoption of digital smile design (DSD) in aesthetic dental treatment planning, it remains limited to static smile evaluations. In this study, we present an innovative methodology based on computer vision techniques and neural networks for functional and facial aesthetic assessment. Our proposed approach also incorporates specific measurements, including horizontal and vertical symmetry, lip-dental alignment, and upper/lower facial aspects, which complement and surpass the scope of traditional digital smile design. These analyses enable improved evaluation of aesthetic mockups in smile design and facilitate personalized treatment planning.

## Material and Methods

Static Smile Analysis Using Mediapipe Face Mesh, key facial landmarks are detected in static images to calculate: • Horizontal and Vertical Symmetry: The difference in distances from facial points to the midline of the face is measured. • Lip and Dental Alignment: The width of the lips and the vertical alignment of the lips with the teeth are calculated. • Facial Proportions: The ratio between the upper and lower areas of the face relative to the midline is evaluated.

## Conclusions

The proposed model represents an innovation in smile analysis and design by integrating functional metrics, facial proportions, and automated prediction based on neural networks. It offers much more than DSD as a tool for aesthetic evaluation and treatment planning in dentistry.

# Abstract: 01. Atherosclerosis and apical periodontitis: systematic review with meta-analysis

**Authors:** M. León López, J. Martín González, B. Sánchez Domínguez, D. Cabanillas Balsera, J.J. Saúco Márquez, J.J. Segura Egea

**Affiliations:** *Endodontics Group, Department of Stomatology, Faculty of Dentistry, University of Seville, and Andalusian Health Service, Seville, Spain. maria.leon.lopez.98@gmail.com*

## Aim

Atherosclerosis is a chronic condition of the arteries, characterized by the thickening and hardening of their walls due to the formation of atherosclerotic plaques. Low-grade inflammation is implicated in pathogeny of atherosclerosis. Chronic apical periodontitis (CAP), the chronic inflammation around the root apex of infected teeth, is associated with a low-grade inflammatory state, so a connection between atherosclerosis and CAP have been suggested. The aim of this study was to conduct a systematic review with meta-analysis to answer the PICO question: In adult patients, does presence or absence of atherosclerosis affect the prevalence of CAP?

## Material and Methods

PRISMA guidelines were followed to carry out this systematic review. Inclusion criteria selected the studies presenting data on the prevalence of CAP in patients diagnosed with atherosclerosis and control patients. The statistical analysis was carried out using Open Meta Analyst software. Study characteristics and risk ratios with 95% confidence intervals (CIs) were extracted. Random-effects meta-analyses were performed. Risk of bias was assessed using the Newcastle-Ottawa scale, adapted for cross-sectional studies. To estimate variance and heterogeneity between trials, the Higgins I2 test was used. The quality of the evidence was evaluated using GRADE.

## Results

The search strategy recovered 102 articles, and only five met the inclusion criteria. Meta-analysis showed an OR = 2.94 (95% CI = 1.83 – 4.74; p<0.01) for the prevalence of CAP among patients with atherosclerosis. The risk of bias was moderate. Quality of the evidence showed a low level of certainty.

## Conclusions

Patients with atherosclerosis are almost three times more likely to have CAP. This result should be translated to daily clinical practice: the healthcare community should be aware of this association and suspect atherosclerotic pathology in patients who show a high prevalence of CAP. Patients with atherosclerosis should be monitored in the dental clinic for CAP.

# Abstract: 01. Modified socket shield technique: a novel approach to immediate implant placement and esthetic optimization

**Authors:** G. Khachatrian, E. Zernitckaia

**Affiliations:** *“Cosmodent” Dental Clinic, Tomsk, Russia. gurgen@mail2000.ru*

## Aim

The rehabilitation of esthetic areas using immediate post-extraction implants has become a predictable procedure with high survival rates. However, achieving optimal esthetic outcomes, particularly in the anterior maxilla, remains challenging due to physiological bone resorption and soft tissue contraction after tooth extraction. The socket shield technique (SST), first introduced by Hurzeler et al., offers a novel approach by preserving the buccal root segment to maintain periodontal ligament vascularization, reducing buccal bone resorption, and improving esthetic results. This study evaluates the outcomes of a modified SST, which includes the creation of a buccal shield in a C-shape and the use of autologous bone graft in the implant gap. To assess the implant survival rate, esthetic outcomes, and complication rates of a modified SST compared to the classic SST and immediate implant placement.

## Material and Methods

A prospective clinical study was conducted with 30 patients, divided into three groups of 10 each: - Group A: Modified SST with a 1–1.5 mm buccal shield, C-shaped root preparation, and autologous bone grafting. - Group B: Classic SST. - Group C: Immediate implant placement. Pre-surgical diagnostics included CBCT and intraoral scanning. Implants were placed using a digitally guided protocol. Clinical and radiographic follow-ups were performed at pre-prosthetic and 1-year stages, measuring Pink Esthetic Score (PES), buccal bone thickness, and marginal bone loss. Statistical analysis was applied to compare outcomes across groups.

## Results

Implant survival rates were high across all groups, with Group A demonstrating superior esthetic outcomes compared to Group B and Group C. Group A also showed significantly less buccal bone resorption and marginal bone loss at 1 year (p<0.05).

## Conclusions

The modified SST offers a promising alternative for immediate implant placement in esthetic zones. By reducing complications and preserving hard and soft tissue architecture, it provides superior esthetic results and enhances the predictability of anterior implant rehabilitation. Further long-term studies are needed to validate these findings.

# Abstract: 02. Rate of progression of initial proximal carious lesions in high caries risk adult patients

**Authors:** Q. Alomari, I. Alfarhan, A. Faridoun

**Affiliations:** *Kuwait University, Jabriya, Kuwait. qasem.alomari@ku.edu.kw*

## Aim

The aims of this study were to investigate the radiographic progression of proximal enamel and outer dentin carious lesions in high caries risk adult patients attending Kuwait University Dental Center (KUDC) and to evaluate the factors associated with this progression.

## Material and Methods

Serial bitewing radiographs were retrospectively analyzed from 105 patients (who met the inclusion criteria), aged 18-61 years, for the progression of 364 enamel and outer dentin proximal carious lesions. The difference in time between the set of radiographs was recorded in months. The patients’ demographic data (age and gender), initial plaque score, initial bleeding on probing (BOP) score, initial fluoride varnish treatment, DMFT, and DMFS scores were collected from the patients’ electronic dental records. Lesions’ progression with respect to all variables was analyzed with the Mann-Whitney rank sum and the Kruskal-Wallis tests. Additionally, multivariate logistic regression analysis with caries progression as the dependent variable was performed.

## Results

Caries progression was only evident in 112 lesions (30.77%). No lesion progressed from enamel to inner dentin and only 12 lesions (out of 43) progressed from outer dentin to inner dentin. There was a statistically significant difference in the rate of lesion progression according to tooth location (upper/lower jaw), duration between the first and second radiographs, and initial lesion depth (p<0.05). According to multivariate logistic regression analysis, lesions in the upper jaw were more likely to progress deeper than lesions in the lower jaw (odds ratio 1.63). When the duration between the first and second radiographs was > 18 months, the lesion was 2.18 times more likely to progress than when the duration ≤ 18 months. There was a statistically significant difference in the distribution of lesion depth between the first and second radiographs (p<0.001).

## Conclusions

About one-third of the initial proximal carious lesions among high caries risk adult patients exhibited progression. No lesions progressed from enamel to inner dentin and only about 30% of lesions located in the outer dentin initially were the only ones that progressed to inner dentin. Tooth location (upper jaw), duration between baseline and follow-up radiographs, and initial lesion depth were the only factors significantly associated with lesion progression.

# Abstract: 03. Tooth wear and depression symptoms – no direct correlation identified

**Authors:** K. Ahmed, S.K. Tadakamadla

**Affiliations:** *University of Melbourne, Melbourne, Australia. khaled.ahmed@unimelb.edu.au*

## Aim

To evaluate the relationship between major depressive disorder (MDD) symptoms and pathological tooth wear severity among patients at a university dental clinic seeking management for their tooth wear.

## Material and Methods

This six-month prospective study assessed tooth wear patients using the Basic Erosive Wear Examination (BEWE) index, scored by four calibrated examiners. Participants completed the Patient Health Questionnaire-9 (PHQ-9) to assess MDD presence and severity. Demographic data, medical history, mental health status and postcodes were recorded. Statistical analysis included Shapiro-Wilk tests for normality, linear regression analysis, t-tests and Chi-square tests to examine associations between BEWE scores and depressive symptoms, gender, socioeconomic status and posterior occlusal support.

## Results

The study included 66 participants (71% male; 64% >60 years). Sixteen patients lacked posterior support in at least one sextant. Most participants (83%) exhibited minimal depressive symptoms (PHQ-9 score <10) and demonstrated average BEWE scores ≥2 (n=58). While posterior support significantly correlated with average BEWE scores (p=0.010), no significant associations were found between BEWE scores and PHQ-9 scores (p=0.242), gender (p=0.317), or age (p=0.692). A trend toward more severe anterior wear (0.4 points higher on BEWE scale) was observed in patients lacking posterior support, though this difference was not statistically significant.

## Conclusions

This study found no significant association between depressive symptoms and tooth wear severity. However, reduced posterior occlusal support was significantly associated with increased tooth wear severity in this patient population. Further research with larger sample sizes may be warranted to confirm these findings.

# Abstract: 04. 10 year prospective clinical evaluation on direct pulp protections for caries in adults

**Authors:** M. Boldrini

**Affiliations:** *University of Buenos Aires, Buenos Aires, Argentina. myriam.boldrini@odontologia.uba.ar*

## Aim

The aim of this clinical study has been to evaluate pulp vitality after performing different pulp capping procedures for caries removal in adult patients. evaluating them for ten years clinically and radiographically.

## Material and Methods

Adult patients with caries in molars and have been treated by the same operator, the protocol was always based on absolute isolation opening, removal of caries and in the face of partial pulp exposure it has been decided randomly to perform G1 protection with calcium hydroxide G2 partial pulpotomy with hydroxide calcium G3 partial pulpotomy and MTA. All were restored with adhesive techniques.

## Results

The data were recorded dichotomously in a longitudinal registry (1: Vital; 0: Non-vital). Estimation of survival time: Kaplan Meier analysis Log Rank comparison (Mantel–Cox).The loss of pulp vitality was established as an end point (0) Average (years) • G1: 4.7 (CI95= 3.7 - 5.8) • G2: 10 (IC95= 8.8 – 10.0) • G3: 5.6 (CI95= 3.4- 7.8). The survival 10 years rate is approximately 95% in group 2.

## Conclusions

MTA hardens in the presence of humidity allows for better sealing and the results are better compared to HC, MTA. Pulp capping with MTA produces cytological and functional changes of the pulp cells, resulting in the production of reparative dentin on the surface of an exposed pulp. MTA offers a biologically active substrate for pulp cells, necessary to regulate dentinogenic events. The immediate reaction indicates the stimulation of the biosynthetic activity of pulp cells by the coating, but this cannot be taken as a direct induction of formation of reparative dentin. A new matrix of a tubular forms with cellular inclusions is observed under the MTA at two weeks while reparative dentinogenesis is20/12/202520/12/202520/12/2025 obtained four weeks after coverage. Therefore, MTA is a more effective material than HC in direct pulp coverage, favoring the formation of a continuous hard tissue bridge if the procedure is done under asepsis.

# Abstract: 05. The influence of preparation design and resin type on the marginal adaptation of 3D printed resin onlay restorations

**Authors:** S.N. Yigin, S. Akarsu

**Affiliations:** *Department of Restorative Dentistry, Faculty of Dentistry, Ordu University, Altınordu/Ordu, Turkey. senanurygn@gmail.com*

## Aim

This study aims to compare the marginal adaptation of onlay restorations fabricated from two different 3D printed resins with two different preparation techniques in plastic teeth.

## Material and Methods

This study used two different 3D printed crown resins (Crowntec, Saremco Dental AG, and VarseoSmile Crown Plus, Bego) to produce 40 restoration samples. Two different preparation designs were applied for the two resins, dividing the samples into four groups: Group 1: VarseoSmile + Shoulder, Group 2: VarseoSmile + Flat, Group 3:Crowntec + Shoulder, Group 4:Crowntec + Flat. The samples were prepared in accordance with the design rules for shoulder and flat preparation. Prepared models were scanned with a 3D intraoral scanner (Trios, 3Shape, Copenhagen, Denmark). Designs were created using CAD software (ExoCAD 2.4 Plovdiv; ExoCAD GmbH, Darmstadt, Germany). Chitubox V1.7.0 (Guangdong, China) 3D printing software was used to set a 50 μm layer thickness, a 0° print orientation and the models were sliced. Restorations were fabricated with a 3D printer (Phrozen sonic mini 8K SLA device, Phrozen Technology, Hsinchu City, Taiwan). Restorations was examined under a stereomicroscope (Leica S6 D; Leica Microsystems Inc) at x40 magnification. Statistical analysis was performed using one-way ANOVA and Tukey's HSD posthoc tests.

## Results

Statistical analysis revealed that those produced with Crowntec (Group 3-4) showed better marginal adaptation than those produced with VarseoSmile (Group 1-2). There is a statistically significant difference. Although the flat groups showed better marginal adaptation than the shoulder groups, no statistically significant difference was found. The highest marginal adaptation was observed occlusal, while the lowest adaptation was observed mesial.

## Conclusions

Resin type and location may have an influence on the marginal adaptation of onlay restorations, but further studies are necessary.

# Abstract: 06. Determination of observer compatibility and accuracy among fluorescence-aided, visual and radiographic caries diagnostic methods

**Authors:** B.S. Kalafatoglu, Y. Yasa, A. Sahin, S. Baglar

**Affiliations:** *Department of Restorative Dentistry, Faculty of Dentistry, Ordu University, Altınordu/Ordu, Turkey. b.serenaykalafatoglu@odu.edu.tr*

## Aim

The aim of this study was to compare the effectiveness of the Proface [Fluorescence-Aided Caries Excavation (W-H, Austria)] device, visual examination, and bite-wing radiography in detecting dental caries and to evaluate the inter-observer agreement of these methods. The null hypothesis of the study were, there is no significant disagreement among diagnostic methods, and no significant inconsistency among observers.

## Material and Methods

From 114 patients a total of 443 first and second molars which were intact and unrestored were examined in the study. Three diagnostic methods were assessed by two independent observers. Proface (W-H, Austria) evaluation was based on red reflection: 0-no reflection; 1-reflection present. Visual examination scored teeth as: 0-no caries; 1-lesion visible after drying; 2-lesion visible without drying; 3-gray discoloration. Bite-wing radiography categorized teeth as: 0-no caries; 1-enamel caries; 2-dentin caries. Inter-observer reliability of each detection method was determined using Cohen's Kappa analysis and the relationship among methods were analyzed using the Chi-square test.

## Results

According to Cohen's Kappa analysis, the highest interobserver agreement was achieved with the Proface (W-H, Austria) method (Kappa=0.721, good agreement). Bite-wing radiography showed good agreement (Kappa=0.689), while visual examination demonstrated moderate agreement (Kappa=0.537). Chi-square analysis demonstrated that the relationship between the Proface (W-H, Austria) method and Bite-wing radiography was significant (p<0.001). There was also statistically significant relationship between the Proface method and visual examination (p<0.001).

## Conclusions

For caries diagnosis, using Proface (W-H, Austria) is an adequate alternative to traditional methods. Thereupon, using these methods in combination may improve caries detection accuracy.

# Abstract: 07. The effect of selective carious removal on the bond strength of bioactive restorations

**Authors:** I. Halici Ortega Guardia, B. Oglakci Ozkoc, M. Ocak, E. Alkan, D. Tagtekin

**Affiliations:** *Department of Restorative Dentistry, Faculty of Dentistry, Bezmialem Vakif University, İstanbul, Turkey. irmak_halici@hotmail.com*

## Aim

This in vitro study evaluated the effect of selective carious removal on the microtensile bond strength (μTBS) of bioactive restorations.

## Material and Methods

48 molars were used with standardized Class I cavity preparations and randomly divided into 6 groups based on restorative Material (bioactive-alkasite and giomer, conventional-nanohybrid composite) and caries removal method (selective/non-selective): Group 1) Selective caries removal+alkasite (Cention Forte); Group 2) Selective caries removal+giomer (Beautifil II); Group 3) Selective caries removal+nanohybrid composite (Estelite Posterior); Group 4) Non-selective caries removal+alkasite (Cention Forte); Group 5) Non-selective caries removal+giomer (Beautifil II); Group 6) Non-selective caries removal+nanohybrid composite (Estelite Posterior). To create artificial dentin caries, pulpal floor of cavities were demineralized with acetic acid for 35 days (pH=4.5) with a shaking incubator (60 rpm, 37 C). In non-selective caries removal groups, softened dentin was removed with an excavator until hard, dry dentin. In selective caries removal groups, softened dentin was left untreated. Conventional methods, DIAGNOdent Pen, Micro-CT and microhardness tests were used to determine mineral values at different time points. After restorations, they were stored in artificial saliva for 1 month to ensure bioactivity potential and ageing of restorative materials. μTBS test was conducted using a universal testing machine (crosshead speed:0.5 mm/min) (n=20). Fractured surfaces were examined with a stereomicroscope. Dentin-restoration interfaces were analyzed under SEM/EDS. Two-way ANOVA and Bonferroni tests were performed for statistical analysis(p<0.05).

## Results

For all restorative materials, non-selective caries removal groups (Groups 4,5,6) (27.45±6.84, 27.55±9.01, 22,12±4,23) showed significantly higher μTBS compared to selective caries removal groups (Groups 1,2,3) (20.55±10.56, 17.93±8.92, 14,03±7,36) (p<0.05). In terms of caries removal method, no significant differences in μTBS were observed among restorative Material (p<0.05). For non-selective removal groups, SEM images with an intact interface and higher number of mixed failures confirmed greater bond strength values.

## Conclusions

Non-selective caries removal could enhance bond strength of bioactive restorations, potentially offering a more reliable tooth-restoration interface.

# Abstract: 08. The influence of direct restoration on trabecular bone structure: a fractal analysis approach

**Authors:** E. Yakin, K. Orhan

**Affiliations:** *Department of Restorative Dentistry, Faculty of Dentistry, Ankara University, Ankara, Turkey. dteceokten@gmail.com*

## Aim

This study aimed to investigate the influence of direct restorations on the microarchitecture of mandibular trabecular bone. The structural integrity of trabecular bone in the mandibular region can be affected by various factors, including tooth loss, restorative treatments, and occlusal forces. Understanding the potential impact of direct restorations on the microstructural properties of the surrounding bone and tissues is crucial for ensuring long-term biomechanical stability. The null hypothesis tested in this study posits that there is no statistically significant difference in trabecular structure between restored and natural tooth regions. In other words, it assumes that direct restorations do not induce notable changes in the microarchitecture of mandibular trabecular bone. However, clinical and micromorphological analyses play a critical role in evaluating the validity of this hypothesis. By examining the bone's adaptive response following restorative procedures, this research can provide valuable insights into how restorative interventions interact with the surrounding bone structure. The findings may contribute to more informed decisions regarding material selection and restorative techniques in clinical practice. Additionally, the results could offer new perspectives on the relationship between long-term restorative success and bone integrity, helping to optimize biomechanical and biological compatibility.

## Material and Methods

A comparative analysis was performed using a total of 150 panoramic radiographs, aiming to evaluate the trabecular bone structure surrounding restored teeth and compare it to their contralateral, unrestored counterparts in the same patients. Each radiograph provided a bilateral perspective, allowing for an in-depth assessment of potential differences in trabecular microarchitecture between restored and natural regions. To achieve this, fractal analysis was utilized as a quantitative method to measure bone complexity and assess trabecular patterns. Fractal analysis is a well-established mathematical approach used in radiographic studies to characterize the structural intricacies of bone and detect subtle variations in trabecular organization (Updike - Nowzari, 2008). This technique enables the evaluation of bone density and connectivity by analyzing repetitive patterns within the trabecular framework, providing an objective measure of microstructural alterations. By applying this method, the study aimed to determine whether direct restorations influence trabecular bone remodeling processes and whether these changes, if present, differ significantly from those observed in the unrestored contralateral region. The findings from this analysis could contribute to a deeper understanding of the biomechanical and biological responses of mandibular trabecular bone to restorative interventions, offering potential implications for clinical decision-making in restorative dentistry.

## Results

A two-way ANOVA statistical analysis was conducted to evaluate differences in overall trabecular bone structure between restored and unrestored regions. The results indicated that there were no statistically significant differences (p=0.05) in trabecular bone characteristics between the two groups. This suggests that direct restorations did not produce notable alterations in trabecular microarchitecture when compared to their contralateral, unrestored counterparts. To further explore potential variations, an additional stratified analysis by gender was performed. This secondary analysis aimed to determine whether biological differences between male and female patients might influence trabecular bone adaptation in response to restorations. However, the results demonstrated no statistically significant differences between genders, indicating that the absence of structural alterations was consistent across both male and female groups.

## Conclusions

These findings suggest that direct restorations, as employed in this study, do not induce substantial alterations in the surrounding mandibular trabecular bone microarchitecture. This observation supports the notion that the bone tissue in the region adjacent to restored teeth maintains a comparable level of structural complexity to those surrounding unrestored teeth. This research contributes to a deeper understanding of how bone tissue responds to restorative interventions, specifically in the context of direct restorations. The absence of significant changes in trabecular structure suggests a degree of resilience or adaptability in the bone tissue, maintaining its integrity despite the presence of the restoration. However, it's important to acknowledge the limitations of this study, such as the cross-sectional design and the specific type of restoration examined. Future investigations could explore the influence of different restorative materials, the long-term effects of restorations through longitudinal studies, and the application of alternative analytical techniques, such as micro-computed tomography, to further elucidate the complex interplay between dental restorations and bone health. Such research could provide a more comprehensive understanding of the bone tissue response to various restorative approaches and materials, ultimately leading to improved clinical practices and patient outcomes.

# Abstract: 09. Degree of conversion of different types of composite resins polymerized with a high-power led curing device

**Authors:** G. Ozciftci, H. Boyacioglu, L.S. Turkun

**Affiliations:** *Department of Restorative Dentistry, Faculty of Dentistry, Ege University, İzmir, Turkey. gozde.ozciftci@gmail.com*

## Aim

The aim of this study is to evaluate the degree of conversion of different composite resins polymerized using rapid (3s) and conventional polymerization settings (10s and 20s) of a Light Emitting Diode curing unit.

## Material and Methods

Cylindrical samples (3 x 4-mm) were prepared from Tetric PowerFill (PFL; Ivoclar, Vivadent, Liechtenstein), Tetric PowerFlow (PFW; Ivoclar, Vivadent) and Omnichroma Bulk Flow (OB; Tokuyama, Japan) and divided in three groups (n=10) according to polymerization settings: 3s-RP, 10s-CP and 20s-CP. All specimens were irradiated with a Light Emitting Diode curing unit (Bluephase PowerCure, Ivoclar Vivadent). A Fourier Transform Infrared Spectroscope (FTIR; Perkin Elmer, Waltham, USA) operating within a 400-4000 cm⁻¹ wavelength range was used to determine the upper and lower degree of conversion (DC) of the samples. The DC% of the composite resin is thought to be determined by the number of double carbon bonds converted to single bonds. Non-reactive carbon–carbon double bonds (C=C%) were determined using the ratio of the aliphatic C=C (1637 cm-1) and aromatic C–C (1608 cm-1) absorbance densities. FTIR spectra of both non-polymerized and polymerized samples were analyzed. The measurements were recorded as absorption spectra. Intra-group comparison was performed with T test and material/upper-lower comparison was performed with ANOVA and Tukey tests (p<0.05).

## Results

All group comparisons were found to be significant for both upper and lower DCs (p<0.05). PFL groups have better upper and lower DC after 3s curing while for PFW this time was 10s and was 20s for OB groups (p<0.05). While material groups and upper-lower DC were compared together, only 10s values were found to be non-significant.

## Conclusions

Rapid 3s curing time is found to be appropriate only for PFL groups while 10s curing time is found to be a better option for PFW. Regular bulk-fill composite resins have better DC after 20s curing time.

# Abstract: 10. Microtensile bond strength evaluation of Riboflavin-5'-phosphate on sound and caries affected dentin

**Authors:** E. Can, M. Berkman, Z. Çiftgül, S. Imarlı

**Affiliations:** *Department of Restorative Dentistry, Faculty of Dentistry, Yeditepe University, Istanbul, Turkey. esra.cansay@yeditepe.edu.tr*

## Aim

The purpose of this study was to evaluate the effect of Riboflavin-5'-phosphate (Riboflavin) on the microtensile bond strength (μTBS; MPa) of a two-step self-etch adhesive to sound (SD) and caries-affected dentin (CAD).

## Material and Methods

52 extracted human molar teeth with occlusal caries were ground flat to expose SD and CAD using 180 SiC paper (Metaserv 250, Buehler, Germany). Caries-infected dentin was removed with 600-grit SiC paper using visual, tactile, and caries-detecting dye methods. Then, all the SD and CAD specimens were randomly divided into two groups according to the Riboflavin application. In groups without Riboflavin, the two-step self-etch adhesive (Clearfil SE Bond, Kuraray, Japan) was applied to SD and CAD specimens according to the manufacturer’s instructions, while in groups with Riboflavin SD and CAD specimens were treated with 0.1% Riboflavin (MedioCROSS-H, Simovision, Netherlands) using a micro-brush for 20 seconds and activated by an LED curing device (Bluephase, Ivoclar Vivadent, Lichtenstein) for 20 seconds. Then, Clearfil SE Bond was applied per the manufacturer’s instructions. A micro-hybrid resin composite (Filtek Z250, 3MESPE, USA) was used for composite build-ups which were subsequently immersed in 37°C distilled water for 24 hours and cut into 1mm² sticks. μTBS was then evaluated with a universal testing device (Instron, 3345, Instron Corp., USA) at a 0.5 mm/min crosshead speed. Data were analyzed with Two-way ANOVA and Tukey HSD tests (p<0.05).

## Results

Regardless of Riboflavin application, μTBS to CAD was significantly lower than to SD (p<0.05). Riboflavin application resulted in significantly higher μTBS to CAD (23.51±4.51) than CAD without Riboflavin (17.24±6.82), while there were no significant differences between μTBS to SD with and without Riboflavin application (29.81±6.21 and 27.33±6.89 respectively).

## Conclusions

Riboflavin application showed a beneficial effect on the short-term μTBS of a two-step self-etch adhesive to caries-affected dentin, which should be confirmed with long-term studies.

# Abstract: 11. Effects of caries removal techniques and cavity pretreatments on dentin: cross-sectional hardness and color staining analysis

**Authors:** O.E. Pecho, L.F. Rondón, A. Rastelli

**Affiliations:** *School of Dentistry, University of Granada, Granada, Spain. oscarpecho@ugr.es*

## Aim

To evaluate the effects of different caries removal techniques (dentin excavator, spherical bur, and ultrasound) and the combination of cavity pretreatments (chlorhexidine, antimicrobial photodynamic therapy, and control group) on hardness and staining susceptibility of dentin. The null hypotheses tested were that there are no significant differences in (i) cross-sectional hardness, and (ii) staining susceptibility after the different approaches for remotion of dentin caries-like lesions.

## Material and Methods

Bovine dentin specimens (N=201) were prepared and subjected to caries induction using a Streptococcus mutans and Actinomyces naeslundii biofilm model. The removal of dental caries was conducted using a dentin excavator, spherical bur, and ultrasound. The specimens were then treated with either chlorhexidine (0.2%), antimicrobial photodynamic therapy using curcumin (60 J/cm²), or no treatment (control). For cross-sectional hardness evaluation, samples (n=9) were assessed using a microhardness tester (HMV-2, Shimadzu) at five different 40-μm interval depths (40, 80, 120, 160, and 200 μm). For color measurement, samples (n=20) were evaluated using a spectrophotometer (CM-2600d, Konica Minolta). Thus, CIELAB parameters were obtained before and after each cavity pretreatment, and the color difference was obtained using CIEDE2000 color difference formula (ΔE00). Data were analyzed using ANOVA and Tukey’s post-hoc test (α = 0.001). Perceptibility (PT) and acceptability (AT) thresholds were used (PT=0.81 and AT=1.77).

## Results

Cross-sectional hardness increased progressively with depth across all groups. Spherical bur group and ultrasound group showed the highest and the lowest values, respectively (p≤0.001). Chlorhexidine exhibited the lowest ΔE00 values across all removal techniques, remaining below the acceptability threshold (≤1.8 ΔE00 units). In contrast, antimicrobial photodynamic therapy-treated specimens showed significantly higher ΔE00 values (p≤0.001), the combination ultrasound-antimicrobial photodynamic therapy showed the highest color changes (17.22±0.91).

## Conclusions

Caries removal and cavity pretreatments influenced cross-sectional hardness at different depths of dentin. Cavity pretreatments affected staining susceptibility of dentin.

# Abstract: 01. Effect of different remineralization agents on microhardness and shear bond strength of enamel

**Authors:** A. Kaya, S. Aktug Karademir

**Affiliations:** *Department of Restorative Dentistry, Faculty of Dentistry, Ordu University, Altınordu/Ordu, Turkey. aysgl08@hotmail.com*

## Aim

This study aimed to investigate the effects of remineralization agents on enamel surface microhardness (VHN) and shear bond strength (SBS).

## Material and Methods

In the study, 100 human premolar teeth were used. Each tooth was sectioned buccolingually into two pieces and the buccal pieces were covered with nail polish leaving a 3x3 mm area open. The samples were randomly divided into five groups (n=20). Group 1, consisting of sound enamel, served as the positive control. Artificial caries lesions were induced in all other groups by immersing the samples in 0.5% citric acid twice daily for 30 minutes over three days. Group 2, comprising demineralized enamel, served as the negative control. The samples in Group 3 (CCP-ACPF paste), Group 4 (5% NaF varnish), and Group 5 (NovaMin toothpaste) underwent the respective remineralization treatments for one week, following the manufacturer's instructions. After measuring the VHN of the samples using a Vickers hardness tester, a 3x3x2 mm universal composite resin was applied to the enamel surface. Then, the SBS of the samples was tested using a universal testing machine at a crosshead speed of 0.5 mm/min. Failure types were examined using a stereomicroscope. The data were statistically analyzed using SPSS software (p<0.05).

## Results

The VHN (202.61±15.77) and SBS (11.81±3.69) were the lowest in group 2. In all remineralization groups, VHN and SBS were significantly higher than in group 2 (p<0.05), but lower than in group 1. There wasn’t significant difference in SBS among remineralization groups. Adhesive failure was significantly higher in group 2 compared to the other groups (p<0.05).

## Conclusions

It was concluded that remineralization agents are beneficial in increasing the microhardness of demineralized enamel and significantly affect the bond strength and failure modes of composite resins to enamel.

# Abstract: 02. Usage frequency and perspective of academic dental practitioners on dental magnification devices: A survey study

**Authors:** S.G. Kuzucan, E. Kitin, A. Pekgoz, O. Yaziciogli

**Affiliations:** *Department of Restorative Dentistry, Faculty of Dentistry, Istanbul University, Istanbul, Turkey. seragulhankuzucan@gmail.com*

## Aim

This study aimed to evaluate the perspectives and usage frequency of digital magnification devices (DMDs) among specialization, doctoral students, and academic staff at the Faculty of Dentistry, Istanbul University.

## Material and Methods

The survey included 159 participants consisting of specialization and PhD students and academic faculty members. The survey, comprising 18 multiple-choice questions, was distributed via email and a QR code link. Statistical analyses, including chi-square tests, were conducted at SPSS v30 to evaluate relationships between categorical variables, with significance set at p<0.05.

## Results

Participants comprised 65 males (40.9%) and 94 females (59.1%), predominantly aged 25–34 years (54.7%). Professors constituted 6.3% of the participants, 11,3% were associate professors, 14,4% were assistant professors, and the remaining percentage (%67,9) consisted of doctoral and specialization students. Approximately 49.4% reported prior use of dental loupes, while 50.6% were non-users. The Cost was identified as the main barrier to acquire a dental loop by 83.6% of participants. Among users, Galilean loupes were more popular (35.2%) compared to prismatic loupes (25.2%). Most respondents (80%) acknowledged that DMDs improved visual comfort, and 88% agreed they enhanced treatment quality, particularly in endodontics (83.6%) and restorative dentistry (64.2%). Concern about being dependent on the device was noted by 58.4%.

## Conclusions

The findings highlight a growing awareness and utilization of DMDs in dental practice, drawing attention to their ergonomic benefits and capacity to enhance precision and treatment quality. Addressing barriers such as cost and dependency may further promote their adoption. Encouraging the integration of DMDs in dental education could foster early familiarity and optimize their use in clinical settings.

# Abstract: 03. Zoom on caries risk assessment: A critical scoping review

**Authors:** S. Rouijel, B. Rémi, B. Touré, S. Doméjean

**Affiliations:** *Health Sciences Research Center, Faculty of Dental Medicine, International University of Rabat, Rabat, Morocco. soukaina.rouijel@uir.ac.ma*

## Aim

Caries risk assessment (CRA) can be used for monitoring the status of oral care, as well as for documentation and research purposes. Numerous CRA systems have been developed, generally based on a combination of factors designed to assess the propensity for the initiation and development of dental caries. The aim of this study was to compile the factors involved in various CRA systems, available in English or French, for children and/or adults, and subsequently evaluate their frequency of use and their classification.

## Material and Methods

The most exhaustive list of CRA systems, in English or French, was assembled based on a manual search via Pubmed and Google. For each identified CRA system, the followings were recorded: country of origin, patient profile, name and number of domains considered, factors considered and name of the domain(s) to which they were linked, as well as method of caries risk level classification.

## Results

A total of 29 different CRA systems and 44 assessment domains was identified, encompassing 338 distinct factors (differences between them may be subtle). The analysis of these CRA systems highlights a significant heterogeneity. Moreover, most of the factors are linked to multiple domains, creating a somewhat disorganized network. Even different methods of caries risk level classification were also identified.

## Conclusions

The variability in terminology across CRA systems here described underscores the necessity for standardization in CRA at several levels: definition of factors to be taken into account, domain categorization, and method of caries risk level classification.

# Abstract: 01. Endodontic Management of an Extraoral Sinus Tract Originating from #26 on the Left Buccal Region

**Authors:** S.J. Park, J.H. Shin

**Affiliations:** *Department of Conservative Dentistry, Korea University Guro Hospital, Seoul, Korea. sssji0203@gmail.com*

## Introduction

This case report highlights the diagnosis and nonsurgical treatment of an extraoral sinus tract caused by chronic apical periodontitis, emphasizing the importance of recognizing dental infections often misdiagnosed as dermatologic conditions.

## Case Report

A 39-year-old man was referred from oral and maxillofacial surgery for a boil in the left buccal region after prior evaluations by dermatology. Clinical examination revealed induration in the buccal vestibule near tooth #25 with severe distal caries and a gold crown-restored #26. The patient reported undergoing RCT on #26 about 7 years earlier. Radiographic and CBCT imaging revealed an apical lesion on #26, with underfilled canals and a missed MB2 canal, confirming it as the infection source. Tooth #26, diagnosed as a previously treated tooth with chronic apical periodontitis, was managed with endodontic retreatment. The working length was measured with an apex locater, and the canals were cleaned and shaped using NiTi rotary files (ProTaper Gold), irrigated with 5.25% NaOCl and 17% EDTA, and filled with gutta-percha and AH+ sealer using continuous wave technique. It was restored with resin core and Zirconia crown placement. During caries removal on tooth #25, pulp exposure with uncontrolled bleeding was observed. RCT was performed using a similar protocol to tooth #26, followed by post, resin core, and Zirconia crown restoration. The extraoral sinus tract gradually diminished with each visit, reducing to a small scar by the time crown restoration was completed. A Mona Lisa lidocaine filler was injected for cosmetic enhancement. At the 1-year follow-up, the apical lesion had resolved, and the scar appeared as a slight dimple when smiling.

## Conclusions

Dental infections should be considered when diagnosing cutaneous sinus tracts on the face or neck. Clinical and radiographic evaluations, including CBCT, are essential to identify the causative tooth and avoid unnecessary treatments.

# Abstract: 02. 180-degree rotation intentional replantation of a maxillary premolar with subgingival palatal cusp fracture

**Authors:** J.Y. Kim, B. Shin, J.Y. Yoon

**Affiliations:** *Seoul National University Bundang Hospital, Seoul, South Korea. kjy4549@naver.com*

## Introduction

Palatal cusp fractures in maxillary premolars can be challenging to treat, particularly when the fracture extends subgingivally and invades the biological width. Traditional treatment options such as extraction, crown lengthening, or orthodontic extrusion each have limitations. Crown lengthening may lead to an unfavorable crown-to-root ratio, while orthodontic extrusion is time-intensive. Intentional replantation with 180-degree rotation offers a promising alternative, allowing the repositioning of intact tooth structure onto the functional cusp.

## Case Report

A 69-year-old male presented with a fractured palatal cusp of the left first premolar (#24), extending 3mm subgingivally. After discussing various treatment options, intentional replantation with 180-degree rotation was selected. To facilitate atraumatic extraction, resin MTA tubes and lingual buttons were placed on the buccal surfaces of teeth #23, #24, and #25. Two weeks later, tooth #24 was extracted using Physics Forceps®, and after confirming the absence of root cracks or resorption, the tooth was replanted following a 180-degree rotation with the buccal margin at the supragingival level. A resin-wire splint was applied to stabilize tooth #24 with #25. After two weeks, root canal treatment was performed, followed by a temporary filling with Riva Light Cure® material. One week later, a resin core buildup was completed, and a temporary crown was placed. One month later, the patient showed no signs of percussion pain or biting discomfort, and the treatment was completed with a zirconia crown.

## Conclusions

Surgical extrusion with 180-degree rotation is an effective technique for managing palatal cusp fractures in maxillary premolars, particularly when the fracture extends subgingivally. This method preserves tooth structure and minimizes bone removal. Key factors for success include appropriate case selection, atraumatic extraction with preoperative orthodontic treatment, and proper stabilization of the replanted tooth. With these factors in place, this approach provides a reliable alternative to more conventional treatments, ensuring functional restoration.

# Abstract: 01. Restoration of a subgingivally fractured tooth with orthodontic extrusion using a Mini-Tube Appliance: A Case Report

**Authors:** H. Yang, Y. Shin, J.W. Park, D. Kim

**Affiliations:** *Department of Conservative Dentistry, College of Dentistry, Yonsei University, Seoul, South Korea. yanghj422@yuhs.ac*

## Introduction

The mini-tube appliance (MTA) typically used for partial correction of anterior teeth, can also be applied in the extrusion of fractured teeth. We present a case of an orthodontic extrusion of a subgingivally fractured maxillary first premolar using the MTA.

## Case Report

A 28-year-old male reported to the Department of Conservative Dentistry with the chief complaint of tooth fracture. Clinical and radiographic examinations revealed a 2mm sub-gingival fracture on the mesial and distal sides. The treatment plan included orthodontic extrusion followed by post-placement and zirconia crown restoration. For the first step, root canal treatment was performed under rubber dam isolation. The mini-tube appliance (HUBIT, Republic of Korea) was applied to teeth #13-15, and a 012 Ni-Ti MTA wire (HUBIT, Republic of Korea) was passively inserted. A palatal fixed retainer using 08 stainless steel wire was placed on tooth #15-17 to prevent shifting of adjacent teeth. The patient was checked weekly, with supracrestal fiberotomy performed on tooth #14. Orthodontic extrusion was applied for 3 weeks, extruding 3.0 mm to expose the fracture above the gingiva to secure ferrule for dentin. A fiber-reinforced composite post and resin core restoration were placed. A fixed retainer using 05 stainless steel wire was bonded for 6 weeks for stabilization. Following this, a zirconia crown was placed, and an additional 4-week retention period was observed. As a result of the 6-month clinical follow-up, the extruded tooth remained stable without relapse also the restoration was well-maintained without any periodontal problems.

## Conclusions

In this case, orthodontic extrusion was performed to restore a subgingivally fractured tooth, using the mini-tube appliance (MTA). The MTA consists of a small, approximately 3 mm tube that minimizes foreign body sensation and discomfort. It offers aesthetic advantages with a lower dropout rate, and the appliance's low frictional resistance facilitates efficient tooth movement with only light wire forces.

# Abstract: 02. Aesthetic rehabilitation of hypomineralized maxillary anterior teeth with deproteinization pre-treatment: A case report

**Authors:** S.H. Cho, J.W. Park, E. Kim

**Affiliations:** *Department of Conservative Dentistry, College of Dentistry, Yonsei University, Seoul, South Korea. sayhyeon@yuhs.ac*

## Introduction

The high organic content of hypomineralized enamel hinders effective bonding. This case demonstrates the use of sodium hypochlorite to dissolve proteins, thereby improving adhesion to hypomineralized enamel. A 12-year-old female patient presented with hypersentivity and dissatisfaction with the appearance of her upper anterior teeth. The teeth were diagnosed as enamel hypomineralization with dental caries. Composite resin restoration was planned to address hypersensitivity and aesthetic concerns.

## Case Report

The most hypersensitive maxillary right central and lateral incisors were treated first. Hypomineralized enamel and carious dentin were removed until a hard tactile sensation was felt with a dental explorer. Deeper hypomineralized enamel was preserved to avoid pulpal irritation. The cavosurface margin was placed on sound tooth structure to prevent bonding failure. After etching with 37% phosphoric acid (3M ESPE, USA), deproteinization was performed by applying 6% sodium hypochlorite solution (SNS Dental, Korea) for 60 seconds. Rubber dam application was required to prevent soft tissue irritation. A self-etch Clearfil SE Bond adhesive (Kuraray, Japan) was applied. Estelite Sigma Quick (Tokuyama, Japan) opaque A2 shade was first used to mask the remaining white spot of hypomineralized enamel, followed by A1 and A2 shades for restoration. Polishing was done with Sof-Lex discs (3M ESPE, USA). The same procedure was repeated for the maxillary left incisors and both canines. At the one-week follow-up, the patient was satisfied with the outcome and experienced reduced sensitivity to cold. Routine follow-ups over the next three months showed no significant abnormalities.

## Conclusions

Congenitally hypomineralized enamel is commonly seen in clinics. As in this case, it could be symptomatic and cause aesthetic problems. Composite resin restoration can be considered first as a minimally invasive method. Bonding to highly organic enamel is unfavorable, and dissolving the organic material improves bonding. In the present case, deproteinization technique was used to successfully restore the hypomineralized enamel.

# Abstract: 03. Minimally invasive treatment of invasive cervical root resorption: Surgical approach without the need of endodontic treatment

**Authors:** L. Schuster, T. Dammaschke

**Affiliations:** *Department of Periodontology and Operative Dentistry, University of Münster, Münster, Germany. laurentia.schuster@ukmuenster.de*

## Introduction

Invasive cervical root resorptions (ICR) can occur without causing clinical symptoms, frequently leading to a late discovery of the lesions. For correct diagnosis of ICR and treatment planning, three dimensional imaging is essential. Oftentimes, endodontic treatment of teeth with ICR is inevitable. This case report of a second right upper premolar (tooth 15) with ICR aims to illustrate a minimally invasive treatment protocol for preserving the tooth in function with the greatest possible preservation of tooth structure.

## Case Report

The 31-year-old female patient was referred to our clinic by a general practitioner, who had found the ICR lesion on tooth 15 on a bitewing image. The resorption lacuna was located in the distal part of the tooth. There were no clinically visible signs of ICR, tooth 15 was caries-free, the sensitivity of the tooth was positive and no tenderness to percussion was found. The aim of this case report is to illustrate a minimally invasive surgical approach for conservative treatment of ICR. Prior to treatment, a CBCT was performed. The CBCT showed a lesion 2Bp (Patel et al. 2017) with maximum extension of 5mmx4.3mmx2.7mm. A full-thickness papilla elevation flap was created under local anaesthesia to expose the resorption lacuna on tooth 15. The lacuna was completely excavated and connection to the pulp was visually and tactilely excluded. The resorption lacuna was carefully cleaned with 3% Natriumhypochlorite. Subsequently, a lining with Biodentine was placed, which covered the entire pulpaaxial and cervical area of the defect, and an adhesive composite resin restoration using a self-etch two-step universal bonding was placed. The flap was then repositioned and sutured with single Monocryl 6.0 button sutures. A postoperative X-ray was performed, which showed complete and sufficient filling of the resorption. 1.5 years after the treatment, the tooth is still clinically sound, symptom-free and vital, the proximal bone level is stable and the apical conditions are bland.

## Conclusions

In this case, the diagnosed ICR was an incidental finding on a bitewing radiograph. Even though caught early, the lesion had almost reached the pulp, yet conservative treatment under direct vision was sufficient. If caught early, ICR can be treated minimally invasive by surgically exposing the resorption lacuna and placing an adhesive composite restoration with a Biodentine lining, avoiding tooth structure loss or endodontic treatment.

# Abstract: 04. Direct injection technique for multiple anomalies: A minimally invasive, functional, and aesthetic approach

**Authors:** K. Demir, E. Yildiz

**Affiliations:** *Department of Restorative Dentistry, Institute of Graduate Studies in Health Sciences, Istanbul University, Istanbul, Turkey. demirkubra20@ogr.iu.edu.tr*

## Introduction

Using the direct injection technique and current advanced flowable composites, it is possible to treat diastemas, fractured teeth, hard tissue wear, dental anomalies, and teeth requiring esthetic corrections. In this case, tooth #12 exhibited microdontia, tooth #22 was congenitally missing, and tooth #23 showed transposition. Additionally, abrasion was observed in the persistent primary tooth #63. This case, presenting multiple dental anomalies simultaneously, aimed to achieve functional and esthetic restoration through modern minimally invasive techniques.

## Case Report

After determining the indications and treatment plan, a digital impression was taken to create the smile design. After 3D-printing the wax-up, a silicone index was obtained using clear polyvinyl siloxane material from the printed model. Following shade selection, the injection access points on the silicone index were verified with the teeth under rubber dam isolation. Only the cervical region of tooth #63, was minimally prepared and beveled. Adhesive procedures were performed by isolating the other teeth with polytetrafluoroethylene tape, applying orthophosphoric acid, and using a one-component light-cured universal adhesive. To eliminate any undercut areas of tooth #63, minimize polymerization shrinkage stress, prevent negative effects on the restoration's adhesion caused by forces acting on the cervical region, and camouflage discoloration, composite resin was applied in layers using free-hand modeling. The silicone index was positioned on the teeth, and flowable composite was injected through its access holes and polymerized. Occlusion was adjusted, finishing and polishing procedures were applied. As a result, tooth #12 and #23 were reshaped to resemble the upper lateral incisors, and tooth #63 was reshaped to resemble tooth #23.

## Conclusions

The direct injection technique, combined with advanced material technologies, provides an efficient, aesthetic, and functional solution for addressing dental anomalies. We believe this study will serve as a valuable reference for similar cases in the future. This study was independently funded by the authors.

# Abstract: 05. Deep margin elevation with resin composite and indirect restoration: A case report

**Authors:** M. Kim, S. Son, J. Park

**Affiliations:** *Department of Conservative Dentistry, School of Dentistry, Pusan National University, Yangsan, Korea. mayseven97@naver.com*

## Introduction

Subgingival margins are frequently encountered in clinical practice. Relocation of the subgingival proximal margin to supragingival level by using composite filling material could be a solution without surgical or orthodontic intervention. The technique of deep margin elevation offers the possibilities of performing a stepwise elevation of deep proximal cavities to create more favorable preparation margins for indirect restorations.

## Case Report

A 47-year-old male patient presented with secondary caries on the distal side of the maxillary right first premolar. The cavity was shown to extend sub-gingivally and beyond the CEJ, complicating isolation and further restorative procedure. Therefore, the treatment plan included a deep margin elevation (DME) with resin composite, followed by an indirect restoration. Palodent matrix system was used to elevate the sub-gingival margin to a supra-gingival position. Teflon tape was packed at the cervical part of the cavity to maintain adequate isolation. The process of DME involved first placing a thin layer of flow composite resin (Filtek Bulk Fill Flow, 3M ESPE), followed by a thicker layer of composite. The tooth was prepared for Class II resin inlay. After the proximal contact was verified, the enamel was selectively etched with 37% phosphoric acid, and a universal adhesive (Scotchbond Universal, 3M ESPE) was applied to the tooth. The resin inlay was cemented using RelyX U200 self-adhesive universal resin cement (3M ESPE). The patient was recalled after 3 months for evaluation of sensitivity, pain, and marginal adaptation and no such findings were seen.

## Conclusions

Elevating the proximal box to its supragingival level makes it easier to proceed the following procedures for indirect restorations. Clinical success of the restoration is directly related to the marginal seal and adaptation of the DME to the tooth. Clinical studies should evaluate long-term outcomes and optimal selection criteria to improve predictability.

# Abstract: 06. Restoration of a crown-root fractured tooth using orthodontic extrusion: A case report

**Authors:** J. Park, J. Park, S. Son

**Affiliations:** *Department of Conservative Dentistry, School of Dentistry, Pusan National University, Yangsan, Korea. parkjy8007@naver.com*

## Introduction

Restorative treatment of teeth with crown-root fractures is challenging due to the subgingival or crestal location of the defects. Orthodontic extrusion is a conservative procedure that allows tooth retention without loss of periodontal support.

## Case Report

A 13-year-old male visited the hospital with a fractured tooth caused by trauma from a baseball. Clinical examination revealed a crown-root fracture of the mandibular right canine. On the day of the first visit, pulpectomy was performed on the mandibular right canine. Subsequently, routine endodontic treatment was performed during the follow up visit. Following the endodontic treatment, a Stainless-Steel (SS) wire loop was attached from the mandibular right central incisor to the mandibular right first molar. A minitube was bonded to the mandibular right canine, and an elastic thread was inserted into the minitube and ligated to the SS wire. A month later, extrusion of the tooth was observed. Due to insufficient space between the minitube and SS wire, both the minitube and SS wire loop were debonded. Subsequently, minitubes were bonded from the mandibular right central incisor to the mandibular right second premolar, and a 012 Ni-Ti wire was inserted. One month later, sufficient extrusion was observed, and the minitubes were debonded. The teeth were stabilized for 12 weeks, using the buccal fixed retainer, to prevent relapse. A gingivectomy was also performed to excise the proliferated gingival tissue. Then, the tooth was restored using light-cure resin (Z350 A3, 3M ESPE and Gradia AO3, GC). Occlusion was checked and adjusted, followed by finishing and polishing. The patient was recalled after 1 month for evaluation of discomfort and no such finding was seen.

## Conclusions

Orthodontic extrusion is a safe and esthetic method that can preserve a tooth with crown-root fractures while reducing the possibility of adverse effects such as additional fracture or extraction.

# Abstract: 07. Composite resin restoration of crown-fractured anterior tooth due to trauma

**Authors:** S. Kim, S. Son, J. Park

**Affiliations:** *Department of Conservative Dentistry, School of Dentistry, Pusan National University, Yangsan, Korea. kimsy7801@naver.com*

## Introduction

The majority of dental trauma occurs in the maxillary anterior teeth. Crown fractures are the most common traumatic dental injury to permanent teeth, and the teeth most affected by trauma are the maxillary central incisors. In this case report, composite resin restoration of crown fractured anterior tooth was successfully performed esthetically.

## Case Report

Case report1: A 74-year-old female presented to the hospital after slip-down, with an avulsed #11 and fractured #11,21,22. Vitality of #21,22 were positive. #11 was immediately repositioned, and tooth splinting was performed. Root canal treatment of #11, direct resin restoration of #11,21,22 were planned. After adjacent teeth was isolated by Teflon tape, Mylar strip was manually adapted and palatal resin shell was formed with flowable resin. For esthetic demands of anterior restoration, incremental resin filling was performed using semi-translucent resin and opaque resin. Case report2: A 44-year-old female presented to the hospital after slip-down, with an alveolar fracture on the anterior maxilla, crown fractures of #11,12, and lateral luxation of #21,22. Vitality of #11,12 were positive. #21,22 were immediately repositioned, and rigid splinting was performed. Root canal treatment of #21,22, direct resin restoration of #11,12 were planned. The adjacent teeth were isolated with a Mylar strip, and the Mylar strip was manually adapted to form a palatal resin shell with flowable resin. For esthetic demands of anterior restoration, incremental resin filling was performed using semi-translucent resin and opaque resin. After finishing and polishing, esthetic results showed, and the patient was satisfied.

## Conclusions

For crown fractures located in the enamel or dentin without significant loss of tooth structure, composite resin can be a good alternative. Composite resin restoration is a highly conservative technique that restores the tooth with minimal reduction of healthy tooth structure. Incremental layering technique can provide restoration with morphology and shades similar to the adjacent dental structures.

# Abstract: 08. Current techniques in aesthetic and functional restorations – 6-month follow-up: two case reports

**Authors:** F.S. Aksoy, S. Yolcu, H. Kemaloglu

**Affiliations:** *Department of Restorative Dentistry, School of Dentistry, Ege University, İzmir, Turkey. f.siir@hotmail.com*

## Introduction

Defects in anterior teeth impact aesthetics and essential functions like chewing and speech. Minimally invasive and efficient restorative approaches are crucial. This study presents two cases where such methods addressed aesthetic and functional issues in anterior teeth.

## Case Report

Case 1: A 12-year-old male patient presented to our clinic due to dental trauma. Clinical examination revealed avulsion of tooth #11, an uncomplicated crown fracture on tooth #21, and initial carious lesions on anterior teeth. Tooth #11 was rehabilitated with an adhesive fiber bridge to serve as both an aesthetic and functional space maintainer until the patient reaches adulthood. The fractured tooth #21 was treated with aesthetic composite restoration, and the initial carious lesions were managed using the resin infiltration technique. The patient was monitored with periodic follow-ups for six months. Case 2: A 21-year-old female patient presented to our clinic with aesthetic concerns related to her anterior teeth. Clinical examination revealed a fracture in the incisal third of tooth #11 and hypomineralized areas in the incisal thirds of teeth #11 and #21. Vital bleaching was performed to mask the hypomineralized areas, followed by resin infiltration. The fractured tooth #11 was treated with aesthetic composite restoration. The patient was monitored with periodic follow-ups for six months.

## Conclusions

These case reports demonstrate the efficacy of minimally invasive and innovative restorative approaches in addressing aesthetic and functional challenges in anterior teeth. The combined use of adhesive fiber bridges, vital bleaching, composite restorations, and resin infiltration effectively met patients' aesthetic expectations while providing functional and biomechanical improvement. These treatment methods offer rapid and effective solutions, achieving both aesthetic and functional satisfaction, particularly in young patients.

# Abstract: 09. Reconstruction of severely crown-damaged teeth with 3D printed permanent resins: Case report

**Authors:** E. Karaaslan, A. Kaya, S. Aktug Karademir

**Affiliations:** *Department of Restorative Dentistry, Faculty of Dentistry, Ordu University, Altınordu/Ordu, Turkey. ekrsln.ek@gmail.com*

## Introduction

3D printed technology, which enables digital workflow in dentistry, has been integrated into clinical use with its low cost and ease of use. The aim of this study was to clinically evaluate the treatment of root canal-treated molars with extensive crown destruction (grade V) with 3D printed resin endocrown.

## Case Report

Female patients aged 18 and 28 years presented to our clinic for restoration of teeth number 16 and 46, respectively, without complaints of pain. Following the clinical and radiologic examination, the degree and direction of occlusal forces, the position of the tooth in the arch, and parafunctional habits were evaluated for the selection of restoration technique and materials. Treatment options were explained to the patient and with the patient's consent, endocrown treatment was decided. For more precise digital impressions, deep margin elevation was performed on the cavity margins below the gingival margin using resin flowable-composite (Filtek Bulk Fill Flowable Restorative, 3M ESPE, USA). Flat margin preparation was made for the endocrown, supported by the pulp chamber. Upon completion of the preparation, impressions were obtained using an intraoral scanner. The designed restoration was fabricated using 3D printer (Phrozen Sonic Mini 8K, Phrozen, Taiwan) and A2-resin (Saremco Print Crowntec, Saremco Dental AG, Switzerland). Post-polymerization and polishing were performed in accordance with the manufacturer's guidelines. Proximal contacts and occlusion were evaluated, and cemented to the isolated tooth using dual-cure resin cement (RelyX U200, 3M ESPE, Germany).

## Conclusions

At 1, 6 and 9-month follow-ups; the parameters of colour match, marginal discoloration, secondary caries, anatomical form, marginal adaptation, surface texture, and fracture were scored as Alpha (A) according to modified USPHS criteria. In severely damaged teeth, endocrown treatment may serve as a reliable alternative to post-core and crown restorations, preserving the strength of the remaining tooth structure.

# Abstract: 10. Esthetic smile rehabilitation with gingivectomy and laminate veneers: A case report

**Authors:** A.B. Karayel, A. Ozturk, M.B. Guncu

**Affiliations:** *Department of Restorative Dentistry, School of Dentistry, Hacettepe University, Ankara, Turkey. aslikarayelll96@gmail.com*

## Introduction

A 20-year-old male patient presented to the Department of Prosthodontics at Hacettepe University Faculty of Dentistry with esthetic concerns due to gaps between the upper anterior teeth. The patient had no complaints regarding tooth color. Intraoral examination revealed polydiastema in the maxillary anterior region and irregularities in the gingival zenith points. A treatment plan involving gingivectomy and laminate veneers was developed to restore an esthetic smile.

## Case Report

Digital impressions were obtained using a 3Shape IOS intraoral scanner and smile design photographs were captured. The collected data were evaluated and imported into EXOCAD software for case planning. A smile design was created for the maxillary anterior teeth, ensuring an 80% width-to-length ratio for the central incisors. A 3D-printed model was fabricated based on the design. Before initiating any invasive procedures, the design was transferred to the patient’s mouth using a silicone index and Protemp™ 4 (3M ESPE) bis-acrylic resin. The patient approved the design esthetically. Subsequently, the gingival zenith points were adjusted using a periodontal guide derived from the EXOCAD design, and soft tissue esthetics were achieved through gingivectomy. Thirty days post-surgery, following soft tissue healing, the approved design was re-applied to the patient’s mouth and Type 2 laminate veneer preparations were performed. Gingival tissues were retracted using Ultrapack retraction cords (Ultradent) and digital impressions of the prepared teeth were obtained. Pre-preparation photographs captured with a gray card were used to analyze the L*a*b* values for color selection. Based on this analysis, IPS E.max ceramic laminates were fabricated using CAD-CAM technology. The adaptation of the restorations was assessed using the try-in paste from the G-CEM Veneer Starter Kit (GC Corporation, Tokyo, Japan). After verifying the fit and esthetics, the restorations were cemented using G-CEM LinkForce™ light-cure adhesive resin cement (GC Corporation, Tokyo, Japan).

## Conclusions

At the first- and second-year follow-ups, the restorations demonstrated excellent esthetic and functional outcomes without any complications.

# Abstract: 11. Rehabilitation of a missing maxillary central incisor with implant-supported monolithic zirconia and advanced digital techniques

**Authors:** A. Ozturk, A.B. Karayel, M.B. Guncu

**Affiliations:** *Department of Restorative Dentistry, School of Dentistry, Hacettepe University, Ankara, Turkey. aslihanozturk10@gmail.com*

## Introduction

Soft tissue contouring is a necessary procedure for achieving pink and white aesthetics in the rehabilitation of anterior single-tooth deficiencies with implants. The accurate transfer of the soft tissue contour to the laboratory following the shaping process plays a significant role in achieving these aesthetic outcomes. This case report describes the fabrication of a screw-retained implant-supported restoration and the digital transfer of the soft tissue contour using a fully digital workflow.

## Material and Methods

A 50-year-old male patient was referred to the Department of Prosthodontics, Hacettepe University Faculty of Dentistry, with complaints of missing tooth #21 and esthetic concerns. Digital impressions were obtained using an intraoral scanner. To provide a preview of the final prosthesis and aid in implant surgery, a mirror image of tooth #11 was designed as a removable rochette using dental design program fabricated with a 3D printer and stained. The Rochette's position served as a reference for placing an implant in the #21 region. After a three-month healing period, a screw-retained temporary restoration was designed with software and fabricated from PMMA. The temporary prosthesis was used for four weeks to achieve an ideal soft tissue emergence profile. For the final prosthesis an additional digital impression of the temporary crown that was attached to the implant analog was obtained to precisely replicate the emergence profile. Dental photograph was taken with gray card and polarized filter to optimize the white balance of color and reflected light. The final prosthesis was milled from a monolithic zirconia block using the cut-back technique and layered with a novel glaze technique to achieve optimal esthetic harmony.

## Conclusions

Providing additional data to the technician during digital impression is a practical approach that enables predictable outcomes. Similarly, the use of a digital camera facilitates the transfer of color data to the laboratory.

# Abstract: 12. From orthodontics to aesthetic using hybrid injected-stratified technique

**Authors:** N. Pérez Candel, F. Muscillo, M. Carmona, V. Guzmán Coto, C. Martín Carreras-Presas, B. Baracco

**Affiliations:** *European University of Madrid, Madrid, Spain. natividadperezcandel@gmail.com*

## Introduction

Treatments which combine orthodontics and aesthetics are increasingly in demand at dental offices. In this case a 30-year-old patient came in to treat the increased overbite, decreased overjet and maxillary compression with invisible aligners with the purpose of distributing the space to be able to restore the teeth with two type of composite resin techniques.

## Case Report

Once the orthodontics was done, a teeth withening was performed using Norblanc Home (10%) prior to the final restorations. First, we did an injected technique by using Normofill Nanoceram Flow that emulates the dentin. Then, we performed a stratified technique to get the enamel with Normofill Nanoceram and the polish was made using the full kit of Normopull. Using both techniques we got better results.

## Conclusions

Bite and malpositions were resolved, always seeking as a result that the restorations would blend in to achieve harmony in the patient's smile.

# Abstract: 13. Diastema Closure and Anatomical Changes Using Universal Composites

**Authors:** V. Guzmán, F. Muscillo, M. Carmona, N. Pérez Candel, B. Baracco, C. Martín Carreras-Presas

**Affiliations:** *European University of Madrid, Madrid, Spain. valeguzman.97@gmail.com*

## Introduction

The patient, referred by orthodontics, attended the Dental Aesthetics Master’s program at the Universidad Europea de Madrid to improve her smile. Due to a Bolton discrepancy, it was decided to close the diastemas using non-invasive composite restorations and correct the triangular anatomy to rejuvenate the patient’s smile.

## Case Report

We selected the composite shade using the Botton Technique and a polarized photo with standarized values. We proceeded with absolute isolation, retracting the gums to properly give the anatomy. The surfaces of the teeth were roughened to promote a better adhesion. The enamel was etched with Normo-Etch 37% phosphoric acid for 15 seconds. Then we applied with a brush universal adhesive NormoBond. We used Normofill composite in shade A3 in the cervical areas and A2 in the incisal areas. Angular lines were defined to give the form using diamond burs. and last, polishing was performed using the Normon Polishing kit.

## Conclusions

The case was completed with a final polish two weeks later. We achievied seamless integration using only two composite shades, without the need of stratification and successfully closed the diastemas and corrected the triangular anatomy, resulting in a more harmonious and youthful smile. Universal composites are truly a great alternative for simplifying workflows in dental aesthetics while achieving high-quality results.

# Abstract: 14. Comprehensive esthetic restoration of maxillary central incisors with lithium disilicate ceramics: A case report

**Authors:** I. Sozen Yanik

**Affiliations:** *Department of Restorative Dentistry, School of Dentistry, Hacettepe University, Ankara, Turkey. iremsozen93@gmail.com*

## Introduction

Discoloration of teeth can occur as an undesirable outcome following root canal treatments. This problem is particularly significant when it involves anterior teeth, as it poses notable esthetic concerns. In such cases, prosthetic rehabilitation often becomes necessary to restore both function and appearance. This case aims to describe the rehabilitation of maxillary central incisors in a patient requiring improved esthetics using lithium disilicate ceramics.

## Case Report

A 35-year-old female patient presented to the clinic with an esthetic concern regarding her maxillary left central incisor, which also exhibited significant material loss. Clinical and radiographic examinations revealed that the tooth had undergone root canal treatment and showed discoloration. Additionally, the maxillary right central incisor displayed irregularities on the incisal edge of tooth. An initial impression was taken using polyvinyl siloxane (Elite HD+, Zhermack, Italy), and a wax-up was prepared, followed by a mock-up. The post space was prepared and a fiber post (Cytec Blanco 43.602, Hahnenkratt) were bonded with resin cement (RelyX U200, 3M), and a composite core (G-aenial A2, GC Corporation, Japan) was constructed, and the tooth was prepared for restoration. The maxillary right central incisor was also prepared for laminate veneer placement. Digital scanning of the patient was performed with TRIOS 5 intraoral scanner (3Shape, Denmark). Temporary restorations were placed to protect the prepared teeth. The digital design was reviewed on the CAD program and approved. Final restorations were fabricated using IPS e.max CAD blocks (Ivoclar Vivadent). The restorations were permanently cemented in accordance with the manufacturer's recommended instructions using G-CEM Veneer Light-Cure Resin Cement (GC, Japan).

## Conclusions

The esthetic and functional rehabilitation of the maxillary central incisors was successfully achieved using lithium disilicate ceramic restorations. This case highlights the importance of a comprehensive treatment approach. Integrating precise digital planning with advanced restorative techniques to achieve the desired esthetic outcomes for the patient.

# Abstract: 15. Assessment of two software applications as a potential digital tool in tooth shade selection: A case report

**Authors:** H. Gokce, A. Ozturk, M.B. Guncu

**Affiliations:** *Department of Restorative Dentistry, School of Dentistry, Hacettepe University, Ankara, Turkey. handegokce@windowslive.com*

## Introduction

In dental esthetics, the primary objective is to replicate the characteristics of adjacent teeth and achieve a natural appearance. Various methods for color selection have been proposed. In recent years, color determination through calibrated photographs has been reported as a practical approach. This case report aims to illustrate the process of color selection based on photographic analysis of a severely compromised and discolored tooth.

## Case Report

A 53-year-old female patient was referred to the department of prosthodontics with complaints of a fractured tooth and aesthetic concerns. Due to the discoloration on the left lateral incisor, devital bleaching was performed. After this treatment, the patient reported that the tooth had fractured. To enhance the durability of the remaining dental tissue, a fiber-reinforced composite filling (EverX Flow; GC) was performed. Tooth preparation was completed, digital impression was taken using intraoral scanner (TRIOS 5; 3Shape). Shade tabs were selected from each value group of the Vita Classical Shade Guide (Vita Zahnfabrik) that were closest in color to the tooth requiring a shade match. A photograph was taken using a camera and a twin flash with a gray reference card which helps standardize digital images irrespective of clinic lighting by using a white balance calibration. The captured photo was saved and imported into a photo editing smartphone application (Snapseed; Google LLC) and photo editing software (Adobe Photoshop). Shade selection was performed separately on both applications. The technician took photographs using the same gray card during the fabrication process and performed color verification. It was observed that both methods resulted in similar outcomes for the crowns.

## Conclusions

The use of digital methods in color evaluation and their application in clinical practice yields more predictable results. It is possible to make color selection decisions based on images obtained with a camera using simple and inexpensive software.

# Abstract: 16. Aesthetic rehabilitation of a geriatric patient with dental bleaching and composite resin restoration

**Authors:** N.N. Karamanci, C. Peskersoy

**Affiliations:** *Department of Restorative Dentistry, School of Dentistry, Ege University, İzmir, Turkey. nimetnurkaramanci@gmail.com*

## Introduction

A 65-year-old female patient applied to our clinic with aesthetic complaints. Intraoral examination revealed tooth discoloration and polydiastema. A treatment plan involving in office dental bleaching and diastema closure with composite resins was established.

## Case Report

Initially, the tooth color was determined as A4 using a Vita shade guide. Soft tissues were protected by applying an intraoral retractor and a gingival barrier. An in-office bleaching procedure was performed using a 40% hydrogen peroxide agent (Opalescence Boost, Ultradent, USA), applied three times for 20 minutes. Following whitening, the tooth color was reassessed as A2. The patient was advised to avoid chromogenic foods and beverages to maintain color stability for two weeks. The patient came back 2 weeks later for restorative procedures. The composite resin shade was selected using the button technique. Isolation was achieved with a rubber dam (Dental Dams, Royal Shield, Malaysia). A diamond bur was used to slightly abrade the vestibular and proximal surfaces. A teflon band was placed to protect adjacent teeth. A 37% orthophosphoric acid gel (Condoc 37, FGM, Brazil) was applied for 30 seconds, followed by rinsing and drying. A universal adhesive (Solare Universal Bond, GC, Japan) was applied and light-cured for 20 seconds. Diastema closure was performed using free-hand layering technique with a universal composite resin (A2; G-enial Accord, GC). Anatomical contouring was completed with finishing burs. Final polishing was performed using polishing discs (Polishing Discs, TOR VM, Russia) and polishing wheels (Twist Dia, Kuraray, Japan).

## Conclusions

With dental bleaching and direct composite resin restorations, a better aesthetic appearance for the patient was achieved in two sessions. After treatment, the patient's self-confidence and quality of life increased dramatically. Additionally, these minimal invasive treatment options were conservative with minimal tooth structure loss. The authors received no funding from any individual or institution.

# Abstract: 17. Rehabilitation of bilateral congenital maxillary lateral incisor agenesis with temporary composite restorations

**Authors:** M. Öz Şengül, B. Balikçi, A. Demirbaş

**Affiliations:** *Department of Restorative Dentistry, School of Dentistry, Ege University, İzmir, Turkey. dtmerveoz@gmail.com*

## Introduction

An 18-year-old male patient presented with bilateral maxillary lateral incisor agenesis to the Restorative Dentistry Clinic of Ege University. Intraoral examination revealed no other missing teeth or restorative needs. The patient, undergoing orthodontic treatment with future implant placement planned, required a temporary restoration to improve smile aesthetics. Due to ongoing orthodontic treatment, adhesive bridge application was not feasible.

## Case Report

Following consultation with the orthodontist, temporary lateral incisors were fabricated using composite resin and placed in the tooth spaces with brackets. As the patient’s orthodontic treatment involved rectangular wires, rotational movement of the temporary teeth was deemed unlikely. An impression was taken using silicone material (Zetaplus, Zhermack, Italy). Prefabricated acrylic teeth were used as a reference to create a silicone index. Composite resin (G-Aenial Achord, GC Dental, Japan) was layered into the index and light-cured. Primary anatomy was refined with abrasive disks (Tor, VM, Russia), and an ovate pontic form was created for aesthetics and cleanability. Finishing and polishing were completed with spiral disks (Twist Dia, Kuraray, Germany). Brackets were bonded to the composite crowns by the orthodontist, and the temporary teeth were secured with ligatures. Lateral and protrusive contacts were checked, and patient instructions were provided. Follow-up visits revealed high patient satisfaction.

## Conclusions

Temporary composite teeth are a cost-effective and adaptable solution for patients with anterior tooth agenesis undergoing orthodontic treatment. They provide satisfactory results until permanent restorations are placed. These restorations can also be retained and used with orthodontic retainers in cases where implants are anticipated post-orthodontic treatment. The authors declare no funding from any individual or institution.

# Abstract: 18. Aesthetic rehabilitation of a fused tooth and carious maxillary teeth

**Authors:** I. Yumak, L.S. Turkun

**Affiliations:** *Department of Restorative Dentistry, School of Dentistry, Ege University, İzmir, Turkey. ilaydayumak95@gmail.com*

## Introduction

This case report presents the aesthetic rehabilitation of a fused tooth (#11) and carious maxillary teeth in a 15-year-old male patient prior to orthodontic treatment.

## Case Report

A 15-year-old male patient was referred to the Department of Restorative Dentistry at Ege University School of Dentistry in January 2025 by the Department of Orthodontics for pre-orthodontic rehabilitations. Clinical examination revealed fusion in tooth #11 with an enamel tubercule on its buccal surface, preventing bracket placement. Tooth #12 was completely palatally positioned, and carious lesions were detected in teeth #13, #21, #22, and #23. The patient received first a periodontal treatment due to his gingival bleeding. Restorative procedures were then initiated. Due to crowding, rubberdam placement was not possible, so isolation was achieved using OptraGate (Ivoclar, Liechtenstein). All teeth were etched with orthophosphoric acid (Condac 37, FGM, Brazil) for 30 seconds, rinsed for 30 seconds, and dried. A universal adhesive (G-Premio Bond, GC, Japan) was applied for 20 seconds and light-cured (Woodpecker, Guilin Woodpecker Medical Instrument, Switzerland). The teeth were restored with a nanohybrid composite resin (G-aenial Achord, A2, GC, Japan). Tooth #11 had an increased mesiodistal width, so both central and lateral incisor’s morphology was created on its buccal surface. To mask the crowding of tooth #21, stripping was performed on its distal enamel. Tooth #22, being more palatally positioned, was restored with composite resin on its labial surface to bring it into a more vestibular alignment. Finishing and polishing were performed using finishing discs and polishing wheels (Twist Dia, Kuraray, Japan). The patient was recalled for a control session.

## Conclusions

During the follow-up session one week later, necessary modifications, contouring, and additional polishing were performed. The patient was then referred to the Department of Orthodontics for further treatment. Throughout that treatment process, the aesthetic restorations will be modified according to tooth movements.

# Abstract: 19. Minimally invasive management of white spot lesions in patient

**Authors:** T. Hasmaden, E.N. Karadumanli, G.I. Erisken, A. Demirbas

**Affiliations:** *Department of Restorative Dentistry, School of Dentistry, Ege University, İzmir, Turkey. tugcehasmaden@gmail.com*

## Introduction

White spot lesions and cavitated enamel defects are common aesthetic and structural issues in adolescent patients, often caused by enamel demineralization or hypomineralization. This case report highlights the comprehensive management of a patient presenting with White spot lesions and cavitated enamel defects using a minimally invasive treatment protocol to restore aesthetics and function.

## Case Report

A patient presented with prominent White spot lesions on anterior teeth and localized cavitated enamel defects. The treatment plan included: Office Bleaching: Performed with 35% hydrogen peroxide (FGM Whiteness HP, Brazil) to improve the overall tooth shade and reduce the contrast between the lesions and the surrounding enamel. Home Bleaching: A supervised bleaching protocol with 16% carbamide peroxide gel (FGM Whiteness Perfect, Brazil) was prescribed for 7-14 days to enhance and maintain the results. ICON Resin Infiltration: Applied to white spot lesions to camouflage the lesions by ICON (DMG, Japan) infiltrating resin into the porous enamel and blending them with surrounding tooth structure. Direct Composite Restorations: Cavitated enamel defects were restored with nanohybrid composite (Estelite Asteria, Tokuyama, Japan) material using adhesive techniques, ensuring optimal shape, color, and function. Clinical outcomes included successful camouflage of white spot lesions, restoration of cavitated defects, and a natural, uniform tooth shade. Both the patient and their parents expressed high satisfaction with the treatment results.

## Conclusions

This case demonstrates the effectiveness of a minimally invasive, stepwise approach combining bleaching, ICON resin infiltration, and direct composite restorations in managing white spot lesions and cavitated enamel defects in adolescents. This protocol achieved excellent aesthetic and functional outcomes while preserving tooth structure. The authors acknowledge the support of the patient and their family during the treatment process. No external funding was received for this study.

# Abstract: 20. Enamel surface hypoplasia treated with a combination of ICON infiltrant and direct composite resin restoration

**Authors:** S. Yalniz, L.S. Turkun

**Affiliations:** *Department of Restorative Dentistry, School of Dentistry, Ege University, İzmir, Turkey. selinbuyukyilmaz@gmail.com*

## Introduction

This case report describes the treatment of enamel hypoplasia in teeth 11-21-31-42 with a resin infiltration technique combined with direct composite resins.

## Case Report

A 21-year-old male patient applied to the Department of Restorative Dentistry in January 2025 for esthetic complains about the appearance of his upper and lower anterior teeth. The patient had no systemic disease. At the intra-oral examination, it was observed that he had enamel surface hypoplasia on the incisal edge of teeth 11-21-31 and 42. The resin infiltration technique (DMG Icon, Hamburg, Germany) was proposed to the patient. Enamel surfaces were roughened under rubber- dam isolation.15% HCl acid gel (Icon-Etch) was applied to the lesions for 2 minutes. Then the acid was rinsed for 30 seconds. As a next step, ethanol (Icon- Dry) was applied for 30 seconds and dried. This process was repeated for consecutive 4 times. Then resin infiltrant (Icon-Infiltrant) was applied to the surfaces for 3 minutes and polymerized for 40 seconds. The resin was applied again for a minute and polymerized for another 40 seconds. The hypoplasia of teeth 11 and 21 decreased however, the desired esthetic appearance could not be achieved. It was then decided to use direct composite resins for restorative purposes. The remaining hypoplasia areas were abraded, etched with 37% orthophosphoric acid for 30 seconds, rinsed and dried. A universal adhesive system GC Premio Bond (GC, Japan) was applied by a rubbing motion for 20 seconds and light cured for 20 seconds (D-light Pro, GC). The teeth were restored minimal invasively with G-aenial Universal Injectable flowable composite resin (A2 shade). Finishing and polishing were performed with Clearfil Twist Dia (Kuraray, Japan) polishing wheels.

## Conclusions

The patient was satisfied with the final esthetic result achieved minimal invasively and in a single appointment.

# Abstract: 21. Comprehensive oral rehabilitation in a patient with severe tooth wear and vertical dimension alteration

**Authors:** C. González-Buendía, O. González Castro, I. González, L. Ceballos, V. Fuentes

**Affiliations:** *Master’s Degree in Restorative Dentistry, Aesthetics and Endodontics, Faculty of Health Sciences, Rey Juan Carlos University, Madrid, Spain. cruzgbuendia@gmail.com*

## Introduction

A 66-year-old male patient was referred following implant surgery for evaluation of a full-mouth prosthetic rehabilitation. He presented severe tooth wear of multifactorial etiology (bruxism, malocclusion and carbonated beverages), altered vertical dimension, and missing teeth where implants were placed. The rehabilitation treatment aimed at restoring and establishing a stable occlusion for the patient with a minimal intervention approach.

## Case Report

The case was planned using photographs, digital smile design, and diagnostic digital wax-up with subsequent mock-up. Initially, the worn maxillary teeth were directly restored with composite resin (Spectra ST Flow, Spectra ST LV, Dentsply Sirona). Previously to implants rehabilitation, zirconia single crowns (Monolithic Multilayer Zirconia, Nacera) were placed on teeth 1.4 to 2.4, a fixed zirconia bridge on teeth 1.5 to 1.7 (Monolithic Multilayer Zirconia, Nacera) and teeth 2.5 and 2.6 were restored with lithium disilicate veneerlays (IPS Emax CAD, Ivoclar Vivadent). Meanwhile, an at-home bleaching treatment (Polanight CP 16%, SDI) of the mandibular teeth was carried out for 4 weeks, after sealing non-carious cervical lesions in advance (Riva light cure, SDI). Mandibular anterior teeth were restored with the injection moulding techique (G-aenial Universal Inyectable B1, GC) with a transparent perforated silicone index (Exaclear, GC) and a total-etch technique with a universal adhesive (G Premio Bond, GC). Mandibular premolars were restored using the same silicone index with a stamp technique applying preheated composite resin (Filtek Supreme XTE B1, 3M ESPE). Finally, zirconia-based implant-supported single crowns were screwed to replace the missing teeth. Full-mouth rehabilitation was successfully achieved by re-establishing the patient’s occlusal vertical dimension and restoring the aesthetics and functionality.

## Conclusions

A multidisciplinary treatment plan is essential for improving prognosis and patient satisfaction when combining implant-supported and tooth-supported restorations. A variety of Material and techniques may be required for complex full-mouth rehabilitations.

# Abstract: 22. Diastema management in a case with Bolton discrepancy: Aesthetic approach with a monochromatic composite resin

**Authors:** P.L. Carrión Borreguero, C. González Font, F. Muscillo, M. González Gancio, M. Carmona, B. Baracco

**Affiliations:** *European University of Madrid, Madrid, Spain. pauladelluch@gmail.com*

## Introduction

Bolton discrepancies, characterized by disproportions in tooth size, require an interdisciplinary approach between orthodontics and aesthetic dentistry. In cases where orthodontic treatment alone is insufficient or unfeasible, composite resins provide a conservative and highly aesthetic alternative for diastema closure. Composites allow for minimally invasive restorations, preserving dental structure and achieving harmonious results. However, the reduced thickness of these restorations can make the incremental technique difficult. Monochromatic universal composites can be an effective alternative for a successful aesthetic result.

## Case Report

This report presents the case of a 27-year-old female patient who attended the Master Program in Aesthetic Dentistry at the Universidad Europea (Madrid) seeking to improve the appearance of her anterior teeth. A Bolton discrepancy was diagnosed, which could not be resolved exclusively by previous orthodontic treatment. Therefore, diastema closure with composite resins was indicated for the segments from 1.3 to 2.3 and from 3.3 to 4.3. A minimally invasive preparation was performed, using rubber dam isolation to optimize the adhesion procedure and prevent contamination. The restoration was carried out using a multimode adhesive (Scotchbond Universal, Solventum) and a “single shade” or “monochromatic” universal composite: Omnichroma (Tokuyama). This type of composite can mimic the tooth structure by means of a “chameleon effect”, which simplifies the technique and shortens the clinical working time.

## Conclusions

Treatment resulted in a satisfactory aesthetic outcome, preserving dental structure and achieving a harmonious smile. This case reinforces the feasibility of using composite resins for managing Bolton discrepancies. The advantages associated with the color mimetic potential of universal single shade composites must be taken into account for this type of procedures.

# Abstract: 23. Amelogenesis imperfecta: conservative approach using microabrasion and direct composite veneers

**Authors:** M. Flores Sanhueza, A. Dris, F. Muscillo, S. Abularach, M. Carmona, C. Martín Carreras-Presas

**Affiliations:** *European University of Madrid, Madrid, Spain. matflorsan@gmail.com*

## Introduction

Amelogenesis imperfecta (AI) is a genetic anomaly that affects enamel development, altering its structure, color, and strength. It compromises dental aesthetics and impacts the masticatory function and patient's quality of life. Among its different subtypes, hypoplastic AI is characterized by thin, rough enamel with irregularities on the dental surface. Clinical management of these cases requires a comprehensive approach that combines conservative techniques and high-aesthetic Material to restore smile harmony without compromising the remaining dental structure.

## Case Report

A 25-year-old female patient sought consultation due to concerns about the appearance of her smile, expressing insecurity regarding the aesthetics of her teeth. Extraoral evaluation showed a harmonious facial symmetry, while intraoral analysis revealed hypoplastic enamel with a rough texture and an opaque color, with no presence of caries or periodontal alterations. To ensure a predictable outcome aligned with the patient's expectations, a structured treatment protocol was followed: 1) Mock-up for aesthetic and occlusal validation: diagnostic wax-up was performed to evaluate the integration of the design, 2) Color selection: a direct chromatic mapping technique ensured an integrated appearance of the restoration, 3) Enamel preparation: A superficial microabrasion was performed to enhance composite adhesion (Scotchbond Universal, Solventum) and homogenize the dental tissue, 4) Fabrication of direct composite veneers: A silicone key was used to guide the layering process, 5) Layering technique: Filtek Supreme XTE (Solventum) was used for the palatal shell, Empress Direct White (Ivoclar Vivadent) for the incisal halo, Enamel Plus HRI (Micerium) for the UD1 dentin layer and Esthelite WE (Tokuyama) for enamel outer layer.

## Conclusions

Direct composite veneers successfully restored the patient´s aesthetics and functionality, positively impacting her confidence. The combination of conservative techniques with high-aesthetic Material represents an effective alternative for managing AI, prioritizing the preservation of dental structure and the customization of treatment according to the patient’s individual needs.

# Abstract: 24. Using composites with different opacities to properly mask a dark dental substrate

**Authors:** M. González Gancio, M. Carmona, F. Muscillo, P.L. Carrión, B. Baracco, C. Martín Carreras-Presas

**Affiliations:** *European University of Madrid, Madrid, Spain. 22488611@live.uem.es*

## Introduction

Restoring teeth with dark substrates presents a significant challenge in aesthetic dentistry. The strategic use of composite resins with varying opacities is crucial to effectively mask dark areas while maintaining a natural and functional appearance. This case highlights the importance of selecting appropriate Material and layering techniques to achieve optimal aesthetic results in anterior restorations.

## Case Report

A 35-year-old male presented with a chief complaint of a darkened upper right central incisor (tooth 11). Pulp necrosis was diagnosed, with no significant pain reported. Diagnostic imaging confirmed the absence of acute infection. The treatment plan included root canal treatment and subsequent restoration of the tooth. A composite shade was selected using the “ball” color matching technique, enhanced by a polarized photograph for accurate shade determination. The procedure began with absolute isolation and thorough substrate preparation. Enamel was etched with 37% phosphoric acid, followed by the application of adhesive (Excite F, Ivoclar Vivadent). An opaquer was applied to mask the dark substrate before layering the composite. The palatal shell was constructed using the composite Harmonize Blu (Kerr). A more opaque dentin shade (Filtek Supreme XTE, A1, Solventum) was applied, followed by Empress Direct Transopal (Ivoclar Vivadent) to enhance translucency between the mamelons. Finally, the restoration was completed with a final layer of “enamel” composite (Harmonize Blu). The restoration thickness was carefully controlled at each stage, and the final surface was polished with Ivoclar Ultra Gloss.

## Conclusions

The use of composite resins with different opacities is an effective method for masking dark substrates. This case demonstrates how careful material selection and precise technique lead to a functional and aesthetically pleasing restoration. The layering technique ensured excellent color matching and translucency, mimicking the appearance of natural teeth. Moreover, the polished surface could achieve a high gloss, replicating the natural texture of enamel.

# Abstract: 25. Esthetic smile rehabilitation with orthodontic treatment and maxillary laterals ceramic veneers: A case report

**Authors:** G. Aksoz

**Affiliations:** *Department of Orthodontics, School of Dentistry, Hacettepe University, Ankara, Turkey. gedizaksoz@gmail.com*

## Introduction

The aim of this case report is to present the anterior smile aesthetic improvement of an open bite patient with congenitally missing lateral, deviated upper dental midline, reduced incisor exposure in the smile, and emphasize the importance of orthodontic treatment in conservative dentistry.

## Case Report

A 17-year-old female patient applied to the Prosthodontics Department with the complaint of diastemas in the upper anterior region and unsatisfied smile aesthetics. The patient was recommended treatment with full-mount maxillary rehabilitation. The patient seeking more conservative treatment was referred to the Orthodontics Department. Clinical and radiographic examinations showed that the patient had class 2 malocclusion, increased overjet and open bite, congenitally missing left lateral, peg-shaped upper right lateral, nonconsonant smile arch, and decreased display of the incisors in the smile. The upper dental midline was deviated 5 mm to the left, a total of 10 mm diastemas were existed between the upper anterior teeth, and the space of 2.2 was closed. With orthodontic treatment, class 2 malocclusion and overjet were corrected, the upper dental midline was shifted to the right, and the space of 2.2 was prepared. After orthodontic treatment, a dental implant with a diameter of 2.9 mm was placed at the site of 2.2. Feldspatic-ceramic-veneered-zirconia crown was cemented with zinc-phosphate cement. The peg-shaped lateral was restored with a zirconia ceramic veneer to achieve a symmetrical appearance with 2.2.

## Results

After multidisciplinary treatment, dental midlines were aligned with the facial midline. Maxillary incisor exposure was increased. Ideal dental relationships, consonant smile arch, and aesthetic smile were achieved.

## Conclusions

In this patient who was recommended treatment with maxillary full-mouth rehabilitation, an aesthetic smile was achieved with only maxillary lateral restorations with orthodontic treatment. Orthodontic treatment should be taken into consideration in the conservative approach, especially in young patients.

# Abstract: 26. Esthetic and functional rehabilitation of a root canal-treated maxillary incisor with implant and advanced restorative materials

**Authors:** I.G. Uluc

**Affiliations:** *Flydent Dental Clinic, Budapest, Hungary. okceuluc@hotmail.com*

## Introduction

Root canal-treated teeth often lose significant structure, compromising function and esthetics. Even with a crown, support may be inadequate. In such cases, dental implants and advanced restorative Material provide predictable solutions for optimal function and esthetics. The aim of this case report is to present the rehabilitation of a root canal-treated maxillary right central incisor that failed functionally and esthetically, requiring replacement with a dental implant and restorative restorations for optimal esthetics and soft tissue integration.

## Case Report

A 27-year-old female patient presented with an esthetic concern regarding her maxillary right central incisor. Clinical and radiographic examinations revealed extensive structural loss following root canal treatment, causing repeated decementation of the crown due to esthetic and functional failure. After a thorough consultation, the decision was made to extract the compromised tooth and replace it with a dental implant and esthetic restoration. While waiting for implant osseointegration, a composite resin-based Maryland bridge was retained using adjacent lateral and central teeth. Once osseointegration was achieved, a provisional crown on a Ti-base abutment was adjusted over three weeks to achieve the desired emergence profile. Once optimal soft tissue esthetics were obtained, a customized impression coping technique ensured accurate emergence profile replication. A zirconia-core was fabricated, and a lithium disilicate ceramic crown was manufactured over that zirconia-core. The zirconia-core was cemented extraorally onto the Ti-base abutment, then torqued onto the implant. Finally, the lithium disilicate ceramic crown was cemented onto the zirconia-core intraorally using resin cement.

## Conclusions

The functional and esthetic restoration of the maxillary central incisor was successfully achieved using a lithium disilicate ceramic crown, a zirconia-core, and a Ti-base abutment. This case underscores the importance of a comprehensive treatment strategy, incorporating precise soft tissue management, an optimized emergence profile, and advanced restorative Material and techniques to achieve superior esthetic outcomes for the patient.

# Abstract: 01. Management of hypomineralization - demineralization lesions post-orthodontic treatment with resin infiltration: 3 case reports

**Authors:** E.N. Karadumanlı, G.I. Erişken, T. Hasmaden, A. Demirbaş

**Affiliations:** *School of Dentistry, Ege University, İzmir, Turkey. edakmanli@gmail.com*

## Introduction

Objective: The aim of this case report is to evaluate the efficacy of the minimally invasive resin infiltration technique in the treatment of developmental hypomineralization and demineralized lesions after orthodontic treatment and to compare the aesthetic results of three different cases.

## Case Report

Case 1: A 23-year-old male patient presented to our clinic with the complaint of discoloration of his anterior teeth. Radiological evaluation revealed no pathology; however, intraoral examination revealed demineralization and opaque lesions in the gingival third of teeth numbered 13, 12, 11, 21, 22 and 23. The lesions were treated with resin infiltration technique applied in accordance with the manufacturer's instructions after polishing. In addition, the minimal fracture in tooth number 11 was restored with resin composite material. Case 2: A 28-year-old male patient was admitted to our clinic and no pathology was detected in the radiological evaluation; however, opaque lesions due to developmental hypomineralization were detected in the incisal third of teeth number 13, 12, 11, 21, 22 and 23. After polishing, the lesions were treated with resin infiltration technique applied according to the manufacturer's instructions; however, hypomineralization lesions in the incisal areas could not be completely treated. Case 3: A nineteen-year-old female patient was admitted to our clinic with the complaint of hypomineralization and opaque lesions on tooth number 13. The lesions were successfully treated with the resin infiltration technique applied in accordance with the manufacturer's instructions after polishing.

## Conclusions

These cases demonstrate that the resin infiltration technique is an effective method for the treatment of enamel demineralization after orthodontic treatment. However, the effectiveness of this method may be limited in developmental hypomineralization lesions in incisal areas. More comprehensive clinical studies are needed to evaluate the long-term efficacy of resin infiltration and its applicability to other enamel lesions.

# Abstract: 02. Acute treatment of highly caries-active 15-year-old female patient

**Authors:** R. Schmidtke, S. Paris

**Affiliations:** *Department of Operative, Preventive and Pediatric Dentistry, Charité – Universitätsmedizin Berlin, Berlin, Germany. rebekka.schmidtke@charite.de*

## Introduction

This case report documents the treatment of a fifteen-year-old female patient who presented with multiple carious lesions and inadequate oral hygiene. The patient had a history of resection of an embryonal rhabdomyosarcoma in the oropharynx with subsequent chemotherapy and proton therapy over the course of six years without dental care during this period. In the initial appointment oral hygiene was insufficient but hyposalivation could not be observed. The root growth and eruption of the second molars on the right side was not completed. The carious destruction was particularly severe in the anterior region and on three first molars.

## Case Report

Initially, the patient was instructed to improve oral hygiene was but showed only limited adherence. Therefore, the SMART-Hall technique, an established treatment concept in pediatric dentistry was employed: The caries lesions in the first molars were arrested with silver diamine fluoride (Riva-Star) and subsequently restored prefabricated stainless steel crowns (3 M Espe). In consecutive appointments under nitrous oxide sedation, carious lesions in premolars and anterior teeth were semi-temporarily restored with strip crowns (Frasaco) and composite restorations (Tetric Evo Ceram, Ivoclar). The patient responded well to the initial treatment and, due to the improvement in aesthetics, showed increased motivation for oral hygiene. In subsequent steps, extraction in case of still missing eruption of the retained molars, and clarification of the orthodontic options for the alignment of the wisdom teeth is planned. As a definitive restoration was not initially possible, the planning for the definitive crowns will follow.

## Conclusions

This case demonstrates the successful use of an established pediatric dentistry concept in a medically complex adolescent patient, to manage severe carious lesions with a focus on stabilizing oral health and improving patient compliance before definitive treatment.

# Abstract: 01. The impact of maturation and protective coating on the discoloration of the restorative materials

**Authors:** A. Ivanišević, K. Vresk, J. Matijević, I. Šalinović, I. Miletić, E. Klarić

**Affiliations:** *School of Dental Medicine, University of Zagreb, Zagreb, Croatia. aivanisevic@sfzg.hr*

## Aim

The aim of the study was to determine the impact of the degree of maturation and protective coating on the discoloration of glass ionomer-based Material after cyclic exposure to green tea.

## Material and Methods

The Material tested were high-viscosity glass ionomer Ketac Universal Aplicap (Ketac) and glass-hybrid material Equia Forte HT Fil (Equia). Samples were divided into eight experimental groups — each material in both non-matured and matured forms, with and without the protective coating: 1. Ketac; 2. Ketac Coat: 3. Equia; 4. Equia Coat; 5. KetacMatured; 6. KetacMatured Coat; 7. EquiaMatured; 8. EquiaMatured Coat. L* a* b* components within the CIELAB system were recorded using a spectrophotometer, before and after cyclic exposure to green tea, 3 times a day for 10 minutes, for 14 days. Changes in color (ΔE) were calculated for each sample. Differences between groups were tested using one-way ANOVA and post hoc Scheffe at the significance level α=0.05.

## Results

In Ketac groups, color change was the smallest in KetacMatured (ΔE=2.108±0.336), and the highest in Ketac (ΔE=2.7±0.666), but the difference between the groups was not significant (p>0.05). In Equia groups ΔE was the smallest in EquiaMatured Coat (ΔE=1.175±0.272) and it was significantly lower than in all other Equia groups (p<0.05). Color change was highest in Equia (ΔE=4.887±1.119) and EquiaMatured (ΔE=4.835±0.817) and it was significantly higher than in all other Ketac and Equia groups (p>0.05).

## Conclusions

Ketac groups with matured and coated samples exhibited less color change, but the effect of maturation and coating was not significant. In glass hybrid material, matured and coated samples resulted in superior color stability. The effect of coating on color stability in Equia was significant, but the effect of maturation was not significant.

# Abstract: 02. Effects of different surface treatments on stainability of additive manufactured definitive crown resins

**Authors:** E. Sonkaya, Z. Süsgün Yıldırım

**Affiliations:** *Department of Restorative Dentistry, Faculty of Dentistry, Cukurova University, Adana, Turkey. ezgisonki@gmail.com*

## Aim

This study aimed to determine the surface polishing effect on discoloration properties of three-dimensional printed dental definitive resin.

## Material and Methods

Formlabs permanent crown resins were used to fabricate 40 rectangular-shaped specimens (14 × 12 × 1 mm) (n=40). After baseline color (L, a, b) measurements, specimens were divided into four groups based on the polishing technique; samples with untreated surfaces, glazed (Optiglaze, GC) surfaces, sand-glazed surfaces and pumice alcohol surfaces (n=10). After polishing, specimens were subjected to 12 days of coffee immersion. Color coordinate measurements were performed again. Color difference (ΔE00) was calculated. The Shapiro-Wilk test was used to assess the normality of the distribution in the groups. For parameters that did not follow a normal distribution, the Kruskal-Wallis test was applied. To detect differences between groups, the Post Hoc Dunn's test was employed. A statistical significance level of p<0.05 was considered for all tests.

## Results

There was a significant difference in the mean ΔE00 levels among the four groups (p<0.001). When examining the differences between the groups, it was observed that the pumice group exhibited less discoloration compared to all other groups. Although there was no statistical difference between the sandblasting Optiglaze and pumice groups, both exhibited significantly less discoloration compared to the control and Optiglaze groups.

## Conclusions

The surface glazing technique significantly influences the stainability of 3D-printed dental permanent resins. Among the tested groups, the pumice-polished surfaces exhibited the least discoloration. This finding highlights the importance of selecting appropriate surface treatment to enhance the aesthetic longevity of 3D-printed dental materials.

# Abstract: 03. Adesensitizer as a remineralization agent

**Authors:** I. Gonullu, E. Akay, H. Kemaloglu, M. Turkun

**Affiliations:** *School of Dentistry, Ege University, İzmir, Turkey. idilgonullu@icloud.com*

## Aim

This study aims to comparatively examine the remineralizing effects of varying concentrations of nano-hydroxyapatite combined with potassium nitrate on enamel and dentin surfaces.

## Material and Methods

Twenty-four samples were prepared for our investigation. After being embedded in acrylic, the samples were randomly divided into four groups, and dentin surfaces were obtained by randomly selecting samples for the dentin subgroups. Six dentin samples were treated with a 2.5% nano-hydroxyapatite agent, while six other samples were treated with a 5% nano-hydroxyapatite agent. In a similar manner, six enamel samples were subjected to treatment with a 2.5% nano-hydroxyapatite agent, while another six enamel samples received treatment with a 5% nano-hydroxyapatite agent. The samples were subjected to demineralization with a demineralization solution for 96 hours before the application of remineralization agents. The agents were administered bi-daily for a period of 7 days. Subsequent to the agents' removal, the samples were preserved in an artificial saliva solution until the next procedure. The surface hardness of the samples was evaluated three times: initially, after demineralization, and post-remineralization, utilizing Vickers microhardness testing.

## Results

A significant increase in microhardness values was observed in all test groups (p<0.05) when measurements taken after the application of remineralization agents were compared to those after demineralization. No statistically significant difference was observed in the microhardness increases on enamel and dentin surfaces when comparing remineralization agents at both concentrations (p>0.05).

## Conclusions

The minimal remineralization effect was observed on the dentin surface with 2.5% nHap, while the agents demonstrated greater efficacy on enamel surfaces irrespective of concentration. Nano-hydroxyapatite incorporated with potassium nitrate, utilized for alleviating tooth sensitivity, will address mineral depletion and facilitate remineralization.

# Abstract: 04. Evaluation of surface properties of bioactive restorative Material exposed to gastric-acid

**Authors:** C.S. Olgunsoy, A. Uslu Tekce, N. Attar

**Affiliations:** *Department of Restorative Dentistry, School of Dentistry, Hacettepe University, Ankara, Turkey. serraolgunsoy@gmail.com*

## Aim

This study aimed to evaluate the surface roughness and microhardness of bioactive restorative Material exposed to gastric-acid, simulating conditions in patients with reflux or frequent vomiting. The null hypothesis was that there would be no significant difference in the surface roughness and microhardness of bioactive restorative Material before and after exposure to gastric acid.

## Material and Methods

Sixty specimens were prepared using five different restorative materials: a nanohybrid resin-composite (Filtek Z550, 3M), a giomer (Beautifil II, Shofu), an alkasite material (Cention Forte, Ivoclar), a resin-modified glass ionomer (Riva LCHV, SDI), and a glass hybrid restorative material (Equia Forte HT, GC). Twelve specimens per group were prepared using molds with a diameter of 5 mm and a thickness of 2mm. Baseline microhardness and surface roughness measurements (T1) were conducted after finishing/polishing using a Vickers microhardness tester and a profilometer, respectively. The specimens were then immersed in gastric-acid solution (pH=1.2) at 37°C for 7 days, with daily exposure for 18h followed by 6h in distilled water. Post-exposure (T2) measurements were performed. The Kruskal-Wallis and Wilcoxon Signed Ranks tests were used for statistical analysis.

## Results

Beautifil II and Cention Forte exhibited significant increases in surface roughness and decreases in microhardness after gastric-acid exposure (p<0.05). Equia Forte HT and Riva LCHV showed a significant decrease in microhardness (p<0.05), whereas no significant change in surface roughness (p>0.05). The greatest change in surface roughness was observed in Beautifil II, while the highest change in hardness was noted in Cention Forte. In contrast, Filtek showed no statistically significant differences in either parameter (p>0.05).

## Conclusions

Gastric-acid exposure significantly affects the surface properties of bioactive restorative materials. Beautifil II and Cention Forte exhibited the most significant changes, while Filtek demonstrated the highest resistance. These outcomes suggest that material selection is crucial for patients prone to acid exposure.

# Abstract: 05. Impact of clear polyvinyl siloxane molds on the microhardness of highly filled flowable composite resins

**Authors:** T. Aytas, E.K. Koç, E. Meral, U. Koç Vural

**Affiliations:** *Department of Restorative Dentistry, School of Dentistry, Hacettepe University, Ankara, Turkey. tolunay.aytas@gmail.com*

## Aim

This study aimed to comparatively evaluate the effect of clear polyvinyl siloxane (PVC) molds used in the injection molding technique on the microhardness of highly filled flowable composite resins.

## Material and Methods

Three different highly filled flowable composite resins were tested: G-ænial Universal Injectable (GUI), Filtek Supreme Flowable (FSF), and Tokuyama Omnichroma Bulk Flow (TOB). A total of 60 disc-shaped specimens were prepared using Teflon molds (n=20 per material). Each group was further divided into control and test groups (n=10 per subgroup). Control specimens were light-cured under a Mylar strip for 20 seconds, and microhardness (VHN) was measured. For the test group, Teflon molds were filled with the respective resin composite. The test specimens were light-cured under a 2 mm thick PVC mold for 20 seconds, and VHN was measured immediately. After removing the PVC mold, specimens underwent an additional 20 seconds of light curing, and VHN measurements were repeated. Data were analyzed using ANOVA, Tukey’s HSD, and independent sample t-tests (p<0.05).

## Results

The VHNs of GUI, FSF, and TOB were significantly lower when light-cured under a PVC mold compared to the control (p=0.217,0.014,0.172 respectively). However, VHN measurements significantly increased after additional curing following the removal of the PVC molds (p<0.001,0.021,0.001 respectively). Significant differences were also observed between the groups in VHN measurements obtained under the PVC mold (p<0.001) and after mold removal and additional curing (p<0.001).

## Conclusions

The use of PVC molds in the injection molding technique significantly reduced the microhardness of highly filled flowable composite resins. An additional light-curing step is essential following the removal of the PVC mold to achieve optimal material properties.

# Abstract: 06. Effect of remineralizing agents on surface properties after diode laser-assisted bleaching

**Authors:** K. Isikli, A. Uslu Tekce, E. Meral, F. Yalcin Cakir

**Affiliations:** *Department of Restorative Dentistry, School of Dentistry, Hacettepe University, Ankara, Turkey. kemal2315@gmail.com*

## Aim

The aim of this study was to evaluate the effects of different remineralizing agents on enamel surface hardness, roughness, and color stability following diode laser-assisted tooth bleaching. The null hypothesis tested was that remineralizing agents would not affect these surface properties after laser-assisted bleaching.

## Material and Methods

Sixty sound human enamel samples were prepared using silicon carbide abrasive papers (SiC, 600-1200 grit) to standardize the surfaces. Baseline measurements (T0) of microhardness (Vickers microhardness tester), roughness (Perthometer M2, Mahr), and color (Easy Shade, VITA) were recorded. Color measurements were performed using the CIELAB color space and the Delta-Eab formula. A diode laser-assisted bleaching procedure (Epic X, 940 nm, 7W, Biolase, Whiteness HP 35%, FGM) was performed on all samples. Post-bleaching measurements (T1) were recorded. Samples were randomly divided into four groups (n=15): Rocs Medical Minerals (ROCS), Curaprox Aligner Foam (Curaprox), Tooth Mousse (GC), and Fluor Protector (Ivoclar Vivadent) serving as the control group. Remineralizing agents were applied twice daily for 7 days, with all samples stored in distilled water between applications, following the specific recommendations for each agent.

## Results

After bleaching, effective whitening was achieved in all groups (p<0.05, Delta-Eab>3.3). No significant color difference was observed between T1 and T2. In terms of surface roughness, Tooth Mousse significantly reduced roughness (p=0.42), whereas Rocs and Curaprox showed no significant changes (p>0.05). In contrast, fluoride varnish significantly increased roughness (p<0.05). No significant differences in surface hardness were observed among the groups following the application of remineralizing agents (p>0.05).

## Conclusions

Remineralizing agents did not affect color stability or surface hardness after diode laser-assisted bleaching. However, they had a significant impact on surface roughness.

# Abstract: 07. Effect of beverages on the color stability of two types of enamel-shade composites with different fillers: an in vitro study

**Authors:** B. Alpay, E. Altin, B. Derel, O. Kardas, M. Tiryaki

**Affiliations:** *Department of Restorative Dentistry, Faculty of Dentistry, Istanbul University, Istanbul, Turkey. burcualpay1001@gmail.com*

## Aim

The aim of this study was the in vitro effects of different beverages on the color stability of two different enamel-shade composites used in the outermost layer of the layering technique in the anterior region.

## Material and Methods

Within the scope of the study, two different types of enamel-shade composites with nanofillers (3M Filtek Ultimate Enamel A2) and nanohybrid fillers (FGM Vittra APS Enamel A2) and 3 different beverage types (Nescafe Classic (Nestle, Bursa, Turkey), effervescent vitamin C (Redoxon, Bayer), Coca Cola and one control group (distilled water) were used. Total 80 discs, 40 of each composite, were prepared (10mm in diameter×2mm in thickness) and initial color measurements of all samples were made. The composite disks were randomly divided into 4 subgroups with 10 composite disks per beverage (n=10). All samples were kept in beverages for 4 weeks in total and color measurements were made along the study. Color change observed with Vita Easyshade (VITA Zahnfabrik, Bad Säckingen, Baden-Württemberg, Germany). In the change of color stability, the numerical values obtained with the spectrophotometer were analyzed with the CIEDE2000 formula.

## Results

In both groups, there was a statistically significant difference in ΔE00 levels between beverage types(p=0.001; p<0.05). Coffee had significantly higher ΔE levels than distilled water , cola and vitamin C. In the coffee group, the ΔE 00 levels of the nanofil composite were significantly higher than those of the nanohybrid group (p=0.002; p<0.05).

## Conclusions

In aesthetic restorations, color and color stability are very important for evaluating the success and clinical life of the restoration especially in enamel shade composites because they are the most affected by external discoloration. The reason for the difference in coloration between the composites can be that the nanofiller contents are more likely to be colored.

# Abstract: 08. Exploring Different Toothpaste’s Effect on the Microhardness of Bleached Enamel

**Authors:** B. Uygunoz, E.Y. Aybek, E. Ergin, A.R. Yazici, F. Yalcin Cakir

**Affiliations:** *Department of Restorative Dentistry, School of Dentistry, Hacettepe University, Ankara, Turkey. buseuygunoz@hotmail.com*

## Aim

This study aimed to evaluate the effect of different toothpastes on the surface microhardness of enamel following in-office tooth bleaching

## Material and Methods

Sixty intact bovine incisor teeth were selected for this study. After in-office laser bleaching (35% HP, Whiteness HP, FGM), the teeth were randomly assigned to five groups (n=12) for brushing with the following toothpastes: Regular Toothpaste (G1: Colgate Total 12), whitening toothpaste with poloxamer (G2: Colgate Optic White Oxygen), whitening toothpaste with hydrogen peroxide (G3: Colgate Optic White Expert), whitening toothpaste with charcoal powder (G4: Colgate Optic White Charcoal) and anti-erosive/desensitizing toothpaste (G5: Sensodyne ProEnamel). All groups underwent simulated brushing using a toothbrushing simulation machine, replicating two weeks of regular brushing. The microhardnesss values were measured at baseline (T0), after bleaching (T1), and post-brushing (T2) using a Vickers hardness device. Data were analyzed using One-Way ANOVA, Tukey-HSD and Kruskal Wallis tests (p<0.05).

## Results

Surface microhardness remained consistent over time (T0, T1, T2) across all tested toothpaste groups, except for the anti-erosive/desensitizing toothpaste group (G5) (p>0.05). In G5, a significant increase was observed in microhardness between the baseline (T0) and post-brushing after bleaching (T2) (p<0.05). However, no significant change was detected between T1 and T2 (p>0.05). The microhardness values among the all the test groups were similar at T0 and T1 (p>0.05). However, at T2, the microhardness of G3 was significantly higher than G2, while G5 demonstrated higher microhardness than both G2 and G4 (p<0.05).

## Conclusions

The findings suggest that choosing the right toothpaste formulations can play an important role in enamel microhardness following in-office tooth bleaching. Among the toothpastes tested, the current anti-erosive/desensitizing toothpaste showed the most promise in improving enamel microhardness, indicating its potential benefits for post-bleaching enamel care.

# Abstract: 09. Effect of tissue glue application on microhardness of mineral trioxide aggregate setting in various solutions - a pilot study

**Authors:** J. Matijević, A. Vlahovljak Ferušić, M. Vidov, A. Ivanišević, Z. Karlović, A. Baraba

**Affiliations:** *School of Dental Medicine, University of Zagreb, Zagreb, Croatia. matijevic@sfzg.hr*

## Aim

The aim of this study was to examine the impact of tissue glue application on the surface of freshly mixed MTA on its microhardness after setting when exposed to moisture and human blood.

## Material and Methods

Micro Mega MM-MTA material was mixed according to the manufacturer’s instructions and applied in custom made teflon molds, 6 mm in diameter and 4 mm in height. Four experimental groups (n=4) were as follows: control group (MTA+Phosphate buffer solution - PBS) (n=4), MTA coated with tissue glue (PerioAcryl)+PBS (n=4), MTA+blood+PBS (n=4) and MTA coated with tissue glue+blood+PBS (n=4). Experimental groups with blood exposure were exposed for 15 minutes, then rinsed and stored in PBS. Prior to the micro-hardness testing, all Material were incubated in PBS for seven days. The microhardness of samples was measured using a Vickers microhardness testing machine. Five measurements were performed for each sample (20 measurements per experiment group). For statistical analysis of microhardness, Kolmogorov-Smirnov normality test and analysis of variance was used.

## Results

Data distribution was normal in all groups. The results of the ANOVA test (df=3, F=28.965, p<0.001) with post hoc Tukey test showed statistically significant differences between the groups with and without tissue glue treatment, both in samples exposed to blood and in those that were not.

## Conclusions

Protection of the MTA using a tissue glue during the setting of the material significantly influences microhardness when exposed to blood and phosphate saline buffer solution. This indicates that tissue glue protection of the MTA material may be clinically used in situations when it is exposed to moisture.

# Abstract: 10. Effect of home-bleaching application on two bioactive restorative Material and a resin composite

**Authors:** E. Şahin, S. Akyuz, E. Ergin, F.D. Oz

**Affiliations:** *Department of Restorative Dentistry, School of Dentistry, Hacettepe University, Ankara, Turkey. dt.ecemsahin@gmail.com*

## Aim

The aim of this study was to evaluate the changes of different bioactive restorative Material in terms of microhardness, surface roughness and color change (ΔE, ΔWI) after home-bleaching for 15 days.

## Material and Methods

8x8x3 sized restorative material samples were prepared from 3 different restorative Material (n=10): a resin composite (RC) (Harmonize/ KERR), an alcasite (AL) (Cention N/IVOCLAR VIVADENT) and a high viscosity glass ionomer (HVGIC) (EQUIA/GC). After polishing with silicone carbide papers, the microhardness values, the surface roughness values and colour of the restorative Material were measured. The specimens were then subjected to a home-bleaching procedure for 15 days according to the manufacturer’s instructions. The final measurements were performed and the data were subjected to statistical analysis with Duncan, One-way ANOVA, Kruskal-Wallis and Wilcoxon Signed Rank tests (p<0.05).

## Results

No significant difference was observed in microhardness values within the tested Material after home bleaching procedure when compared to baseline (p>0.05). Percentage changes in microhardness and surface roughness were compared across the materials, no differences were found (p>0.05). It was observed that ΔE value was highest in the HVGIC group, and it was significantly greater compared to the RC and AL groups (p< 0.05). However, no significant differences were found among the ΔWI (p>0.05). In terms of surface roughness, when each material was compared within itself, it was found that the surface roughness of AL decreased following the whitening procedure (p<0.05).

## Conclusions

This in vitro study demonstrated that home-bleaching agents did not affect the microhardness and surface roughness of the tested bioactive restorative Material but affected HVGIC more than RC and AL in terms of colour change. Therefore, the effect of bleaching on the restorative material selection might be critical when considering patient-specific factors, the location of the restoration, and its role within the overall aesthetic plan.

# Abstract: 11. Effects of bioactive restorative Material on neighboring enamel after home-bleaching

**Authors:** S. Akyüz, E. Sahin, F.D. Oz, E. Ergin

**Affiliations:** *Department of Restorative Dentistry, School of Dentistry, Hacettepe University, Ankara, Turkey. serraakyuz96@gmail.com*

## Aim

To evaluate the effects of different bioactive restorative Material on the neighboring enamel in terms of color change (ΔE, ΔWI), microhardness and surface roughness after 15 days of home-bleaching.

## Material and Methods

Thirty intact bovine incisors were collected and embedded in acrylic resins. Standard preparations (8x8x3 mm) were made on one side of each embedded tooth. The specimens were randomly divided into three groups (n=10) based on the restorative material used to fill the preparations, in contact with the embedded tooth: (i) control group – resin composite (RC) (Harmonize/KERR), (ii) alcasite group (AL) (Cention N/IVOCLAR), and (iii) high-viscosity glass ionomer group (HVGIC) (EQUIA/GC). The initial color, microhardness and the surface roughness values of the neighbouring enamel were measured. The specimens were then subjected to home-bleaching procedure (Total Blanc Home C16/DFL) for 15 days following the manufacturer’s instructions. Color, microhardness, and surface roughness measurements were repeated and the data were subjected to statistical analysis with Duncan, One-way ANOVA, Kruskal-Wallis and Wilcoxon Signed Rank tests (p<0.05).

## Results

The ΔE, ΔWI, and roughness values of the enamel neighbouring to the restorative Material were similar before and after bleaching (p>0.05). A significant increase was observed on the roughness of the enamel adjacent to HVGIC after bleaching (p<0.05), whereas no significant difference was found in the other groups (p>0.05). The microhardness of enamel neighbouring to HVGIC and RC significantly decreased after bleaching (p<0.05), however no change was observed on the AL group (p>0.05).

## Conclusions

The tested bioactive restorative Material affected the neighboring enamel differently after home-bleaching. AL demostrated the most stable enamel properties, with no significant changes in microhardness or roughness, while HVGIC caused significant roughness and microhardness changes. RC also reduced the enamel microhardness, emphasizing the importance of choosing adequate restorative Material to minimize bleaching-induced adverse effects on the neighbouring enamel.

# Abstract: 12. Effects of whitening toothpastes on color stability and surface roughness of a giomer

**Authors:** E. Dermanli, E. Ozturk

**Affiliations:** *Department of Restorative Dentistry, School of Dentistry, Hacettepe University, Ankara, Turkey. ekinder2@hotmail.com*

## Aim

This study aimed to evaluate the effects of different whitening toothpastes on the color stability and surface roughness of a giomer before and after immersing in soft beverages.

## Material and Methods

A total of 64 disk-shaped specimens (Beautifil II, Shofu) were prepared and randomly divided into two groups. The groups were immersed in freshly prepared coffee and cola solutions for 1 hour daily. After staining, the specimens were randomly divided into four subgroups and brushed for 8 seconds using an electronic toothbrush (Oral-B iO 9) with the following whitening toothpastes: Opalescence Whitening (OW), Sensodyne Clinical White (SCW), Zubio Whitening Toothpaste (ZWT), and a non-brushed control group (n=8 per group). Color changes (DeltaE) were measured using a spectrophotometer, and surface roughness (RA) was assessed using a profilometer at baseline and after 14 days of staining and brushing. Data were statistically analyzed using Duncan’s and Independent Samples T-Test for color measurements, and Kruskal-Wallis and Mann Whitney-U for roughness measurements.

## Results

Toothpastes didn’t exhibit significant differences in color changes in any solution (p>0.001). However, the control group showed significantly higher color change compared to the toothpaste groups (deltaEcoffee-control=11.54, deltaEcola-control=2.66; p<0.001). In the coffee solution, none of the whitening toothpastes caused significant differences in surface roughness compared to the control group (pO=0.141, pSC=0.073, pZ=1.0; p>0.05). However, a significant difference was found between Sensodyne and Zubio (p=0.043<0.005). In the cola solution, Opalescence and Zubio did not cause significant differences in roughness compared to the control group (pO=0.063, pZ=1.0; p>0.05). Sensodyne caused a significant increase in surface roughness compared to the control group (pS=0.022<0.05). Significant differences were also found between Sensodyne and Zubio (pS-Z=0.007) and between Opalescence and Zubio (pO-Z=0.022) groups.

## Conclusions

Whitening toothpastes demonstrate beneficial effects in reducing the discoloration of composite resin restorations; however, their use may lead to an increase in surface roughness.

# Abstract: 13. Impact of polyvinyl siloxane mold thickness on surface gloss of highly filled flowable composite resins

**Authors:** E.K. Koç, T. Ayta, E. Mera, U. Koç Vural

**Affiliations:** *Department of Restorative Dentistry, School of Dentistry, Hacettepe University, Ankara, Turkey. e.k.koc93@gmail.com*

## Aim

The aim of this study was to evaluate the effect of clear polyvinyl siloxane (PVC) mold thickness used in the injection molding technique on the surface gloss of two highly-filled-flowable composite resins.

## Material and Methods

A total of 80-disc-shaped (diamater 10mm X height 2mm) specimens were prepared using two highly-filled-flowable composite resins- G-ænial Universal Injectable(GUI) and Filtek Supreme Flowable(FSF) (n=40, per material)-with Teflon molds. Each group was divided into a control and three test subgroups(n=10, per group). The specimens of control groups were prepared by injecting the material into the mold and light-curing under Mylar strip for 20s. In the test groups, after the molds were filled with materials, a PVC mold (Exaclear) with 2 mm thickness was placed on the resin composite and then light-cured for 20s. After removal of the PVC mold, an additional 20s of light-curing was applied. The same sequence was followed for 3mm and 4 mm PVC mold test groups. Surface gloss values were measured with a glossmeter. Data were analyzed statistically (p<0.05).

## Results

There were significant differences among the GUI specimens in the test groups regarding suface gloss (p< 0.05). Control group showed higher gloss values compared to test groups (p<0.001). Regarding FSF, no significant differences were observed across all groups (p>0.05). A comparison between control groups of GIU and FSF revealed, significant differences (p<0.001). GUI exhibited higher gloss values than FSF in the control group (p<0.001), No significant differences were found between Material in the 2mm and 4mm groups (p=0.605, p=0.293, respectively), whereas FSF showed higher gloss in the 3mm group (p=0.013).

## Conclusions

Since surface gloss is an important parameter affecting the aesthetic success of anterior restorations, it should be kept in mind that this parameter may vary depending on the material-specific differences and the thickness of the PVC mold in material selection when injection moulding technique employed.

# Abstract: 14. Hyaluronic acid as a therapeutic approach for gingival aesthetics

**Authors:** S. Abularach Roca, L. Horcajo, F. Muscillo, S. Treviño, C. Benito, C. Martín Carreras-Presas

**Affiliations:** *European University of Madrid, Madrid, Spain. sabularachr@gmail.com*

## Aim

This study aims to evaluate the efficacy of hyaluronic acid (HA) in regenerating interdental papillae and addressing aesthetic deficiencies, such as “black triangles” with a specific focus on the durability of clinical outcomes.

## Material and Methods

A bibliographic review was conducted, analyzing 11 studies published in the last eight years, including randomized clinical trials and systematic reviews, with a total of 92 patients. Participants underwent HA injections targeting interdental papillae deficiencies. Outcomes were measured through photographic analysis, standardized molds, and clinical indices, including interdental papilla index (IPI), aesthetic evaluation scales, and black triangle area reduction.

## Results

HA injections resulted in a mean reduction of 41% in the black triangle area and improved aesthetic scores based on clinical indices. The effects were temporary, with a mean duration of 6 months. Mild adverse events, such as localized pain and swelling, were reported in 30% of cases, resolving spontaneously within days. No severe complications were observed. Patients’ satisfaction scores exceeded 85% in all studies.

## Conclusions

HA is an effective and minimally invasive therapeutic option for the aesthetic reconstruction of interdental papillae. While its clinical benefits are significant, its limited duration necessitates repeated applications, which may influence patient compliance and overall cost-effectiveness.

# Abstract: 15. The effect of different polishing systems on the color stability and surface roughness of an universal composite resin

**Authors:** C. Bayraktutan, U. Koc Vural, A. Kiremitci

**Affiliations:** *Department of Restorative Dentistry, School of Dentistry, Hacettepe University, Ankara, Turkey. cerenhacettepe1@gmail.com*

## Aim

This study investigated the influence of different polishing systems on the color stability and surface roughness of a nanohybrid universal resin-based composite

## Material and Methods

A total of 60 disk-shaped specimens (10X2 mm) were prepared using a nanohybrid universal resin-based composite (Filtek Z550, 3M ESPE) and light-cured under Mylar strip for 20s. The specimens were then divided into 5 subgroups (n=12, per group) based on the polishing procedure: unpolished control (C), OptiDisc (OD), Diamond Polish Mint (DPM), Enhance PoGo (EPG), and DiaComp Twist Plus (DCTP). Baseline color measurements were obtained using a spectrophotometer and surface roughness measurements were obtained using a profilometer device. Then, polished specimens were immersed in coffee solution at 37°C for 5 days. After immersion, all measurements were repeated, and color change values (ΔE00) were calculated. Data were analysed statistically (p<0.05).

## Results

There were significant differences among the groups in color change (p<0.001). The highest ΔE00 was detected in the following order DPM > OD > EPG > DCTP > C groups. The differences between DPM and OD, EPG, DCTP, and C groups were significant (p=0.005, p=0.007, p<0.001, p<0.001). Regarding surface roughness, at baseline, the highest surface roughness was found in the following order DPM > OD > C> EPG > DCTP groups with significant differences (p=0.0314). After coffee staining, surface roughness was increased in all groups compared to baseline significantly (p<0.001). The highest surface roughness was found in DPM > OD > EPG > DCTP > C groups (p<0.05). DPM group showed significantly higher roughness values than C group (0.019).

## Conclusions

Clinicians should be aware that the polishing system used may have an effect on changes in both the surface roughness and color stability of composite resins due to coloring foods over time.

# Abstract: 16. Immediate vs delayed dentinal sealing: a 5-year microtensile bond strength evaluation

**Authors:** C. Orsello, G. Gennari, R. Chiara, A. Comba, A. Baldi, N. Scotti

**Affiliations:** *Dental School, University of Turin, Turin, Italy. caterina.orsello@edu.unito.it*

## Aim

The aim of this in vitro study is to evaluate the microtensile bond strength (μTBS) to coronal dentin using different universal adhesives both in self-etch and total-etch technique. Immediate and delayed dentinal sealing (IDS and DDS) were evaluated, both at baseline and after 5-years.

## Material and Methods

One-hundred-twenty extracted sound molars were selected and the coronal dentin was exposed. Prepared teeth were divided into two groups: freshly milled coronal dentin (G1, IDS) and 1-month incubation in 37°C sterile water (G2, DDS). Each group was then divided according to the adhesive system used and the etching approach: - SG1: 3-step etch-and-rinse (ER) adhesive (Scotchbond, 3M), servig as control - SG2: Universal adhesive with MDP in ER mode (Clearfil Universal Quick) - SG3: Universal adhesive with MDP in self-etch (SE) mode (Clearfil Universal Quick) - SG4: Universal adhesive without MDP in ER mode (Futurabond U) - SG5: Universal adhesive without MDP in SE mode (Futurabond U) Resin composite buildups of 4mm were made, then teeth were sectioned along to obtain 1x1 mm sticks in accordance with the μTBS protocol. Sticks were stressed to failure at baseline and after 5 years. Each specimen was observed with stereomicroscope to determine the failure mode: adhesive (A), cohesive (C) or mixed (M). Data were statistically analyzed by ANOVA and Tukey post-hoc test, while Chi-squared test was used for failure mode analysis. Statistical significance was set for p<.05.

## Results

All the investigated factors significantly influenced μTBS. ER performed better than SE, IDS gave higherbond strength values compared to DDS and storage over time significantly affected μTBS.

## Conclusions

IDS achieved higher bond strength values both at baseline and after 5-years aging.

# Abstract: 17. Optical stability and surface analysis of 3D printed resin-based composite vs milled materials

**Authors:** A. Bardine, F. Bosello, C. Rolando, P. Iacomussi, A. Baldi, N. Scotti

**Affiliations:** *C.I.R. Dental School, Lingotto, University of Turin, Turin, Italy. anna.bardine@edu.unito.it*

## Aim

The purpose of this in vitro study was to evaluate the optical properties and surface analysis of different 3D printed resin-based composite (RBC) compared to conventional milled materials, before and after thermal aging.

## Material and Methods

A single full crown, designed with a CAD software, was fabricated with different methods, for a total of 40 crowns divided in 8 groups (n=5 each), according to the material employed: 1. G1: 3D-printed micro-hybrid composite (Iris Plus, DWS) 2. G2: 3D-printed, heavy-loaded micro-hybrid composite (Iris Max, DWS) 3. G3: Milled lithium disilicate with high translucency (Initial Lisi HT, GC) 4. G4: Milled lithium disilicate with low translucency (Initial Lisi LT, GC) 5. G5: Milled composite with high translucency (Cerasmart HT, GC) 6. G6: Milled composite with low translucency (Cerasmart LT, GC) 7. G7: Control group, milled lithium disilicate with high translucency (E-Max CAD HT, Ivoclar) 8. G8: Milled feldspathic-based hybrid ceramic with high translucency (VITA Enamic HT, VITA) Once finished and polished according to individual manufacturer instruction, samples were tested to evaluate optical properties. Spectro-colorimetric parameters included: spectral factor of re-reflection and transmission, CIE Lab colorimetric coordinates, yellow index, translucency index measured as the variation of spectral factor of reflection and/or transmission with respect to substrate color. Finishing parameters included the evaluation of the gloss index, nanoindentation and roughness through laser profilometry. After beforementioned tests, samples underwent thermal aging with a standard protocol consisting of 10.000 cycles at 5-55°C. Then, samples were resubmitted to previous tests and data were collected for baseline and after-aging comparison. Statistical analysis consisted in ANOVA test, with significancy p<0.05.

## Results

3D-printed Material tested (G1 and G2) showed optical and surface properties similar to milled resins (G5, G6), with no significant differences among them (p<0.05).

## Conclusions

3D-printed Material seem a promising alternative to milled composites, but present significant differences compared to ceramics.

# Abstract: 18. The effect of whitening toothpastes on the color stability of injectable resin composites

**Authors:** C. Atalay

**Affiliations:** *Department of Restorative Dentistry, School of Dentistry, Hacettepe University, Ankara, Turkey. cansuatalaydr@gmail.com*

## Aim

The aim of this in-vitro study was to evaluate the changes in the color and whitening index of injectable resin composites after brushing with four different kinds of whitening toothpaste, following coffee immersion.

## Material and Methods

Eighty-disc shaped samples (8mmøx2mm) were fabricated from two different resin composites; G-aenial Universal Injectable and G-aenial ACHORD (GC Corp). After 7 days of coffee immersion, brushing simulation was applied for 7, and 15 days with four different whitening toothpastes. The subgroups were arranged according to the tested different toothpastes were as follows (n=10): Group 1: Colgate Total 12 (CT; Colgate Palmolive, USA) (regular toothpaste as control), Group 2: Opalescence Whitening (OW; Ultradent Products, USA), Group 3: Colgate Optic White with Charcoal (COW; Colgate Palmolive, USA), Group 4: Colgate 2 in 1 Whitening (C2W; Colgate Palmolive, USA). The color of all specimens was measured at the baseline, after initial staining, and on days 7, and 14 using a spectrophotometer (VITA Easyshade, Zahnfabrik, Germany). Color stability was evaluated based on the CIEDE2000 color difference (ΔE00), and differences in whiteness were determined using the whitening index for dentistry (WID). Data were analyzed using one-way ANOVA and Tukey tests.

## Results

G-aenial ACHORD groups showed more discoloration compared to G-aenial Universal Injectable groups after coffee immersion (p<0.05). The color change for both resin composites was above the clinically acceptable limit (ΔE00>1.8). Color change over time after brushing with whitening toothpastes differed significantly for all groups (p<0.05). OW and COW exhibited a significantly higher whitening effect than C2W (p<0.05). No significant difference was found between composite groups brushed with C2W (p>0.05), but significant differences were noted between groups brushed with OW and COW (p<0.05).

## Conclusions

Injectable resin composites may be less prone to discoloration than conventional paste-like resin composites. Opalescence Whitening and Colgate Optic White with Charcoal reduced discoloration within the clinically acceptable perceptibility range.

# Abstract: 19. Nanohydroxyapatite/proanthocyanidin chitosan membranes for tissue engineering

**Authors:** T. Enrich, S. González López, C. Cifuentes Jiménez, A. Rodríguez Navarro

**Affiliations:** *University of Granada, Granada, Spain. tattiana@ugr.es*

## Aim

This study aimed to create a bioactive membrane by combining a chitosan biopolymer (CS) with hydroxyapatite nanoparticles functionalized with proanthocyanidin (nHAp_PA). This strategy combines the remineralizing and collagen-strengthening functions of nanoparticles with the biocompatible and antibacterial potential of chitosan.

## Material and Methods

nHAp_PA was synthesized as previously described. The biocompatibility of the nanoparticles was evaluated by a cytotoxicity assay on fibroblasts. To fabricate the membranes, chitosan powder (3g) was dissolved in 100 mL of aqueous acetic acid solution (1% w/v), and nHAp_PA nanoparticles were incorporated at 2% (w/v) and mixed until homogenization. The membranes were then frozen at -18 °C for 24 h and freeze-dried for 72 h. Control samples of the chitosan membranes were prepared following the same protocol, except for the addition of nHAp_PA. The membrane characterization was carried out with ATR-FTIR, TGA, SEM, and AFM.

## Results

The nHAp_PA promoted high cell viability, and the CS_nHAp_PA membranes were effectively synthesized. ATR-FTIR showed characteristic CS and nHAp_PA peaks at 890 cm-1, 1060 cm-1, 1150 cm-1, 1340 cm-1, 1400 cm-1, 1540 cm-1, and shoulder emergence of shoulder at 1100 cm-1. The TGA results demonstrated that the addition of nHAp_PA improved the thermal stability of the CS membranes. SEM and AFM confirmed the porous structure of the membranes.

## Conclusions

Based on these preliminary results, CS_nHAp_PA is a biocompatible material that improves the structural stability of CS membranes upon the addition of nHAp_PA. These membranes combine the remineralizing and collagen-reinforcing properties of nHAp_PA and the antibacterial and biocompatible porous structure of CS membranes, which is a key feature that enables cell attachment and differentiation, as well as nutrient transport. Therefore, this is a promising biomaterial that could be used in tissue engineering, specifically for the dentin-pulp complex and periodontal regeneration.

# Abstract: 20. Masking abilities of layering techniques using composite resins with different viscosities

**Authors:** E.C. Taptal, İ.N. Çalışkan, M. Türk, G. Ozan, H. Şar Sancaklı

**Affiliations:** *Faculty of Dentistry, Istanbul University, Istanbul, Turkey. elifcemretaptal@gmail.com*

## Aim

The present study aimed to evaluate the masking abilities of composite resins with different viscosities, opacities and thicknesses by using single or multi-layering techniques. The hypothesis of our study is that different layering techniques with highly-filled flowing composites would have a limited significant effect on masking ability of dark substrates.

## Material and Methods

120 composite discs (2mm*8mm, 1.5mm*8mm) (G-Aenial A’Chord-G-Aenial Universal Injectable) were obtained by stainless steel mold and both sides were polymerized with LED light-curing unit (Elipar) for 40s. Samples were divided into two groups according to thickness: 1) 1.5mm*8mm; 2) 2mm*8mm and each group divided into six subgroups according to their viscosity and shade combination due to layering technique. Measurements performed with a spectrophotometer (Vita EasyshadeV) from 3 different points. Normality and homogenity of the optical data were confirmed by using the Shapiro–Wilk test with p<0.05 significance with Kruskal-Wallis test.

## Results

CIEDE2000 formula and TP (translucency parameter) were used to evaluate masking ability and translucency of composites. Composites with higher TP values showed lower masking potential. Various composites showed significantly different opasities (p<0.001), moreover, the highest masking were obtained from our negatif control groups (G-anieal A’Chord Bleach) although, the lowest was the layering group of injection. Layering thickness have a significant difference and showed lower masking ability yet higher translucency (p<0.05).

## Conclusions

It can be concluded that layering strategy influences the masking ability of composite resins over discolored substrates. Due to higher translucency of injectable composites; 2mm layers exerts more acceptable masking rather than 1,5mm layers. Within the limitation of the present study, masking ability of injectable composites can be determined as a promising technique.

# Abstract: 21. Optical properties of katana yml cubic zirconia related to sintering procedures

**Authors:** F. Tassone, E. Oreglia, C. Rolando, A. Baldi, A. Comba, N. Scotti

**Affiliations:** *C.I.R. Dental School, Lingotto, University of Turin, Turin, Italy. tassone.federica@gmail.com*

## Aim

The aim of this in vitro study was to evaluate the optical properties of Katana YML cubic zirconia subjected to different sintering settings, in terms of volume occupied in the sintering oven and firing temperature.

## Material and Methods

Several models of “Crown-shaped” specimens were prepared with a flat occlusal surface and an occlusal thickness of 1.5mm. The zirconia samples (Katana YML) were milled according to the manufacturer's procedures and all come from the same zirconia production batch. The crowns had to meet the required dimensional parameters. The crowns were produced in 2 different firing ovens; for each oven, the samples were divided based on the temperatures (1500C°, 1550C° and 1600C°) at which they were sintered. Furthermore, always respecting the subdivision by oven and temperature, the samples were further subdivided based on the volume occupied in the sintering oven: they were sintered either in a single quantity or in quantities of 9 samples at a time. The samples were tested to evaluate the optical properties: spectro-colorimetric parameters, spectral re-reflection and/or transmission factor, CIE Lab colorimetric coordinates, yellowness index, translucency index measured as variation of the spectral reflection and/or transmission factor with respect to the color of the substrate, optical finishing parameters as brightness index. After the previous tests, samples underwent thermal aging with a standard protocol consisting of 10.000 cycles at 5-55°C. Then the samples were resubmitted to previous tests for comparison.

## Results

This study demonstrates that both different temperatures and a diverse number of crowns in the same oven do affect the aforementioned final optical properties (p<0,05).

## Conclusions

Based on the results of the study, the volumetric occupation in the firing chamber and the firing temperature influence the spectro-colorimetric parameters of the samples.

# Abstract: 22. Bond strength and durability of graphene oxide-enhanced universal adhesive

**Authors:** M. Ugurlu

**Affiliations:** *Department of Restorative Dentistry, Faculty of Dentistry, Süleyman Demirel University, Isparta, Turkey. dtmuhittinugurlu@gmail.com*

## Aim

To evaluate the effect of incorporating graphene oxide into a universal adhesive on dentin bond strength and durability.

## Material and Methods

Forty caries-free human third molars were used. The teeth were randomly assigned to four groups (n=10). Experimental adhesive groups were prepared by adding graphene oxide in different concentrations (0.5, 1, and 3 wt%) to a universal adhesive (Scotchbond Universal). The adhesives were applied in self-etch mode. After composite buildups were bonded to the mid-coronal occlusal dentin surfaces, the specimens were stored in water (37°C for 24 hours) and then sectioned into micro-specimens. Ten micro-specimens were obtained from each tooth. Half of the micro-specimens were immediately subjected to microtensile bond strength (μTBS) testing (1.0 mm/min), while the other half was stored in water (37°C) for six months before testing. Failure analysis was performed using a stereomicroscope and scanning electron microscopy (SEM). Data were analyzed using one-way repeated measures ANOVA and Tukey’s test (p=0.05).

## Results

The addition of 0.5% and 1% graphene oxide to the universal adhesive significantly increased bond strength and durability (p<0.05). However, adding 3% graphene oxide resulted in a significant decrease in bond strength and durability (p<0.05). The highest immediate and aged bond strength values were observed in the 1% graphene oxide group, while the lowest values were recorded in the 3% graphene oxide group (p<0.05).

## Conclusions

The findings indicate that incorporating graphene oxide into a universal adhesive may enhance its bond strength and durability. However, increasing the concentration of graphene oxide beyond a certain threshold may have a detrimental effect on the adhesive's performance.

# Abstract: 23. The effect of different occlusal correction on crack initiation and propagation in metal-free materials

**Authors:** C. Gallesio, R. Sayed Ahmed, E. Sponzilli, A. Baldi, A. Comba, N. Scotti

**Affiliations:** *C.I.R. Dental School, Lingotto, University of Turin, Turin, Italy. cristina.gallesio@edu.unito.it*

## Aim

The study aims to analyze the effect of different occlusal corrections on crack initiation and propagation in lithium disilicate, cubic zirconia and hybrid ceramic Material used for CAD/CAM chairside restorations; and to determine the crack growth pattern in ceramic material with different occlusal correction and the crack initiate in ceramic material under cyclic fatigue. The null hypothesis tested was that there is no difference between the CAD/CAM chairside material and different occlusal correction on crack initiation and crack propagation during fatigue.

## Material and Methods

Four types of monolithic CAD/CAM Material (Initial LiSi Block, Cerasmart270, IPS Emax CAD, Katana Zirconia Block STML) were selected to mill identical flat-surface single crown restorations that were cemented on the replicated acrylic preparations. The specimens of each material were randomly allocated into 3 different groups: 1) No intervention (control specimens), 2) Fine football shaped diamond bur, 3) Fine football shaped diamond bur + polishing. A chewing simulator (SD Mechatronik) was used for fatigue cycling mechanical aging of the specimens in wet condition. Fatigue damage was evaluated by using optical coherence tomography at the baseline and after every cycle to reveal the presence of cracks. Furthermore, SEM analysis was performed to analyze the presence of cracks. Obtained data were statistically analyzed with a three-way ANOVA test and post-hoc Tukey (p<0,05).

## Results

From the statistical analysis, cracks form and propagate more in lithium disilicate compared to other materials. Additionally, occlusal corrections have a greater impact on cracks in group 3 compared to the other groups.

## Conclusions

The null hypothesis can be rejected since the type of material and the type of occlusal correction influence the formation and extension of the crack.

# Abstract: 24. 1-year in vitro study of tooth discoloration induced by hydraulic calcium silicate cements after pulpotomy

**Authors:** O. González-Castro, L. Ceballos, V. Fuentes

**Affiliations:** *Rey Juan Carlos University, Madrid, Spain. olga.gonzalez.castro@urjc.es*

## Aim

The aim of this study was to compare coronal tooth discoloration over a 1-year period following full pulpotomy in anterior teeth using different hydraulic calcium silicate cements (HCSCs).

## Material and Methods

Eighty anterior extracted teeth underwent coronal pulpotomy. Half were filled with blood, and the other half with serum. The teeth were divided into five groups (n=8): 1) Control group, 2) Biodentine, 3) ProRoot MTA, 4) NeoMTA 2, 5) NeoPUTTY. Color parameters (L*a*b*) were measured before HCSC placement and at 1 week, 2 weeks, 1 month, 3 months, 6 months, and 12 months. Color change was calculated by ΔEab and ΔE00. Repeated Measures ANOVA with Tukey post-hoc tests were performed (p<0.05).

## Results

All experimental groups exceeded the threshold considered acceptable (ΔE00 1.77–ΔEab 3.3) from the first week. No significant differences were found between groups in serum at any time point (p>0.05). However, in the blood medium, significant differences were observed at one week (ΔE00 p=0.020), one month (ΔE00 p=0.010; ΔEab p=0.007), and one year (ΔE00 p=0.045; ΔEab p=0.011). NeoPUTTY exhibited more discoloration than the control group at one week but less than ProRoot MTA at one month. After a year, teeth treated with ProRoot MTA showed higher color change than other HCSCs tested. Differences along the time in ΔEab were detected for tooth treated with ProRoot MTA in serum medium (p=0.008), and ΔE00 for ProRoot MTA and control group in both media (p<0.05).

## Conclusions

All Material tested produced a color change that was not acceptable from the first week after pulpotomy, regardless of the presence of blood or serum. Only ProRoot MTA increased color changes over the time. After a year, no differences in discoloration were detected between the bioMaterial regardless of the medium.

# Abstract: 25. Sustainable preparation of lipid nanoparticles by green chemistry for periodontal diseases

**Authors:** C. Cifuentes Jiménez, S. Contreras, M. Toledano, R. Osorio

**Affiliations:** *University of Granada, Granada, Spain. caro88cifuentes@gmail.com*

## Objectives

To synthesize and characterize lipid nanoparticles from beeswax by a biosynthetic technique using lecithin/Tween 80 as stabilizing agents, and Icariin (Epimedium alpinum) as a loading material.

## Material and Methods

Lipid nanoparticles (LNPs) were synthesized using beeswax as precursor agent and Icariin as loading material. The morphology of LNPs was determined by Transmission Electron Microscopy. The size was measured by DLS and the surface charge was assessed by Zeta- Potential. ATR-FTIR analysis established the composition of the LNPs and the effectiveness of their loading.

## Results

TEM showed the spherical shape of the LNPs, DLS analysis indicated an average particle size between 100 and 150 nanometers, the potential zeta measurement was -43. The ATR-FTIR spectrum indicated the presence of beeswax and Icariin.

## Conclusions

Lipid nanoparticles were successfully biosynthesized and characterized using green resources. It can be stated that beeswax acts as a precursor agent. In addition, effective loading of Icariin into the nanoparticles was achieved.

# Abstract: 01. Effect of trunatomy™, protaper next®, mtwo® and reciproc® systems on canal transportation, centring ability, and crack formation

**Authors:** L. Silvestre Cuesta, M. Rodríguez Pérez, P. González Villafranca, C. Benavides Reyes, C. Lucena Martín

**Affiliations:** *Master’s Degree in Aesthetic Multidisciplinary Dentistry, University of Granada, Granada, Spain. laurasilvestre@correo.ugr.es*

## Introduction

Traditional endodontics has been improved with the introduction of Nickel-Titanium instruments. Its most notable characteristics are its shape memory and superelasticity, and its main drawback is the fracture within the root canal. For that reason, new alloys and thermal treatments have been introduced to increase resistance to fatigue. The aim of this study was to compare the new TruNatomy™ files (Dentsply) with other commonly used files in dental practice: ProTaperNext® (Dentsply), Reciproc® (VDW, Munich, Germany), and MTwo® (VDW).

## Material and Methods

The mesial canals of 20 mandibular molars were subjected to computed tomography (CBCT) to analyse the initial morphological characteristics. The samples were distributed into four experimental groups according to the endodontic instrumentation system used (n=10) and were instrumented according to the manufacturer's instructions. After the instrumentation of the root canal, the samples underwent a second CBCT scan, and pre- and post-instrumentation data were examined to evaluate the following parameters: canal transportation, centring ability, area, and volume of dentine removed. After exploring the data distribution (Shapiro-Wilk test), parametric tests (ANOVA and Bonferroni) were applied. A significance level of p<0.05 was established.

## Results

No statistically significant differences were found between the different systems, except for canal transportation in the apical third between TruNatomy™ and Reciproc® (p=0.034).

## Conclusions

All tested systems have similar capabilities for transportation and centring in the root canal. Therefore, all systems can be used with a minimal risk of procedural errors for root canal preparation.

# Abstract: 02. A comparative of three Material used in vital pulp therapy: MTA, biodentine and iroot bp plus - literature review

**Authors:** J.J. Bofill Berdery

**Affiliations:** *International University of Catalonia, Barcelona, Spain. joanjordibofill@uic.es*

## Aim

Currently, we are experiencing a shift within dentistry, advocating for a more conservative approach, resulting in more personalized treatment. Due to advancements in technology, and particularly in biomaterials, the approach has become more predictable and practiced by dentists, supported by scientific evidence and the development of guidelines from leading scientific societies. The justification for this review aims to gather updated information on vital pulp therapy and the bioMaterial used, collecting health documents produced up to this point, and verifying if there has been a change in the perception of professionals. GENERAL To know and analyze the main strategies used in vital pulp therapy and the Material currently available. SPECIFIC To examine and understand the differences between MTA, Biodentine, and iRoot BP Plus in vital pulp therapy.

## Material and Methods

Review of the scientific literature by consulting different relevant databases.

## Results

The result of this review indicates that each material has its own advantages and disadvantages, and the success of the treatment does not only depend on the choice of material, but other factors may be considered as well.

## Conclusions

There is still a need for further research in order to develop an agreed protocol.

# Abstract: 01. Fiber-reinforced splints in the anterior region: a 23-year retrospective clinical insight

**Authors:** N. Ünlü, M.S. Velioglu, Y.E. Balaban, E.E. Mutafcilar Velioglu

**Affiliations:** *Department of Restorative Dentistry, Faculty of Dentistry, Selçuk University, Konya, Turkey. nunlu@selcuk.edu.tr *

## Aim

This study aimed to evaluate the long-term clinical performance of fiber-reinforced periodontal splints (FRSs) placed in the anterior region.

## Material and Methods

Thirty-eight patients who received FRSs between 2001 and 2024 were recalled for clinical evaluation. The restorations were assessed based on anatomical form, marginal adaptation, marginal discoloration, secondary caries, and retention using the modified United States Public Health Service (USPHS) criteria. Photographic documentation was taken to evaluate the current condition of the restorations, ranging from 3 to 23 years post-treatment. Patient satisfaction was assessed using a visual analogue scale (VAS), and periodontal pocket depths were measured during follow-up. Statistical analysis was performed using the Chi-squared test with a significance level set at p<0.05.

## Results

Significant differences were observed between the baseline and follow-up values across all modified USPHS criteria categories (p<0.05). However, no statistically significant differences were found when the clinical evaluation criteria were compared with each other (p>0.05). Most FRS restorations demonstrated repairable composite resin defects and secondary caries. Among the 38 patients who responded, 62.5% (n=32) rated their satisfaction with the FRS restorations as 90–100 on the VAS scale, 25% (n=4) rated them as 80–90, and 12.5% (n=2) rated them as 70–80. The periodontal pocket depths ranged from 2.3 to 3.8 mm at 3 years and 2.4 to 5.2 mm at 23 years post-treatment.

## Conclusions

Fiber-reinforced splints (FRSs) utilized for the management of periodontal tissue loss have demonstrated clinical effectiveness and durability for a minimum of 10 years. These restorations significantly prolong the functional lifespan of compromised teeth, thereby delaying or preventing the need for more invasive and costly treatment modalities such as dental implants and prosthetic restorations. FRSs represent a conservative, cost-effective, and practical therapeutic option for patients with periodontal disease.

# Abstract: 02. Internal gap evaluation of resin nanoceramic inlay with self-adhesive cement

**Authors:** S.H. Han, Y. Shimada, A. Sadr

**Affiliations:** *Department of Conservative Dentistry, St. Vincent’s Hospital, College of Medicine, Catholic University of Korea, Seoul, South Korea. han7537@hotmail.com *

## Aim

The first aim of this study was to determine if the self-adhesive cement (SAC) used for resin nanoceramic inlay affected internal gap at the tooth-restoration interface. The second aim was to investigate whether application of a universal adhesive before cementation affected internal gap.

## Material and Methods

Fifty-four Class I cavities were prepared on extracted 3rd molars. Cavities were optically scanned, and resin nanoceramic inlays were fabricated using Lava Ultimate block (3M ESPE). For the control group (n=6), the milled restoration was cemented using Panavia V5 (PV, Kuraray Noritake). For the experimental groups, the teeth were randomly divided into Groups I and II. Group I contained four subgroups using different self-adhesive cements (n=6 for each subgroup). For the subgroups of Group I, the inlays were placed with cement and dual-cured without pretreatment. For subgroup RX, RelyX U200 (3M ESPE) was used. For subgroup GL, G-cem LinkAce (GC) was used. For subgroups SC and MS, SmartCem 2 (Dentsply Caulk) and Multilink Speed (Ivoclar Vivadent) were used. Group II contained the same four subgroups in which inlays were cemented after application of a universal adhesive. After thermo-cycling, internal gap at the interface was measured using swept-source optical coherence tomography (SS-OCT, IVS-2000, Santec) imaging and statistically compared.

## Results

Internal gap at the tooth-restoration interface was different depending on the self-adhesive cement used (p<0.05). After application of a universal adhesive, each subgroup with universal adhesive showed similar or lower internal gap than that without adhesive (p<0.05).

## Conclusions

Internal gap at the interface of a resin nanoceramic inlay and tooth can be different depending on self-adhesive cement. For some self-adhesive cements, application of a universal adhesive before cement placement can make internal gap lower.

# Abstract: 03. Effect of remineralizing agents on shear bond strength of a universal adhesive to demineralized dentin

**Authors:** E. Meral

**Affiliations:** *Department of Restorative Dentistry, School of Dentistry, Hacettepe University, Ankara, Turkey. e.yildz@gmail.com*

## Aim

This study evaluated the effect of different remineralizing agents on the shear bond strength of a universal adhesive to demineralized dentin.

## Material and Methods

Forty human dentin specimens were prepared and subjected to pH cycling (8 h demineralization/16 h remineralization for 2 weeks). The specimens were randomly divided into four groups (n=10): three experimental groups treated with remineralizing agents—G1: a remineralizing gel containing calcium, phosphate, and magnesium (R.O.C.S. Medical Minerals); G2: a paste containing CPP-ACP (Tooth Mousse, GC, Tokyo, Japan); and G3: a foam containing hydroxyapatite (Curaprox Aligner Foam)—applied twice daily for 3 minutes over 5 days, and a control group left untreated. Resin composite cylinders (Omnichroma, Tokuyama, Tokyo, Japan) were bonded to the dentin using a universal adhesive (Bond Force II, Tokuyama, Tokyo, Japan), followed by shear bond strength testing. Data were analyzed using Kruskal-Wallis and Mann-Whitney U tests.

## Results

Statistically significant differences were observed between the groups (p<0.05). The G1 group (R.O.C.S. Medical Minerals) demonstrated significantly higher shear bond strength compared to other groups.

## Conclusions

Among the tested remineralizing agents, only R.O.C.S. Medical Minerals significantly improved the bond strength of a universal adhesive to demineralized dentin.

# Abstract: 04. The activity of the conservative pediatric dentistry services in primary care in Vallés oriental area (Barcelona)

**Authors:** E. Casals Peidró

**Affiliations:** *Catalan Health Institute, Barcelona, Spain. odontoleg@gmail.com*

## Aim

The conservative dentistry activity covered by the National Health System in Catalonia is provided in primary care centres. In the Granollers-Mollet Primary Care Service, two specific units were created to develop this activity (located in Granollers and Mollet) which we now call Specialised Pediatric Dentistry Services (SOEI). In late 2023 the overall offer was increased in Granollers with more working hours. The aim of this research is to evaluate the evolution of the activity in these two services as well and it's effects on patient engagement withinin a two-year period (January 2023-December 2024)

## Material and Methods

The activity noted (fillings and seals) has been individually exploited for all professionals who practise in the SOEI and use the eCAP-HES programme to reflect their activity. The delay in achieving an SOEI appointment throughout the months of the study period plus the amount of failed appointments have also been evaluated. Patient engagement was obtained exploiting data from scheduled visits in which the patient has not shown up. The average delay obtaining a dental appointment was evaluated per week calculating the amount of days between the day in which the appointment was made and the day it was scheduled eliminating all rescheduled appointments. Data was obtained from the average waiting time for all monday's patients. The average percentage of appointments where the patient did not show up were calculated on a weekly basis.

## Results

The results show the progressive increase in conservative activity due to the increase in the number of days of the SOEI staff Works as well as the increase in the number of patients visited per day from 8 to 9. The average monthly amount of fillings placed at the dental service in Granollers was 48,25 in 2023 and 77 in 2024. The average waiting list decreased from 7 to 3,5 months.

## Conclusions

The capacity to carry out conservative activity has progressively increased at the Granollers SOEI Service, the waiting list has decreased as well as the amount of failed appointments. Further increments on provision will better adjust offer to demand.

# Abstract: 05. Role of social media in aesthetic perception of dentistry students with and without clinical training

**Authors:** B. Dereli, O. Kardas, B. Alpay, E. Altin, M. Tiryaki

**Affiliations:** *Department of Restorative Dentistry, Faculty of Dentistry, Istanbul University, Istanbul, Turkey. beyza_dereli_@hotmail.com*

## Aim

Social media is a communication platform that enables individuals to create and share information with others. An online survey was conducted to evaluate whether the influence of social media differs among dental students with and without clinical training.

## Material and Methods

Volunteers were selected from dental school students. The exclusion criterion was non-use of social media platforms. A power analysis performed with the G*Power 3.1 program determined that a minimum of 321 questionnaires were required. The 25-question survey consisted of two parts. The first part gathered demographic information, while the second part explored students' use of social media and its relationship with dentistry. The pre-clinical education group consisted of 148 first- and second-year students, while the clinically trained group consisted of 202 third-, fourth-, and fifth-year students.

## Results

The survey revealed that 86.3% of dental students follow accounts related to dentistry, and 80.3% use social media to obtain information about treatments. However, only 24% of students trust the information obtained from social media. Additionally, 78.9% of students expressed interest in photos of aesthetic dental treatments shared on social media. A significant correlation was found between students aspiring to perform laminate veneer and whitening treatments in the future and the influence of social media on their interest in these procedures (p=0.002; p=0.034).

## Conclusions

It is concluded that social media increases interest in aesthetic dentistry in both groups. Students receiving clinical education believe they can perform dental treatments they see on social media with less practice time in their future careers (p=0.001).

# Abstract: 06. Effect of zirconia thickness, shade, and light-curing distance on irradiance and microhardness of a resin cement

**Authors:** D. Seo, Y.S. Choi, J.W. Park

**Affiliations:** *Department of Conservative Dentistry, School of Dentistry Seoul National University, Seoul, South Korea. dgseo2@gmail.com*

## Aim

This study investigated the effect of distance, zirconia thickness, and shade on the irradiance and microhardness of a dual-cure resin cement using two light-curing units. It also examined whether extended exposure time enhanced microhardness through different thicknesses and shades of zirconia.

## Material and Methods

Two light-curing units, the Eliparª DeepCure-S (3M) and the VALO Cordless (Ultradent), were tested for 10 seconds (standard mode) and 3 seconds (Xtra mode), respectively. Irradiance measurements (n=3) were taken at different distances (0.0 to 6.0 mm), and through zirconia discs (0.5 to 4.0 mm, shades A2 and A3) using a CheckMARC radiometer (BlueLight Analytics Inc.). Dual-cure resin cement samples (SeT PP, SDI) were light-cured under the same conditions (n=3). Microhardness was measured after 24 hours using a Vickers hardness tester (HM-220B, Mitutoyo). Additional samples (n=3) were tested with extended exposure times under 0.5 to 2.0 mm zirconia discs to match the irradiance at 0.0 mm. Statistical analyses included Kruskal-Wallis tests with Dunn's post-hoc tests and two-way ANOVA on ART-transformed data (α<0.05).

## Results

For both light-curing units, increasing distance significantly reduced irradiance from 5.0 mm and microhardness from 3.0 mm. Increasing zirconia thickness significantly decreased irradiance and microhardness starting at 0.5 mm. A3 shade showed lower irradiance and microhardness than A2 (p<0.05). Extended exposure times improved microhardness, compensating for energy losses from thicker zirconia and darker shades.

## Conclusions

Distance, zirconia thickness, and shade decreased microhardness of the resin cement, even with a high-irradiance unit. These effects were more pronounced with darker shades and thicker zirconia. Extended exposure time successfully compensated for these losses of energy and microhardness.

# Abstract: 07. Influence of different caries removal methods on dentin bond strength

**Authors:** S. Aznar León, A. Otero Ávila, P. González Villafranca, C. Lucena Martín, C. Benavides Reyes

**Affiliations:** *Master’s Degree in Aesthetic Multidisciplinary Dentistry, University of Granada, Granada, Spain. sofia98aznar@hotmail.com*

## Aim

To compare the shear bond strength to dentin after caries removal with different methods: Brix300, rounded carbure bur and SmartBurs polymer bur.

## Material and Methods

Healthy teeth and decayed teeth were assigned to two control groups: (1) healthy and (2) pH-cycling; and three experimental caries removal groups: (3) carbure Bur, (4) Polymer Bur (SmartBur), and (5) Brix3000. After the different protocols, the adhesive was applied, and it was filled with incremental technique. Subsequently, the samples were sectioned into bars with a cross-sectional area of 1 mm. The bars were mounted in a modified Bencor Multi-T testing support with cyanoacrylate. The shear bond strength was determined using an Instron 3345 and the mode of failure was observed: adhesive, cohesive or mixed. Means and standard deviations were calculated, and the one-factor ANOVA test was applied to determine the differences between groups. In addition, the differences between groups were determined with Tukey's test.

## Results

The healthy group was the one that obtained higher values compared to the rest of the groups, this difference being statistically significant with the SmartBurs and Brix3000 groups. The distribution of the types of failures was different according to the type of technique used.

## Conclusions

Both, the SmartBurs and Brix3000 groups, achieve a similar shear bond strength that that of the layer affected by caries, being the methods less aggressive than conventional bur treatment.

# Abstract: 08. Dehydration effect on tooth colour -in vivo study-

**Authors:** P. Hoyo Liddle, E. Rosel Gallardo, V. Valenzuela Triviño, L. Molina Hernández, G. Martín Martín, C. Lucena Martín

**Affiliations:** *Master’s Degree in Aesthetic Multidisciplinary Dentistry, University of Granada, Granada, Spain. hoyopatricia@gmail.com*

## Aim

Determine if the absolute isolation of the operating field affects tooth colour, quantify the magnitude and direction of the chromatic change as a function of the isolation time and evaluate the time necessary for the teeth to recover their initial colour.

## Material and Methods

The colour of 26 central incisors and 26 canines was measured with an Easyshade¨ spectrophotometer under basal conditions and after isolation with a rubber dam, at minutes 5, 7, 10, 20 and 30 of dehydration. The isolation was removed, and the colour was recorded at 2, 4 and 60 minutes, and at 24 hours of rehydration. For each tooth and time, the coordinates L*, a*, b*, C* and h* were obtained. From them, the colour differences (ΔE00 and ΔE*ab) between the basal conditions and the different measurement times were calculated, interpreting them with respect to their respective thresholds of perceptibility (PT) and acceptability (AT) 50:50%.

## Results

The L*a*b* and C* coordinates of incisors and canines increased during the dehydration time and decreased with rehydration, while the tone angle (h* showed an opposite trend. Colour differences (ΔE00 and ΔE* ab) increased progressively with dehydration time, exceeding the acceptability threshold at 5 minutes of dehydration. During rehydration, ΔE values decreased, being clinically acceptable (<AT) after 30 minutes of rehydration, but perceptible after 24 hours.

## Conclusions

Dehydration-induced chromatic changes can cause errors during colour selection and verification in restorative procedures.

# Abstract: 09. Fracture resistance in endodontically treated teeth according to the type of restoration

**Authors:** A. Bailón Elvira, A.B. Muñoz Puerto, A. Costas Soto, C. Benavides Reyes, C. Lucena Martín

**Affiliations:** *Master’s Degree in Aesthetic Multidisciplinary Dentistry, University of Granada, Granada, Spain. abailon@correo.ugr.es*

## Aim

The objective of this systematic review was to evaluate the effect of different direct and indirect restoration techniques and Material on endodontically treated teeth (ETT) in order to determine which options provide greater fracture resistance and clinical longevity.

## Material and Methods

A literature search was conducted in Scopus, Medline (PubMed), Cochrane, and Embase databases, using descriptors related to endodontically treated posterior teeth and direct or indirect restorations. The results were limited to articles published in the last ten years in English or Spanish.

## Results

Out of an initial total of 292 articles identified, 44 were selected for full-text review after duplicate removal and manual screening by two researchers. A total of 21 studies met the inclusion criteria: 18 in vitro studies and 3 in vivo studies.

## Conclusions

1. Cuspal coverage is essential to improve fracture resistance in endodontically treated teeth. 2. There is insufficient evidence to demonstrate clear differences between direct and indirect restorations in terms of fracture resistance. 3. Additional clinical studies are needed to compare different restoration techniques using ceramics and hybrid Material in endodontically treated posterior teeth.

# Abstract: 10. Chemical comparison of dentin after caries removal using chemical-mechanical techniques and classical technique

**Authors:** C. García Bedmar, B. Vilchez de la Fuente, A. Costas Soto

**Affiliations:** *Master’s Degree in Aesthetic Multidisciplinary Dentistry, University of Granada, Granada, Spain. celiaegb685@gmail.com*

## Aim

Chemical comparison of dentin after caries removal using chemical-mechanical techniques and classical technique with rotary instruments.

## Material and Methods

Twelve permanent human molars with caries lesions were longitudinally sectioned in an occlusal-apical direction, dividing the caries lesion in two. Each half was randomly assigned to an experimental elimination group: Brix3000 or bur. After the caries removal protocol, they were analyzed by X-ray diffraction, attenuated total reflection infrared spectroscopy and Masson's trichrome staining. The data were analyzed by ANOVA and the Bonferroni test. A significance level of p<0.05 was established.

## Results

The relative amount of mineral to organic matrix (PO4 / Amide I) and the total amount of carbonate in relation to the organic matrix (CO3 / Amide I) was higher in the control group, followed by the bur group and Brix3000. The mineral crystallinity index and crystalite size measured by X-ray diffraction is higher in healthy dentin, obtaining statistically significant differences with the rest of the groups. The size of the crystallite in the remaining dentin after the bur and after removal with Brix3000, did not show statistically significant differences.

## Conclusions

Although more studies are needed to corroborate the chemical and mechanical characteristics of the substrate after caries removal with Brix3000, we can conclude that this product removes the caries-infected layer effectively and less aggressively than conventional strawberry treatment.

# Abstract: 11. Voids, wear and 3D adaptation in DME technique using different Material before and after cyclic fatigue

**Authors:** M. Biondi, A. Baldi, A. Comba, N. Scotti

**Affiliations:** *Dental School, University of Turin, Turin, Italy. michael.biondi26@gmail.com*

## Aim

The aim of this study is to evaluate voids, restoration wear and three-dimensional adaptation of different Material before and after cyclic fatigue in an in-vitro simulated DME using micro-CT.

## Material and Methods

Intact human molars will be selected and stored in water. A single operator will perform class II MOD cavity preparations on each tooth, with the following parameters: 4mm bucco-oral, 3mm mesio-distal for mesial and distal box; cervical margin 1mm above CEJ for the mesial and 1mm below for the distal one. The adhesive procedure will be performed with circumferential matrixes and a universal adhesive with selective enamel etching. Specimens will be divided in three groups: G1: A pre-heated bulk composite (VisCalor Bulk,VOCO) with a 2mm-thick horizontal layer. G2: A nano-hybrid ormocer (Admira Fusion, VOCO) with 2mm-thick horizontal layers. G3: A highly-filled flowable composite (GrandioSo Heavy Flow, VOCO) with 2mm-thick horizontal layer. Baseline marginal adaptation and voids of each restoration will be evaluated using a Micro-CT. After that, a chewing simulator (CS-4.4 Mechatronik) will be used for fatigue-cycling mechanical aging of the specimens (occlusal load=50 N, 200.000 cycles). Then, to reveal interfacial gap progression and wear, specimens will be subjected to a second scan with the same protocol. Data were recorded and evaluated with ANOVA test, setting p<0.05.

## Results

No statistically significant differences were found between tested Material in terms of voids and adaptation of the restoration. Small differences were highlighted in terms of wear, with G3 performing slightly worse than G1 and G2 (p=0.04). Cyclic fatigue always resulted in a degradation of the interface, but not significant in terms of statistical analysis, probably due to low number of cycles.

## Conclusions

All Material performed similarly in terms of voids, wear and adaptation. Further studies are necessary to confirm such data with longer aging periods.

# Abstract: 12. Universal composites: A guide to understanding and indicating them

**Authors:** S. Treviño-Díaz, V. Vásquez, M. Carmona, M. Flores, B. Baracco, C. Martín Carreras-Presas

**Affiliations:** *European University of Madrid, Madrid, Spain. sofia_l131@hotmail.com*

## Aim

Universal composites evolved over the years, improving their properties such as wear resistance, handling and polishing capacity. These Material are indicated for anterior and posterior restorations, in addition to simplifying the shades of colors in contrast to conventional composites. The general objective is to better know the characteristics and properties of several universal composites: Filtek Universal (Solventum), Tetric Evoceram(Ivoclar Vivadent), A'Chord (GC), Essentia Universal (GC), Omnichroma (Tokuyama), Charisma Diamond One (Kulzer), Venus Diamond (Kulzer) and Estelite Sigma Quick (Tokuyama).

## Material and Methods

A bibliographic review was carried out by consulting PubMed, Medline and CRAI Dulce Chac—n databases. Scientific, research and informative articles were accepted; as well as reviews of the most relevant literature published in recent years (2007-to date), in English and Spanish. Articles related to the topic were included and those whose summaries were not available or did not have open access were discarded.

## Results

A total of 24 articles were found, related to the topic, of which 3 observational studies (1 cohort, 1 case, 1 cross-sectional); 14 experimental studies (10 laboratory, 4 clinical trials); 1 comparative analysis of scientific literature; 4 informative analyses; 1 narrative review or clinical guideline; 1 literature review. The universal composites especially indicated for the anterior sector due to their polishing properties were Omnichroma, Estelite Sigma Quick, Venus Diamond, Charisma Diamond One and A'Chord, although Filtek Universal showed the highest surface gloss. Essentia Universal showed the lowest surface roughness and A'Chord the least stickiness.

## Conclusions

Also, the most indicated composites for the posterior sector according to their resistance and hardness were Filtek Universal, followed by A'Chord and, finally, Tetric Evoceram, Essentia Universal and Estelite Sigma Quick. Lastly, Venus Diamond is the best in regard of stiffness.

# Abstract: 13. Assessing the relation between depression, anxiety, and bruxism in patients at dental and physical therapy clinics: A survey study

**Authors:** E. Kitin, E. Yildiz, H.K. Gun, N. Alaca, Y. Sevda Ozel

**Affiliations:** *Department of Restorative Dentistry, Faculty of Dentistry, Istanbul University, Istanbul, Turkey. dt.eminekitin@hotmail.com*

## Aim

The aim of this study was to investigate the relationship between the presence of bruxism and the levels of depression and anxiety in patients applying to dental and physical therapy clinics for various reasons. Understanding this relationship may enhance the management of bruxism by integrating psychological assessments.

## Material and Methods

A cross-sectional survey was conducted involving 439 patients from the Istanbul University, Faculty of Dentistry (n:198) and the Acibadem University Physical Therapy and Rehabilitation Center (n:241). Participants completed a structured questionnaire that included validated scale called hospital anxiety and depression scale (HADS), along with a self-assessment for bruxism categorized as either present or absent. Anxiety and depression levels were rated as normal, borderline, or abnormal. Data were collected over a three-month period, and evaluated using non -parametric Mann Whitney U tests and chi-square tests, performed through SPSS software.

## Results

Among the 439 participants, the prevalence of self-reported bruxism was identified in 45,1 % of respondents. No significant difference was found in the prevalence of bruxism between patients from both clinics. While patients at both centers exhibited similar levels of anxiety, depression levels were statistically significantly higher in dental clinic patients compared to those attending the physical therapy center (p=0.02<0.05). In the dental clinic, it was determined that as anxiety and depression levels increased, so did the reporting of bruxism. Conversely, in the physical therapy center, a rise in anxiety levels was associated with an increase in bruxism reports; however, patients with borderline depression levels reported higher instances of bruxism.

## Conclusions

The findings highlight the complex relationship between bruxism, anxiety, and depression among patients in dental and physical therapy clinics. These results suggest that psychological factors may significantly contribute to bruxism. Therefore, a multidisciplinary approach is recommended for effective bruxism management.
